# Going beyond the borders: pyrrolo[3,2-*b*]pyrroles with deep red emission†

**DOI:** 10.1039/d1sc05007a

**Published:** 2021-11-22

**Authors:** Mariusz Tasior, Paweł Kowalczyk, Marta Przybył, Małgorzata Czichy, Patryk Janasik, Manon H. E. Bousquet, Mieczysław Łapkowski, Matt Rammo, Aleksander Rebane, Denis Jacquemin, Daniel T. Gryko

**Affiliations:** Institute of Organic Chemistry, Polish Academy of Sciences Kasprzaka 44–52 01-224 Warsaw Poland dtgryko@icho.edu.pl; Faculty of Chemistry, Silesian University of Technology Strzody 9 44-100 Gliwice Poland mieczyslaw.lapkowski@polsl.pl; CEISAM Lab—UMR 6230, CNRS, University of Nantes Nantes France Denis.Jacquemin@univ-nantes.fr; Centre of Polymer and Carbon Materials, Polish Academy of Sciences Curie-Sklodowskiej 34 41-819 Zabrze Poland; National Institute of Chemical Physics and Biophysics Tallinn Estonia; Department of Physics, Montana State University Bozeman MT 59717 USA arebane@montana.edu

## Abstract

A two-step route to strongly absorbing and efficiently orange to deep red fluorescent, doubly B/N-doped, ladder-type pyrrolo[3,2-*b*]pyrroles has been developed. We synthesize and study a series of derivatives of these four-coordinate boron-containing, nominally quadrupolar materials, which mostly exhibit one-photon absorption in the 500–600 nm range with the peak molar extinction coefficients reaching 150 000, and emission in the 520–670 nm range with the fluorescence quantum yields reaching 0.90. Within the family of these ultrastable dyes even small structural changes lead to significant variations of the photophysical properties, in some cases attributed to reversal of energy ordering of alternate-parity excited electronic states. Effective preservation of ground-state inversion symmetry was evidenced by very weak two-photon absorption (2PA) at excitation wavelengths corresponding to the lowest-energy, strongly one-photon allowed purely electronic transition. π-Expanded derivatives and those possessing electron-donating groups showed the most red-shifted absorption- and emission spectra, while displaying remarkably high peak 2PA cross-section (*σ*_2PA_) values reaching ∼2400 GM at around 760 nm, corresponding to a two-photon allowed higher-energy excited state. At the same time, derivatives lacking π-expansion were found to have a relatively weak 2PA peak centered at *ca.* 800–900 nm with the maximum *σ*_2PA_ ∼50–250 GM. Our findings are augmented by theoretical calculations performed using TD-DFT method, which reproduce the main experimental trends, including the 2PA, in a nearly quantitative manner. Electrochemical studies revealed that the HOMO of the new dyes is located at *ca*. −5.35 eV making them relatively electron rich in spite of the presence of two B^−^–N^+^ dative bonds. These dyes undergo a fully reversible first oxidation, located on the diphenylpyrrolo[3,2-*b*]pyrrole core, directly to the di(radical cation) stage.

## Abstract 2

Ladder-type heterocycles encompassing two B^−^–N^+^ dative bonds possess intense green to red emission, large 2PA cross-sections and superb photostability.

## Graphical abstract

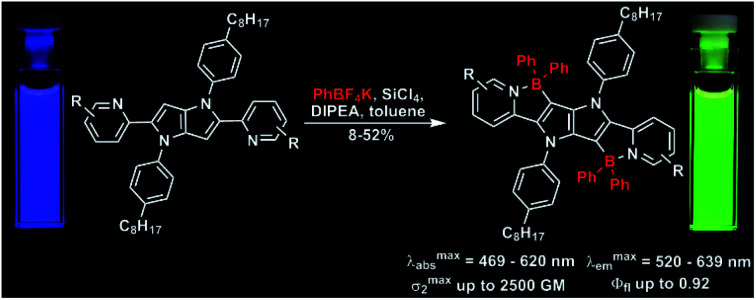

## Introduction

In recent years, boron has found new distinct roles in materials science, being incorporated in various polycyclic aromatic hydrocarbons (PAHs).^1–7^ It has been discovered that doping with three-coordinate, sp^2^-hybridized boron has the most pronounced effect on aromatic systems among main group elements, due to its low Pauling electronegativity and enhanced π-conjugation resulting from its vacant p_*z*_ orbital. As a consequence, in such systems boron atoms can act simultaneously as π-electron acceptors and σ-electron donors. The fascinating properties of the materials obtained in such a way (so-called B-PAHs), such as strong fluorescence^8–11^ and enhanced charge-transport characteristics,^12,13^ has resulted in a wide range of applications in optoelectronics, including materials for organic light-emitting diodes (OLEDs)^14–16^ circularly polarized luminescence,^17^ organic photovoltaics (OPVs)^18^ and organic field-effect transistors (OFETs)^19–21^ as well as materials for electrodes in lithium batteries.^22^ Much attention has also been paid to the B–N/O isosteres of PAHs, *i.e.* compounds in which two adjacent carbons in a π-conjugated core have been replaced by one boron and one N/O atom.^23–28^ Of particular importance are π-conjugated systems containing a B–N covalent bond, B←N coordination bond^29^ and N–B←N motif, as evidenced by growing number of reports dealing with BN-embedded heteroacenes^29–32^ and BODIPY analogues,^33–37^ mostly due to their excellent performance in OLEDs^38*a*^ and OPVs.^38*b*,*c*^ They were also computed to possess inverted singlet-triplet gap.^39^ In such systems, the N–B–N motif not only constrains the π-conjugated skeleton in a coplanar fashion, but also considerably lowers the LUMO level, making the core of the dye a strong acceptor.

**Fig. 1:**
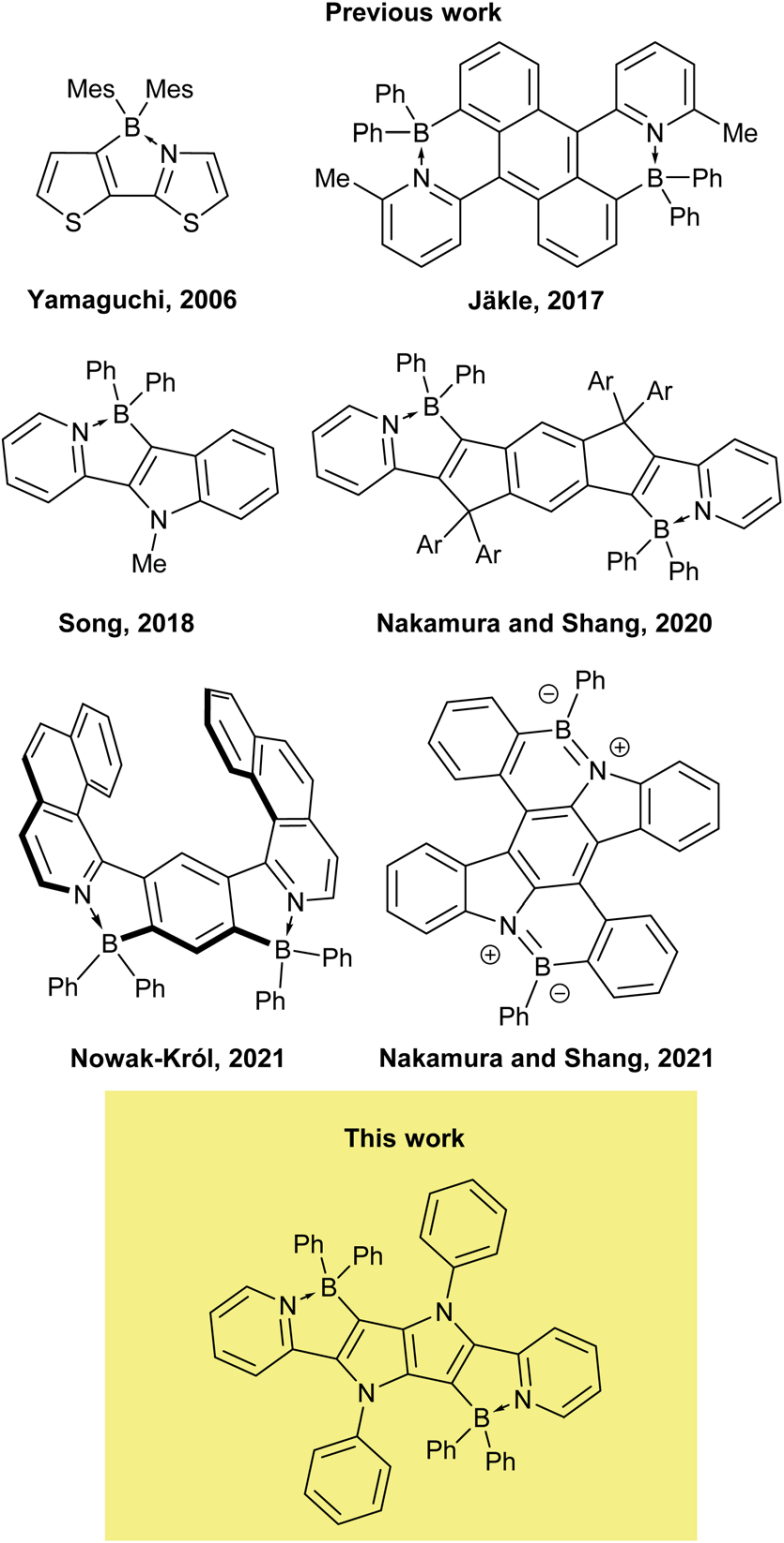
Chemical structures of the representative dyes possessing a B←N coordination motif.

1,2,4,5-Tetraarylpyrrolo[3,2-*b*]pyrroles (TAPPs)^40^ are aza-analogues of well-known thieno[3,2-*b*]thiophenes.^41^ Recent synthetic breakthroughs,^42^ prompted their application in research related to studying symmetry breaking in the excited state,^43^ solvatofluorochromism,^43*b*,*c*^ direct solvent probing *via* H-bonding interactions^44^ photochromic analysis of halocarbons^45^ organic light emitting diodes^46^ resistive memory devices,^47^ bulk heterojunction organic solar cells,^48^ dye-sensitized solar cells,^49^ aggregation-induced emission^50^ and MOFs.^51^ Furthermore, the very high reactivity at positions 3 and 6 of the pyrrolo[3,2-*b*]pyrrole core makes them very convenient starting materials for the construction of ladder type heterocycles. Taking advantage of this feature, many reports have been published on the synthesis and properties of TAPP-based PAHs,^52^ however, there has only been one successful synthesis of BN-embedded TAPPs (Fig. 1).^53^ The resulting dyes, containing boron atoms incorporated into six-membered cycles, exhibited very high absorption coefficients and strong fluorescence, both in solution and the solid state, with very small Stokes shifts.

**Scheme 1:**
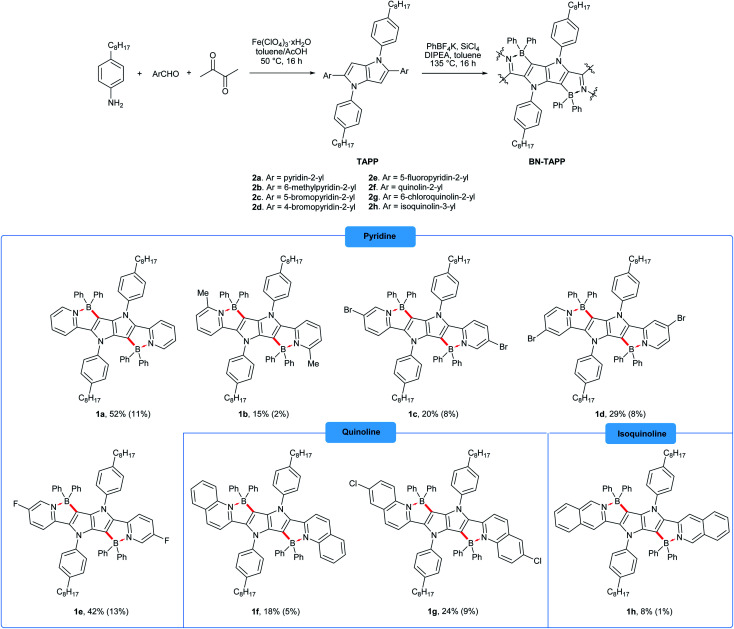
The synthesis of four-coordinate boron-containing pyrrolo[3,2-*b*]pyrroles. Isolated yields are given. The overall yields (after two steps) are given in parenthesis.

Although the combination of suitable photophysical properties have made TAPPs and fused TAPP analogues popular chromophores for a range of applications, shifting their emission to the red region of the visible spectrum has proved difficult. We have sought to address this issue in this work. Here, we have considered the reaction of pyrrolo[3,2-*b*]pyrroles bearing 2-pyridil substituents in positions 2 and 5 (C=N bonds in N-heteroaromatic rings are effective in coordinating with boron moieties) with an appropriate boron source (Scheme 1). The resultant B←N=C five membered chelate ring fuses the pyrrolopyrrole and pyridine substituents together and effectively fixes the π-conjugated framework in a coplanar fashion resulting in the formation of a unique chromophore, which perfectly fits into the current intense research on BN-embedded heteroacenes.

## Results and discussion

### Design and synthesis

**Scheme 2:**
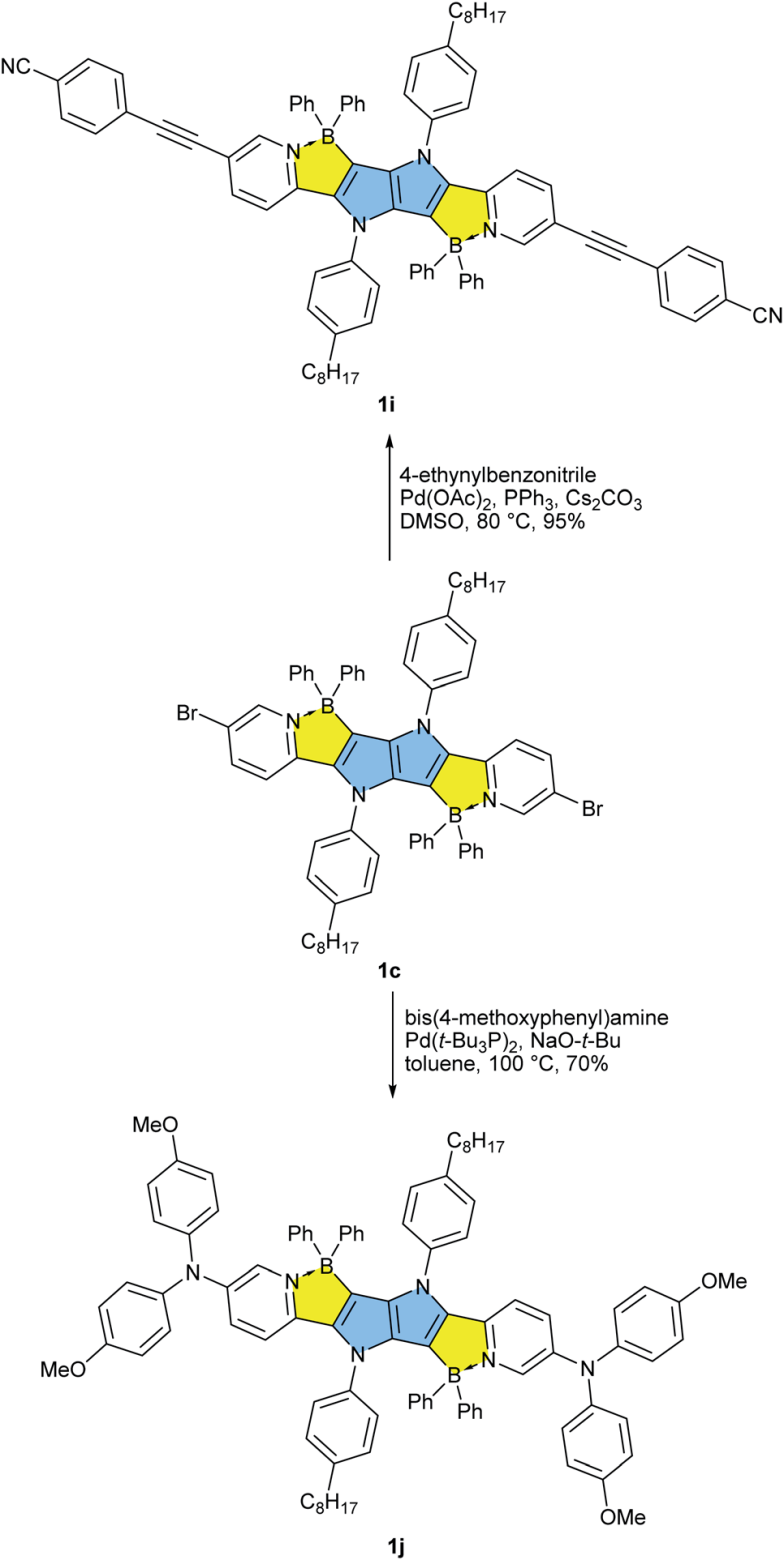
Post functionalization of BN-TAPP 1c leading towards dyes 1i and 1j.

**Scheme 3:**
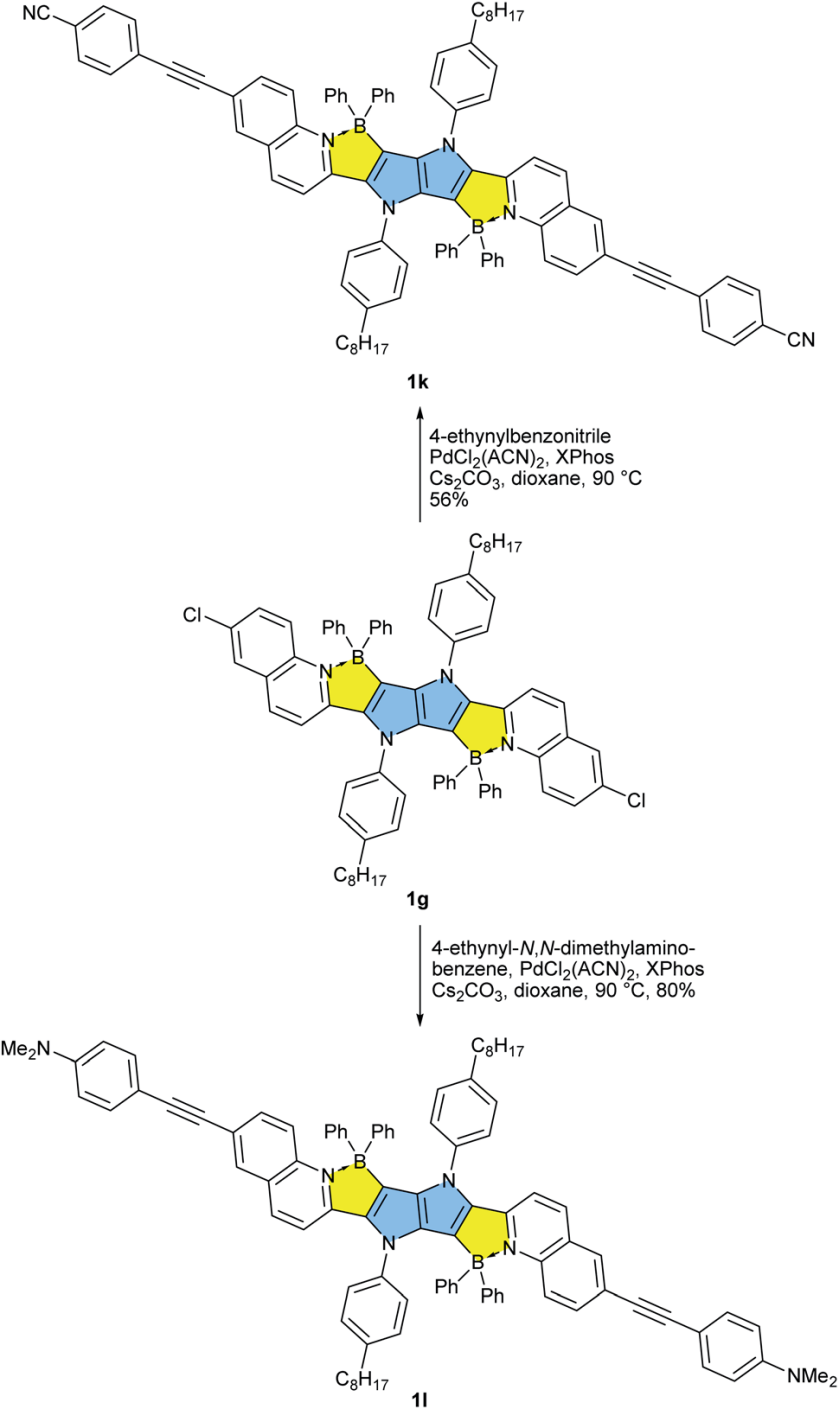
Post functionalization of BN-TAPP 1g leading towards dyes 1k and 1l.

Our approach towards new BN-embedded heterocycles capitalizes on pyrrolo[3,2-*b*]pyrroles' intrinsically high reactivity at positions 3 and 6, and on the straightforward access to derivatives bearing pyridyl substituents at positions 2 and 5 (Scheme 1). The necessary TAPPs 2a–2e were obtained in good yields from appropriate formylpyridines and 4-octylaniline using our optimized multicomponent condensation (see ESI† for details).^42*a*^ TAPPs 2f–2h were formed in lower, but sufficient yields from appropriate quinoline- and isoquinoline-derived aldehydes. We anticipated that the planar structure of BN-embedded heterocycles would result in extensive π-stacking, therefore long alkyl chains were installed on the *N*-linked aryl substituents in order to secure good solubility of the final products. In the next step, we planned to apply a well-known strategy consisting of treatment of the parent pyrrolopyrrole with BBr_3_ in the presence of base, however, this led to complete decomposition of the starting material. An alternative route was to use diarylchloroboranes, but this seemed difficult due to the limited availability of such reagents. Fortunately, Song and co-workers recently developed a straightforward cascade B–Cl/C–B cross-metathesis and C–H bond borylation procedure,^54^ which we successfully used in the synthesis of the desired four-coordinated boron-containing pyrrolo[3,2-*b*]pyrroles 1a–1h (BN-TAPPs) (Scheme 1). According to Song's findings, in this procedure aryltrifluoroborate reacts with SiCl_4_ to form aryldichloroborane in highly selective manner, which then reacts with another molecule of aryltrifluoroborate to give diphenylchloroborane. Subsequent pyridine directed electrophilic aromatic borylation leads to four-coordinate triarylborane. The yields in the last step are greatly improved when sterically hindered organic base is applied. Importantly, this simple procedure can be conveniently applied to starting materials containing both pyridyl and quinolinyl substituents, and it tolerates the presence of halogens in the starting materials. Consequently, we prepared a few BN-embedded TAPPs substituted with various halogens and submitted them to Sonogashira or Buchwald–Hartwig cross-couplings, in order to further expand the π-system. Although the outcomes of these reactions were not obvious due to the presence of reactive boron and halogen substituents, the desired compounds 1i–1l (Schemes 2 and 3) were isolated in acceptable yields.

### X-ray analysis

**Fig. 2:**
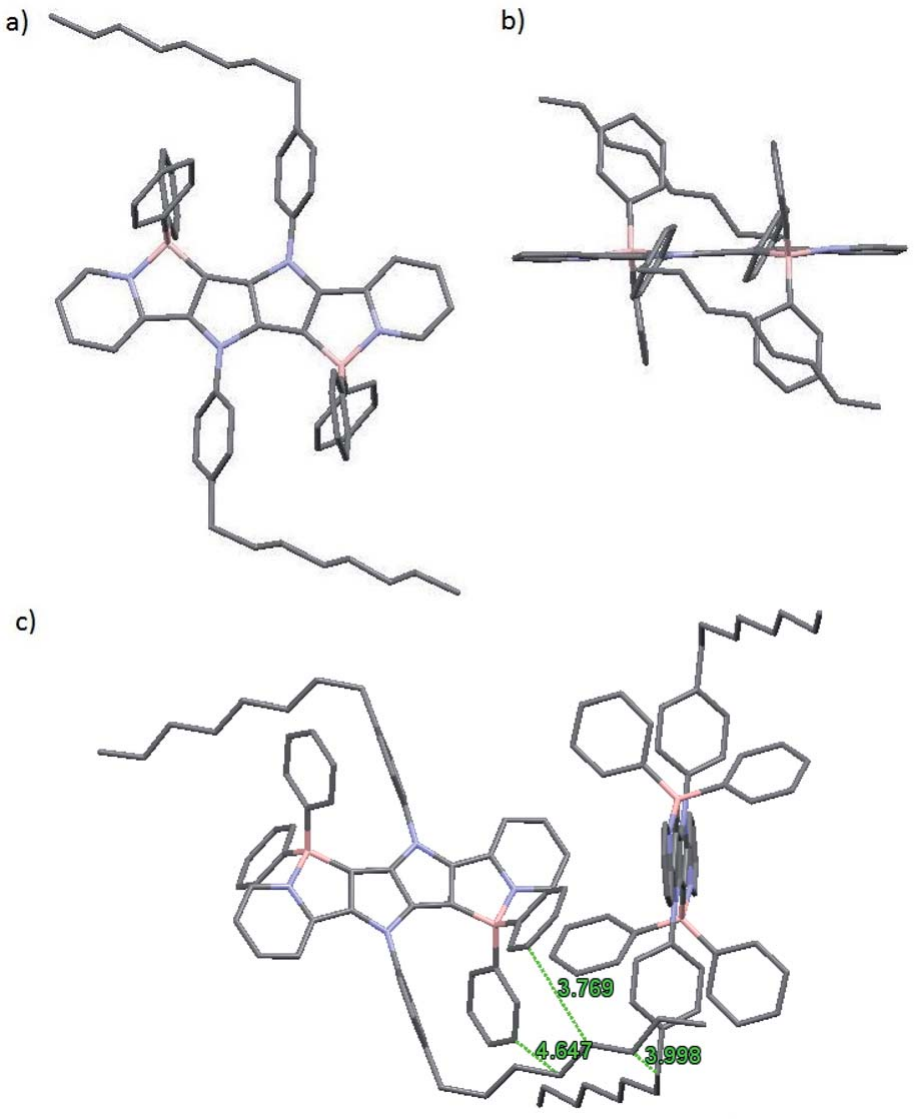
(a) Top view, (b) side view and (c) crystal packing of 1a as determined from X-ray crystallography (CCDC). Hydrogen atoms were omitted for were omitted for clarity. Distances are given in angstroms.

The molecular structure of 1a was determined by X-ray crystallography. Single crystals of 1a suitable for analysis were obtained at ambient temperature by slowly diffusing hexanes into a tetrahydrofuran solution. Dye 1a crystallizes in the monoclinic space group *P*2_1_/*c*, and the unit cell comprises of two molecules (Fig. 2). The pyrrolo[3,2-*b*]pyrrole core adopts a perfectly planar structure (180° N–C–C–N torsion angle) and the peripheral pyridine rings are both twisted by 5° from this plane.

Benzene rings attached to nitrogen atoms of the pyrrolopyrrole core are twisted by 59° and 61°, while phenyl substituents attached to boron atoms are twisted by 83° and 78°. The lengths of the newly formed B–C bonds are 1.6 Å, which is a slightly shorter value than those reported for related compounds and suggests a stronger bond,^35,55^ while the lengths of the B–N bonds are 1.63 Å, which is similar to the B–N bond length found in analogous compounds^55^ and BODIPY-type molecules.^56^ Van der Waals interactions govern the crystal packing, with the main structural motif being the interaction of bent C8 aliphatic chain with two phenyl substituents attached to the boron atom adjacent to the same molecule and then with the octylphenyl substituent attached to the nitrogen atom of the neighboring molecule. No obvious π-stacking interactions are observed, probably due to the orientation of phenyl substituents on the boron atom that prevents these heteroacenes with extended π systems from approaching each other.

**Fig. 3:**
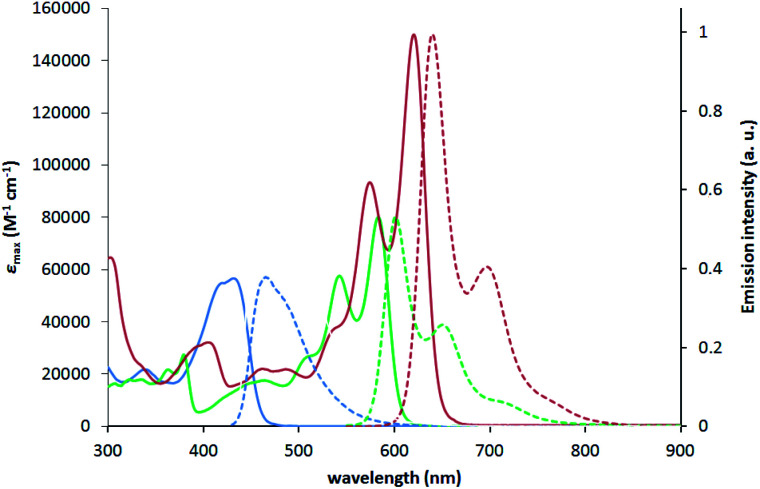
Absorption (solid lines) and normalized emission (dashed lines) of compounds 2f (blue), 1f (green) and 1k (red) measured in toluene.

To characterize the photophysical properties of the novel dyes we conducted a multipronged campaign. The optical properties of TAPPs 2a–2h correspond very well to those reported earlier for this class of dyes (see ESI† for details).^42*a*^ The incorporation of boron into the TAPPs backbone, however, brings spectacular changes in their optical properties (Table 1 and Fig. 3). The absorption maxima for compounds 1a–1g are bathochromically shifted by 122–155 nm when compared to parent TAPPs. Only for compound 1h, a somewhat lower, but still remarkable 68 nm red-shift of the absorption maxima was observed, accompanied by an unusual broadening of the band shape and by a low intensity band between 600 and 700 nm (see ESI†). Theoretical calculations indicate that the latter is most likely not connected to the isolated 1h structure (see below), whereas preliminary measurements did not detect formation of aggregates or other related structures. Similarly, the emission maxima were red-shifted by over 100 nm, with very small Stokes shifts of less than 1000 cm^−1^ and large fluorescence quantum yields for most of the dyes. These results clearly indicate a rigidifying effect of the boron component thanks to the character of boron atoms. Noticeably, in the case of compounds 1k and 1l, in which the π-systems were further expanded, the barrier of 600 nm for both absorption and emission maxima was broken, which means that these are the first examples of pyrrolo[3,2-*b*]pyrrole derivatives absorbing and emitting in the deep-red region. For dyes 1i, 1k and 1l, exceptionally high extinction coefficients were observed, exceeding 100 000 M^−1^ cm^−1^, together with large *Φ*_fl_. This combination of properties is particularly rare for deep red-emitting dyes due to additional deactivation pathways by vibrational relaxation (*energy gap law*). A very weak emission was detected at 670 nm for dye 1h. The excitation spectrum however, is not consistent with the absorption profile, which points at potential more complex underlying photophysics to be addressed in subsequent investigations. When compared to recently published, structurally related B/N-doped *p*-arylenevinylene chromophores,^35^ BN-TAPPs absorb and emit at much longer wavelengths, with the difference being most noticeable for the derivatives bearing similar, unsubstituted quinoline moieties (63 nm and 80 nm bathochromic shift in the absorption and emission maxima, respectively). *p*-Arylenevinylene analogues of BN-TAPPs exhibit higher fluorescence quantum yields which are close to being quantitative, although the latter compounds are characterized by much higher molar extinction coefficients, which overall results in a similar emissive brightness for these two classes of organic chromophores.

**Table 1:** Spectroscopic properties of dyes 1a-1l in toluene

Compound	*λ* ^max^_abs_ [nm]	*ε*_max_ [M^−1^ cm^−1^]	*λ* ^max^_em_ [nm]	Stokes shift [cm^−1^]	*Φ*_fl_	2*λ*^max^_abs_ [nm]	*λ* ^max^_2PA_ [nm]	*σ* ^max^_2PA_ [GM]	*σ* ^max^_2PA_ *Φ*_fl_ [GM]
1a	502	42 500	521	730	0.78^a^	1004	650	104	80
	474	40 100				948			
	380	26 800				760			
1b	504	52 500	520	610	0.77^a^	1008	660	102	80
	476	44 100				952			
	406	18 200				812			
1c	528	42 800	553	860	0.28^a^	1056	670	188	50
	499	42 700				998			
	397	34 000				794			
1d	513	48 700	533	730	0.42^a^	1026	660	176	70
	484	44 600				968			
	392	29 200				784			
1e	520	20 900	557	1280	0.28^a^	1040	640	93	25
	494	25 500				988			
	391	34 500				782			
1f	583	80 000	601	510	0.78^b^	1166	940	200	160
	542	57 600				1084			
	379	27 600				758			
1g	598	80 900	616	490	0.75^b^	1196	920	270	200
	555	57 900				1110			
	388	29 800				776			
1h	469	84 100	—c	—c	—c	—	—c	—	—
1i	566	134 000	589	690	0.92^b^	1132	800	1400	1300
	431	90 700				862			
	420	28 200				840			
1j	563	62 900	590	810	0.75^b^	1124	790	590	440
	533	51 600				1066			
	424	50 500				848			
1k	620	150 000	639	480	0.78^b^	1240	760	2500	2000
	574	93 800				1148			
	536	37 500				1072			
1l	618	135 000	639	530	0.88^b^	1236	870	1800	1600
	573	81 300				1146			
	534	29 600				1068			

a Determined with fluorescein in NaOH (0.01 M) as a standard.

b Determined with cresyl violet perchlorate in MeOH as a standard.

c Fluorescence of dye 1h was not investigated in 2PA due to the inconsistency of the one-photon fluorescence excitation results.

### Photostability

**Fig. 4:**
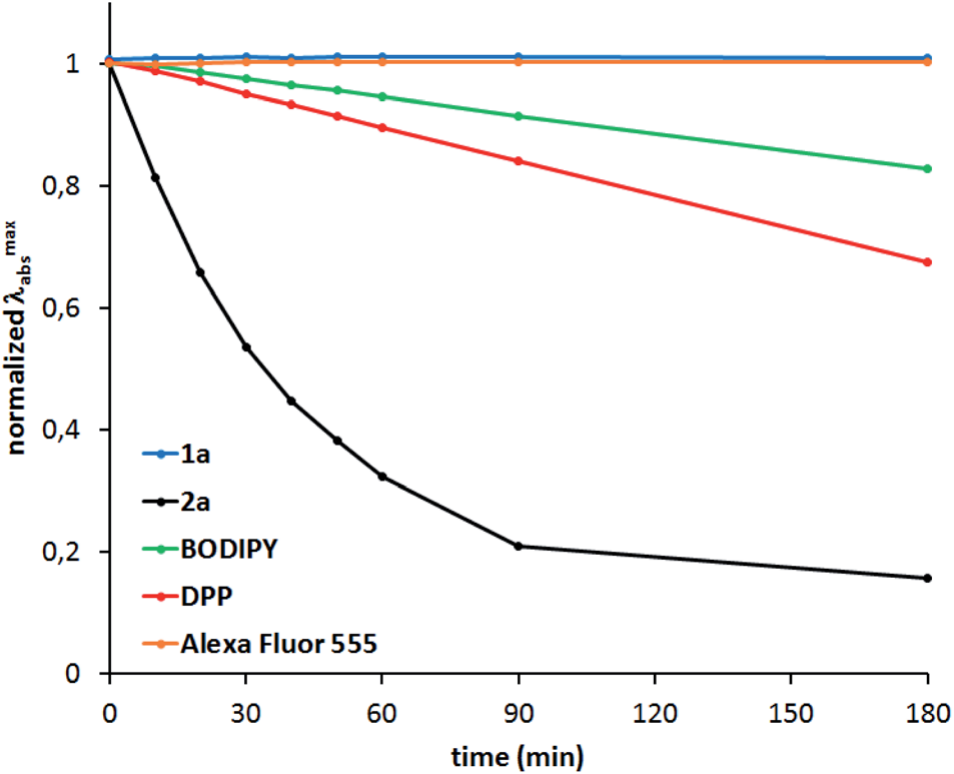
Photostability of 1a and 2a compared to a BODIPY (BODIPY 493/503), DPP (2,5-dioctyl-3,6-bis(3,4,5-trimethoxyphenyl)pyrrolo[3,4-*c*]pyrrole-1,4(2*H*,5*H*)-dione) and Alexa Fluor 555 measured using a collimated light source from a 300 W Xe lamp.

Photostability tests were carried out for compounds 1a and 2a as representative examples (Fig. 4). TAPPs, which generally exhibit very poor stability under irradiation, acquire remarkable photostability upon incorporation of boron, as demonstrated by comparing the properties of 1a with common dyes, such as BODIPY and diketopyrrolopyrroles. Even prolonged exposure to a strong light source did not cause any noticeable changes in the absorption spectrum of 1a.

### Two-photon absorption

**Fig. 5:**
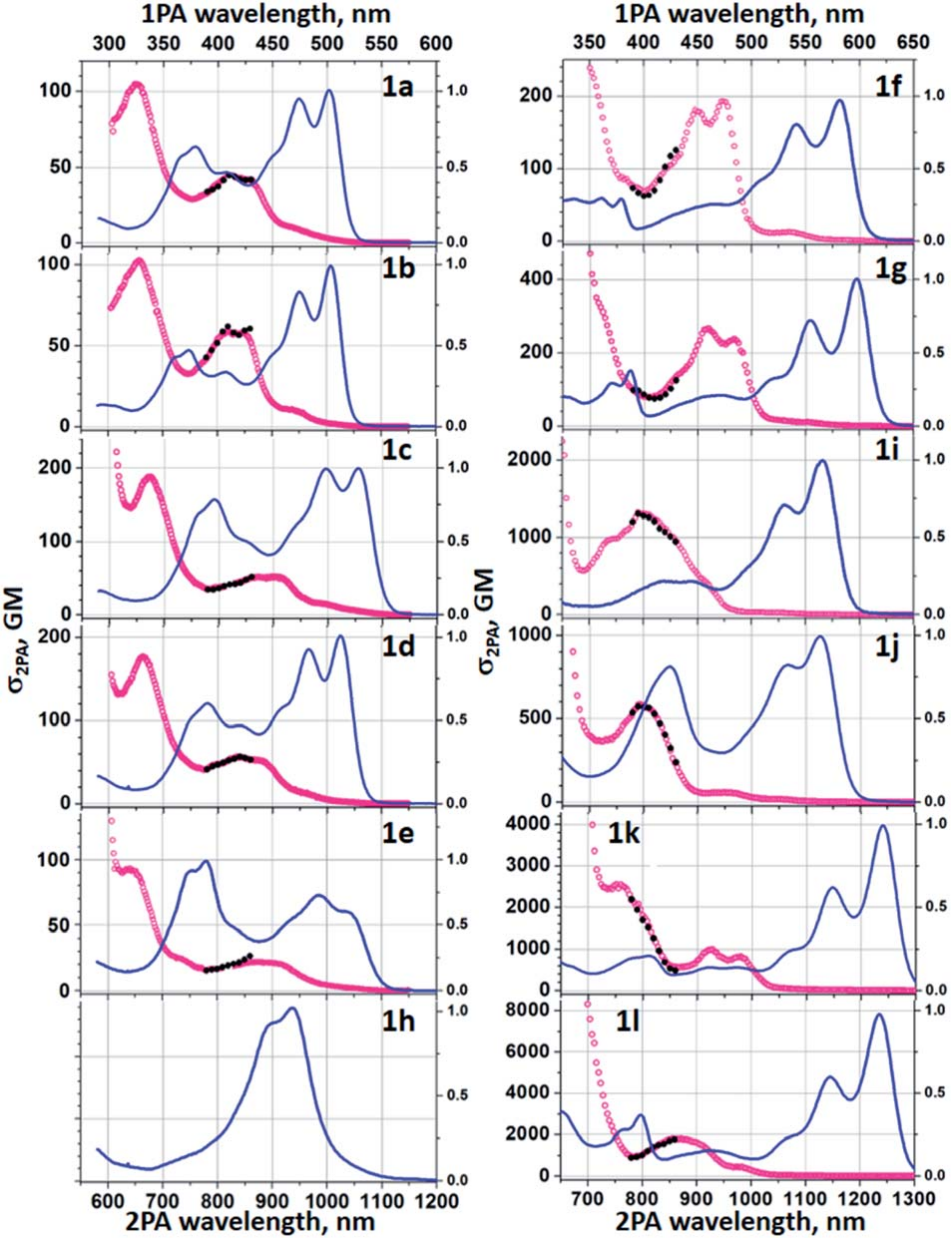
2PA cross section spectra of 1a–1l in toluene (red symbols, lower horizontal and left vertical axis); Normalized to the peak linear absorption spectra (blue line, upper horizontal and right vertical axis) is shown for comparison. Absolute *σ*_2PA_ values were measured in the range *λ*_2PA_ = 780–850 nm (black symbols).

Two-photon absorption studies were performed using a two-photon excited fluorescence (2PEF) method (Table 1 and Fig. 5). Similarly to previously studied symmetric TAPPs,^40,43*b*,*c*^ the most prominent two-photon transitions are observed at higher energies, well above the lowest-energy one-photon allowed *S*_0_→*S*_1_ transition. The latter is only very weakly present in the two-photon spectra, thus following the Laporte rule for low-energy bands. At energies above the *S*_0_→*S*_1_ transition, the 2PA profiles of 1a-e show similar features comprising a double-band or vibronic progression at 800–950 nm and a distinct peak at *ca.* 650–670 nm. At <600 nm there is a steep increase of the cross section due to the near-resonance enhancement effect, followed by onset of linear absorption at even shorter wavelengths (not shown). When pyridine is replaced with quinoline (1f, 1g) and further with the addition of diarylamino groups, these spectral features undergo a gradual bathochromic shift. Two-photon absorption was not investigated in the case of dye 1h for two main reasons: (1) the fluorescence intensity is too weak for 2PEF method to be used; (2) the perplexing photophysics of this BN-TAPP has to be delineated before intrinsically more complex non-linear phenomena will be studied. The substantial change, however, comes only after adding strong electron-withdrawing groups at the periphery (dyes 1j and 1k).

In such case the peak *σ*_2_ becomes remarkably large, exceeding 10^3^ GM. In the case of the dyes 1k and 1l, the peak values at 760–870 nm reach *σ*_2_ = 2500 GM and 1800 GM, respectively, whereas strongly resonance-enhanced values at higher energy become even larger. These values are significantly higher than for simpler A–D–A type TAPPs.^43*b*,*c*^ An important feature to note is that the values are higher for the case with the peripheral diaryl-amino groups which would suggest that the new heterocyclic core is somewhat electron-withdrawing.

### First-principle studies

Time-Dependent Density Functional Theory (TD-DFT) has been used to explore the nature of the excited-states involved in the BN-TAPPs 1a–1k. The technical details are given in the ESI.†Table 2 summarizes the main results obtained by the calculations.

**Table 2:** Computed vertical absorption wavelengths (in nm) for the lowest singlet states of all compounds with the corresponding oscillator strengths and 2PA cross-sections (in GM). For the lowest state, we also report the vertical emission wavelength and the computed 0–0 wavelength. See the experimental section for details

Compound	Vert. *λ*_abs_	*f*	*σ*_2PA_	Vert. *λ*_em_	*λ*_0–0_
1a	422	0.86	0	493	463
	352	0.00	94		
	351	0.42	0		
	322	0.26	0		
1b	424	0.88	0	496	465
	351	0.38	0		
	348	0.00	119		
	314	0.23	0		
1c	439	1.03	0	514	472
	367	0.00	122		
	362	0.43	0		
	336	0.34	0		
1d	430	0.86	0	502	460
	362	0.42	0		
	358	0.00	118		
	330	0.30	0		
1e	427	0.71	0	500	472
	372	0.00	74		
	351	0.41	0		
	335	0.41	0		
1f	479	1.14	0	560	n.d.
	384	0.00	418		
	379	0.39	0		
	317	0.29	3		
1g	494	1.17	0	572	534
	395	0.00	428		
	394	0.43	0		
	326	0.32	4		
1h	424	1.04	0	541^a^	484
	418	0.00	6		
	417	0.77	0		
1i	489	2.33	0	572	524
	386	0.52	0		
	373	0.01	1790		
	343	0.353	1		
1j	469	1.43	0	552	505
	410	0.00	82		
	378	0.57	0		
	346	0.50	0		
	334	0.00	1760		
1k	518	2.11	0	599	558
	405	0.57	0		
	400	0.00	1600		
	340	0.92	5		
1l	516	2.26	0	597	560
	406	0.01	381		
	395	0.61	1		
	358	0.00	10 900		

a Nearly dark state after optimization.

Let us start our analysis by the one-photon absorption. In the full series (except for 1h), the *S*_0_→*S*_1_ transition is well separated from the next one, and is strongly dipole allowed with oscillator strengths ranging from 0.71 to 2.33. The *S*_0_→*S*_1_ transition undoubtedly corresponds to the observed most red-shifted absorption band. Experimentally, this absorption band is typically composed of two peaks and a shoulder, resulting from vibronic couplings. To ascertain this statement, we have performed vibrationally resolved calculations within the FC-AH approach^57^ for a simplified version of dye 1f (see Fig. S4†). As can be seen, the overall topology of both the absorption and emission bands is reproduced confirming the vibronic nature of these two peaks. For 1h, the three lowest excited-states are very close according to theory (Table 1), with differences below the TD-DFT accuracy, so that it is not possible to have a definitive information regarding their ordering.

While one cannot compare directly computed vertical absorption wavelengths to experimental *λ*^max^_abs_, it is also obvious that the trends in the series are reproduced by calculations. Indeed, taking 1a as reference, one notices that the strongest red-shift is obtained for 1c (theory: +17 nm, experiment: +26 nm) in the 1a–1e series. For the six dyes showing the most red-shifted absorption, the experimental order is 1j ∼ 1i < 1f < 1g < 1l ∼ 1k, whereas theory yields: 1j < 1f < 1i ∼ 1g < 1l ∼ 1k.

**Fig. 6:**
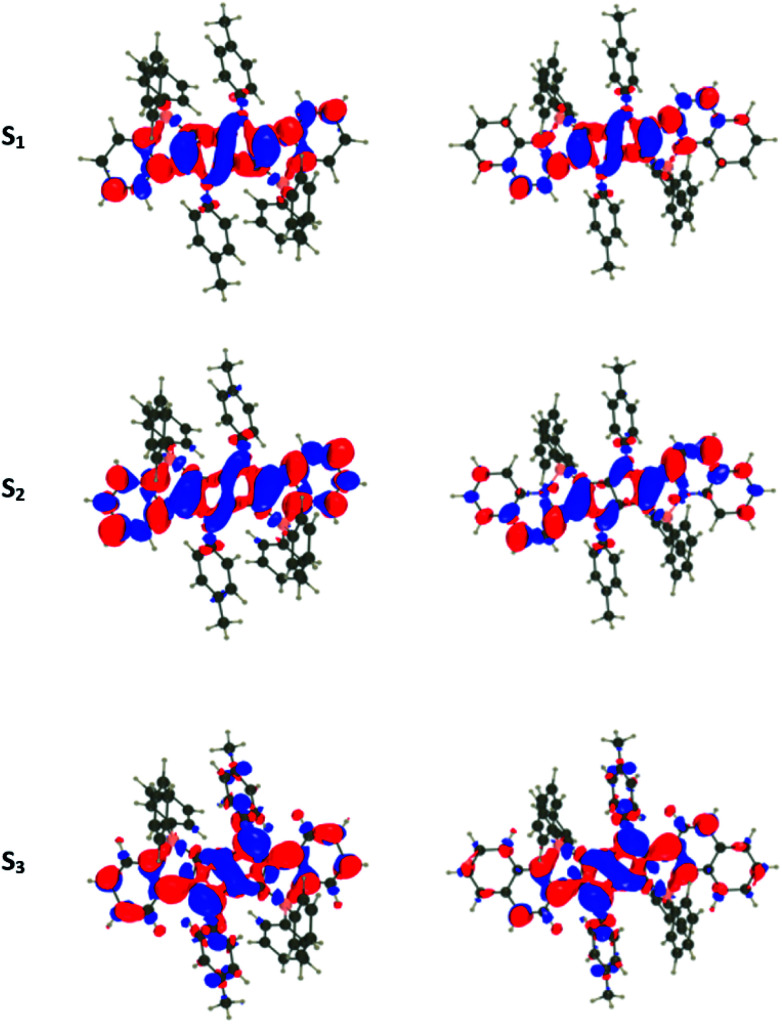
Density difference plots for the three lowest transitions of 1a (left) and 1f (right). From top to bottom excitation to S_1_, S_2_, and S_3_. The crimson and blueberry lobes indicate an increase and decrease of density upon excitation, respectively. Contour threshold: 0.001 au.

**Fig. 7:**
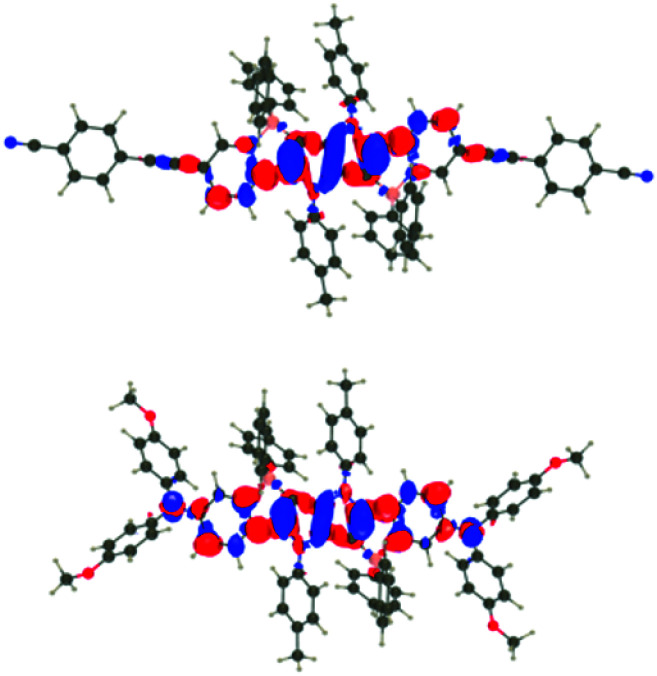
Density difference plots for the lowest transitions of 1i (top) and 1j (bottom). See caption of Fig. 6 for more details.

For all investigated dyes but 1j, the second and third excited states are very close on the energy scale, but possess vastly different oscillator strengths, which is a consequence of the nearly centro-symmetric nature of the investigated dyes. This second (or third) state corresponds to the second weaker absorption band found in the 380–420 nm region experimentally (Tables 1 and 2). For dye 1a, we show density difference plots in Fig. 6. For all three states the central pyrrolopyrrole unit acts as a donor group and the side pyridine rings are accepting moieties. The quadrupolar CT character is, however, not strongly marked for the lowest transition and becomes significantly stronger in the two higher ones. As can be seen, the nature of the state is conserved when going to 1f (derived from quinoline), the contribution of the additional benzene rings being trifling for the first state and remaining limited in the two higher states. In dye 1i, the lowest excited state is partially delocalized on the ethynyl bridges, whereas in 1j, the electron-donating amino groups play their expected role (Fig. 7) explaining the observed redshifts.

As far as fluorescence is concerned, for only one dye of the series, namely, 1h, the optimization of the excited-state geometry led to a nearly dark state (*f* = 0.009), suggesting a very slow radiative decay rate. This is consistent with the trifling emission quantum yield measured experimentally (see Table 1). Indeed the computational studies revealed that the lowest energy allowed transition is markedly blue-shifted placing is energetically close to the dark state. The fact that this pair of opposite-parity electronic states may become almost isoenergetic corroborates the anomalous photophysical behavior of dye 1h (*vide infra*). Apart from this specific case, one globally finds similar substituent effects as for absorption. In Table 2, we also report the 0–0 wavelengths that offer more physically well-grounded comparisons with experiment,^57*b*^ and more precisely, with the crossing point between absorption and emission. Illustratively, for 1a, 1j and 1l, we compute *λ*_0–0_ of 463, 505, and 560 nm, respectively. These values are blue-shifted by +0.26, 0.31, and 0.24 eV as compared to the measured values of 512, 577, and 629 nm. These errors are on the upper side of the typical TD-DFT errors, which can be explained by the very specific nature of these boron-containing systems: TD-DFT is known to overshoot the transition energies of such states.^58^

We have also computed two-photon absorption cross sections. In 1a, the experimental rather broad response around 800–900 nm is due to the *S*_0_→*S*_2_ transition that is dark in one-photon, a typical outcome in quadrupolar dyes. Theory provides a peak *σ*_2PA_ of 94 GM, of the same order of magnitude as the experimental response (45 GM). This response remains of the same order of magnitude but is slightly increased in 1b, 1c, and 1d, but slightly decreased in 1e, which holds both in the experiment and in the simulations. In 1f, the *σ*_2PA_ attains the respectable value of *ca.* 220 GM experimentally, and it is clear that it is again the second state that is responsible for this response (418 GM theoretically). The situation is totally similar in 1g. In the more extended 1i, the calculations indicate that the 2PA response comes from the *S*_0_→*S*_3_ transition, which is consistent with the experimental spectra. Again, theory gives value that closely matches the experiment (1500 GM *versus* 1400 GM). In 1i, the experimental 2PA spectrum shows two bands, one moderately active due to the second transition, and one much more intense due to the fifth state. In 1k, the second and third states appear at almost the same energy but have different (pseudo) symmetry, one being active in one-photon, the other in two-photon absorption, potentially explaining the shape of the experimental spectra resembling a superposition between the two phenomena. The computed response is 1600 GM for the 2PA-active transition, again with the same order of magnitude as the experimental measurement. Eventually, the analysis is more difficult in 1l, but it appears that the second excited state should be responsible for the shoulder in the 2PA experimental spectrum at *ca.* 475 nm, whereas the fourth transition likely yields the much more intense peaks at *ca.* 440 nm.

### Electrochemical properties

**Fig. 8:**
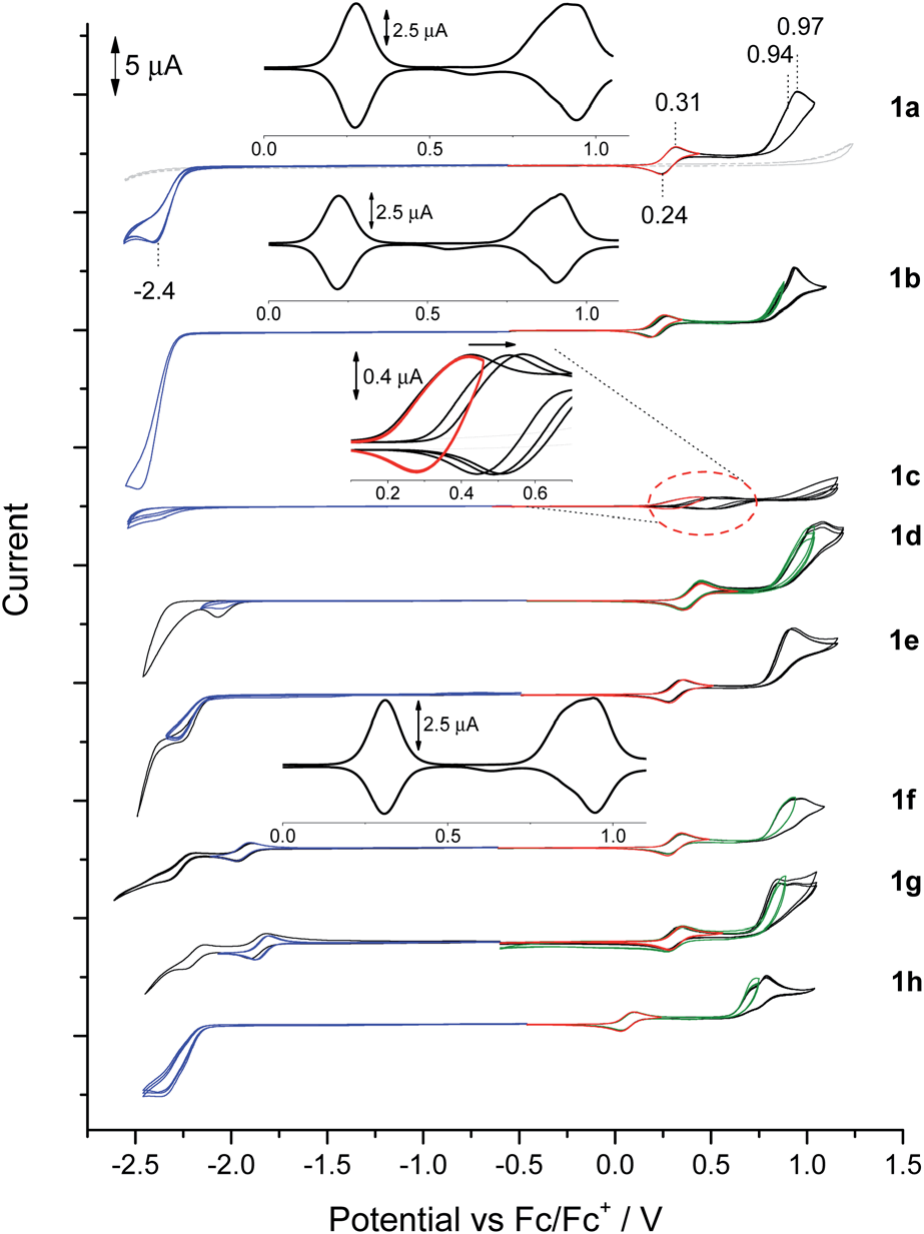
Cyclic voltammograms in the solution of BN-TAPP-series compounds (1 mM) in 0.1 M Bu_4_NBF_4_/DCM. Oxidation (red, black CVs), reduction (blue, black CVs), second oxidation (green, black CVs).

BN-TAPPs 1a–1l are characterized by the presence of a reversible redox pair of the first oxidation process (Fig. 8, red CVs, Table 3) which according to its potential value can be tentatively attributed to the oxidation of the *N*,*N*′-diphenylpyrrolo[3,2-*b*]pyrrole core. An exception is 1c where partial reversibility was found, the cause of which was further investigated during spectroelectrochemical measurements (*vide infra*). The lowest value of the first oxidation potential in the entire BN-TAPP series (0.11 V) was recorded for the derivative 1h with isoquinoline scaffolds. For the remaining dyes the first oxidation occurs between 0.25 and 0.45 V which corresponds to HOMO values in the range (−5.26 eV)–(−5.43 eV) (Table 3). Compared to structurally analogous dyes possessing two thiophene rings BN-TAPPs are slightly less electron-rich but they are more electron-rich than dyes π-expanded TAPPs possessing two thiophene-*S*,*S*-dioxide moieties.^52*b*^

**Table 3:** Potential of cathodic peak (cp) and anodic peak (ap) of oxidation (ox) and reduction (red) processes of BN-TAPPs (1 mM) in 0.1 M Bu_4_NBF_4_/DCM; values in volts *vs.* Fc/Fc^+^ couple; potential sweep rate 0.05 V s^−1^

Compound	*E* ^1^_ox_		*E* ^2a^_ox_	*E* ^2b^_ox_	*E* ^1^_red_		*E* ^2^_red_	HOMO (eV)	LUMO (eV)
	*E* ^1^_ap_	*E* ^1^_cp_	*E* ^2a^_ap_	*E* ^2b^_ap_	*E* ^1^_cp_	*E* ^1^_ap_	*E* ^2^_cp_		
1a	0.31	0.24	0.94	0.97	−2.40	—	—	−5.30	−2.84
1b	0.25	0.19	0.87	0.93	−2.48	—	—	−5.26	−2.81
1c	0.42	0.28	—	—	−2.38	—	—	−5.31	−2.81
1d	0.45	0.36	1.02	1.08	−2.04	—	—	−5.43	−3.13
1e	0.34	0.26	0.90	—	−2.30	—	—	−5.34	−2.98
1f	0.35	0.28	0.93	0.97	−1.97	−1.90	−2.32	−5.35	−3.24
1g	0.41	0.34	0.92	1.01	−1.78	−0.68	−1.11	−5.35	−3.32
1h	0.11	0.05	0.74	0.81	−2.46	—	—	−5.12	−2.96

The second oxidation curve is partially irreversible in all cases (Fig. 8, green and black CVs) and shows a dual nature more visible in differential pulse voltammetry (DPV) experiments (Fig. 8, *e.g.*1a and 1b – inserts). The double peak of the second oxidation state may indicate a slight difference in oxidation potential between both peripheral units, which was also registered in separate processes as green and black CV curves with the peak potential marked as *E*^2a^_ox_ and *E*^2b^_ox_, respectively. Dyes 1h and 1d possess, respectively, the lowest (0.74 V) and highest (1.02 V) potential of *E*^2a^_ox_. Irreversibility of the second oxidation does not impact on the potential and reversibility of the first redox couple in subsequent CV cycles after polarity reversal.

DPV measurements also show that the peak areas of the first and second oxidation peaks are comparable, which indicates the full oxidation of the *N*,*N*′-diphenylpyrrolo[3,2-*b*]pyrrole scaffold under the potential of the first oxidation peak to dication or di(radical cation), while the second oxidation peak is associated with the oxidation of both the peripheral moieties.

One irreversible reduction process was registered for BN-TAPPs 1a–1e bearing two pyridine moieties at (−2.40)–(−2.04 V). In the case of dye 1f (bearing two quinoline moieties), we observed two reduction processes, and the first was fully reversible. The values of reduction were shifted towards positive potentials, compared with the pyridine series.

### Spectroelectrochemistry

**Fig. 9:**
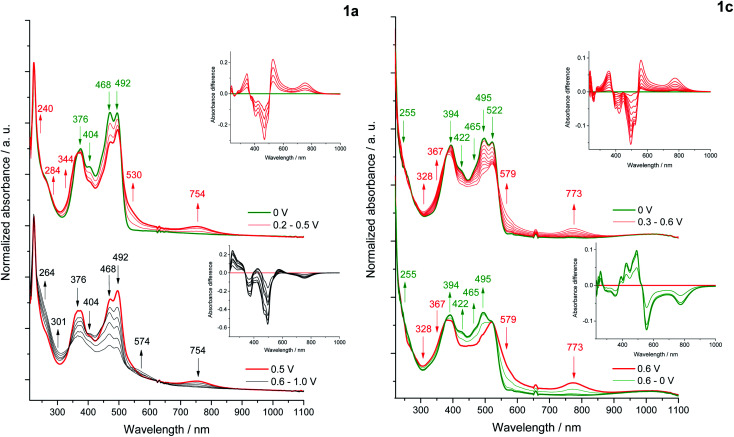
*In situ* UV-Vis-NIR spectra of 1a (left) and 1c (right); differential UV-Vis-NIR absorbance (insets) in 1 mM sample solution in 0.1 M Bu_4_NBF_4_/DCM within the potential of the first (red) (**1a**, **1c**) and second (black) oxidation peaks (1a); at 0 V or the returning to 0 V (green).

Changes in the UV-Vis-NIR absorption spectra during polarization within the first oxidation peak revealed a decrease in the intensity of the bands at 468, 492 nm (1a) and 495 and 522 nm (1c)^59^ (Fig. 9), while the shift of the absorption in the infrared direction of the output bands of 1c indicates a decrease in the energy of the π–π* transition due to a partial localization of the HOMO orbital also on the pyridine rings. Polarization within the first oxidation peak causes an increase in the bands at 530, 734 nm (1a) and 579, 773 nm (1c), characteristic for a di(radical cationic) state, as was observed in a previous spectroelectrochemical study of dyes based on the pyrrolo[3,2-*b*]pyrrole scaffold.^52*b*^ The gradual formation of a radical cation and its transition to di(radical cation) was not observed.

ESR spectroelectrochemical measurements were performed for representative dyes 1a and 1c, where the reversible first redox process is associated with the formation of a di(radical cation) in both cases. Unpaired electrons of the di(radical cation) of 1a are located on the *N*,*N*′-diphenylpyrrolo[3,2-*b*]pyrrole core and are characterized by a low value of the *g*-factor (*g* = 2.0023). In turn, the ESR signal recorded under polarization of the first oxidation state of 1c gives the *g*-factor equal 2.0050, which may indicate the coupling of one of the electrons of the di(radical cation) with nitrogen and/or boron atoms, as proposed in Scheme S1.† Although the ESR signals are quite broad in both cases, which may indicate the cleavage of the energy levels of the unpaired electron by the nuclei coupled to it, the hyperfine structure was not recorded.

## Conclusions

In summary, we have designed, synthesized and fully characterized a new family of BN-embedded heteroacenes. This was achieved through incorporation of duel cyclopenta[*c*][1,2]azaborole moieties with a pyrrolo[3,2-*b*]pyrrole core in a straightforward two-step synthetic procedure. The B^−^–N^+^ dative bond reduces the HOMO–LUMO gap of the parent dye, which results in a marked red-shift of absorption and emission and almost quantitative fluorescence quantum yield. Their excellent properties, such as superb photostability, strong absorption and intense emission in the orange to deep red region, together with large two-photon absorption cross sections and rich electrochemistry, opens the door for future applications in optoelectronics. Given the recent renaissance of interest in boron-doped PAHs and their related B–N/O isosteres, this work should inspire the future design and synthesis of pyrrolopyrrole and related frameworks with distinctive π-expanded architectures.

## Data availability

Data associated with this article, including experimental procedures, compound characterization, steady-state absorption and emission along with the two-photon absorption details, electrochemical details and computational analysis details are available in the ESI.†

## Author contributions

M. T. conceived the idea and wrote the manuscript. M. T., P. K and M. P. performed all synthetic experiments including condition optimizations and exploring the scope. M. C. and P. J. performed electrochemical and spectroelectrochemical measurements. M. B. performed several TD-DFT calculations, and vibronic analysis. M. R. performed 2PA measurements. M. Ł. wrote formal electrochemical analysis and reviewed the manuscript. A. R. performed 2PA measurements, wrote formal analysis of this part of the manuscript and reviewed the manuscript. D. J. performed DFT and TD-DFT calculations, analyzed data, wrote and reviewed the manuscript. D. G. supervised the project, performed formal analysis, wrote and reviewed the manuscript. All the authors discussed the results and commented on the manuscript.

## Conflicts of interest

There are no conflicts to declare.

## Supplementary Material

SC-012-D1SC05007A-s001

SC-012-D1SC05007A-s002
