# Supplementary material for: Going beyond the borders: pyrrolo[3,2-b]pyrroles with deep red emission
Source: Chem Sci. 2021 Nov 22;12(48):15935–46. doi: 10.1039/d1sc05007a (PMC8672719; doi:10.1039/d1sc05007a)
Supplement: SC-012-D1SC05007A-s001 [file SC-012-D1SC05007A-s001.pdf]

Electronic Supplementary Material (ESI) for Chemical Science.
This journal is © The Royal Society of Chemistry 2021

Supporting information for

Going beyond the borders: pyrrolo[3,2-b]pyrroles with deep red emission

Mariusz Tasior,[a] Paweł Kowalczyk,[a] Marta Przybył,[a] Małgorzata Czichy,[b] Patryk Janasik,[b] Manon H. E. Bousquet,[c] Mieczysław Łapkowski,*[b,d] Matt Rammo,[e] Aleksander Rebane,*[e,f] Denis Jacquemin*[c] and Daniel T. Gryko*[a]

[a] Institute of Organic Chemistry, Polish Academy of Sciences, Kasprzaka 44–52, 01-224 Warsaw, Poland.

[b] Faculty of Chemistry, Silesian University of Technology, Strzody 9, 44-100 Gliwice, Poland.

[c] CEISAM Lab—UMR 6230, CNRS, University of Nantes, Nantes, France. E-mail : Denis.Jacquemin@univ-nantes.fr

[d] Centre of Polymer and Carbon Materials, Polish Academy of Sciences, Curie-Sklodowskiej 34, 41-819 Zabrze, Poland.

[e] National Institute of Chemical Physics and Biophysics, Tallinn, Estonia.

[f] Department of Physics, Montana State University, Bozeman, MT, 59717, USA.

Table of contents

1. **General information** 1-2
2. **Synthesis** 3-7
3. **Crystallographic data** 8-10
4. **Spectroscopic properties** 11
5. **Absorption and emission spectra** 12-14
6. **Electrochemical properties** 15-16
7. **Calculations** 17
8. **^{1}H, ^{13}C, ^{19}F and ^{10}B NMR spectra** 18-44

1. General information

All chemicals were used as received, unless otherwise noted. All reported NMR spectra were recorded on a 500 MHz spectrometer unless otherwise noted. Chemical shifts (δ; ppm) for ^{1}H and ^{13}C NMR were determined with TMS as the internal reference. *J* values are given in Hz. The absorption and fluorescence spectra of **2a-h** and **1a-l** were recorded in toluene. Mass spectra were obtained with EI ion source and the EBE double focusing geometry mass analyzer or spectrometer equipped with electro-spray ion source with Q-TOF type mass analyzer.

Electrochemical methods used were cyclic voltammetry and differential pulse voltammetry using CH Instruments Electrochemical Analyzer model 620 with the parameters for the first and second techniques, respectively: potential sweep rate of 0.05 V/s; step size of 4 mV, pulse size of 25 mV and frequency of 15 Hz. Setup was in the single-cell with 2 mm^2 - platinum disk (working electrode), platinum coil (counter electrode) and Ag|Ag$^+$ pseudo-reference electrode calibrated with ferrocene|ferrocenium Fc|Fc$^+$ couple as the internal standard. UV-Vis-NIR spectroelectrochemical measurement was performed using Agilent/HP 8453 UV-Visible Spectrophotometer G1103A. A thin-layer optical cell characterized by the optical path of 0.075 mm length was a quartz cuvette containing ITO/quartz electrode (20±5 V/sq, Praezisions Glas & Optic GmbH), Ag|Ag$^+$ pseudo-reference electrode and platinum mesh as a counter electrode. Electron paramagnetic resonance (ESR) spectroelectrochemical measurement was performed using JEOL JES FA-200 X-band spectrometer with the following parameters: 1 mT (modulation width); 1 mW (microwave power); 50 (amplitude) in a system of electrodes: platinum wire (working electrode), platinum coil (counter electrode) and Ag|Ag$^+$ pseudo-reference electrode. Sample concentration was 1.0 mM in dichloromethane or acetonitrile in the presence of 0.1 M Bu_4NBF_4 electrolyte. The solution was deoxygenated by flushing with argon before all electrochemical and spectroelectrochemical measurements.

Tetrabutylammonium tetrafluoroborate (Bu_4NBF_4, 99.0%, Sigma-Aldrich) dried under vacuum, dichloromethane (DCM, for HPLC, 99.8%, Sigma-Aldrich), ferrocene (Fc, 98%, Sigma-Aldrich) and argon (6.0, SIAD Group) were used for the above mentioned studies.

The optical properties have been modelled with DFT and TD-DFT. We used exact experimental structures, but for the side alkyl chains that were replaced by methyl groups for obvious computational reasons. The ground-state geometries have been fully optimized with Gaussian 16[1] using Pople's 6-31G(d) basis set, Truhlar's M06-2X[2] hybrid exchange-correlation functional, and using the PCM[3] model for the environmental effects (toluene). Default or improved convergence parameters have been used. Next, frequency calculations have been systematically achieved - at the very same level of theory - to confirm the minimum nature of the optimized geometries. Starting from these ground-state structures, TD-DFT optimizations and frequency calculations of the lowest singlet states have been performed as well, using the same setting. For the one-photon (vertical) absorption and emission, we next used linear-response TD-DFT, with the same functional, but a diffuse-containing atomic basis set, namely 6-31+G(d,p). The selected solvent model was LR-PCM[4] which is justified as the lowest transition is strongly dipole allowed. We note that in toluene the *equilibrium* and *non-equilibrium* limits of PCM-TD-DFT calculations are essentially equivalent. From these calculations, we deduced 0-0 energies directly, by correcting the adiabatic energies for the differences of ZPVE between the two states.[5] Advantageously, 0-0 energies can be directly

compared to the experimentally-available absorption-fluorescence crossing point. For one case, we performed vibrationally resolved calculations of both one-photon absorption and emission. The vibronic spectra have been simulated using the FCclasses3[6] (*Beta* version) code within the time-independent formulation, applying the Franck-Condon model, and using the Adiabatic Hessian (AH) model at 298K. Both properties have been computed with 10^7 integrals and the emission have been broadened using a Lorentzian function (HWHM = 0.03 eV) while using a Gaussian function (HWHM = 0.045 eV) for the absorption. These calculations recovered very large fractions of the FC spectrum (>0.97) indicating a well-converged calculation. Finally, the two-photon cross-sections were simulated using the quadratic response model implemented in Dalton[7] using the optimal geometries. These two-photon calculations were performed with CAM-B3LYP[8] combined to the 6-31G basis set. As a final note, we are well aware of the limits of TD-DFT for boron-containing complexes like "BODIPY" and similar, [9] but the size of the investigated compounds clearly prevents the use of Coupled-Cluster (or similar) schemes.

2. Synthesis

General procedure for the synthesis of pyrrolo[3,2-b]pyrroles

Glacial acetic acid (6 mL), toluene (6 mL), aldehyde (8 mmol) and aniline (8 mmol) were placed in a 50 mL round-bottom flask equipped with a magnetic stir bar. The mixture was stirred at 90 °C for 30 min. After that time, $Fe(ClO4)_3$ (92 mg) was added, followed by diacetyl (350 µL, 4 mmol). The resulting mixture was stirred at 50 °C for 16 h. Solvents were evaporated off. Residue was dissolved in hot ACN and cooled to rt, then in the fridge overnight. The resulting precipitate was filtered off, washed with cold ACN and dried under high vacuum.

1,4-Bis(4-octylphenyl)-2,5-bis(pyrid-2-yl)-1,4-dihydropyrrolo[3,2-b]pyrrole (2a)

Brown solid. Yield: 535 mg (21%). Spectroscopic properties were in agreement with the literature data.[10]

2,5-Bis(6-methylpyrid-2-yl)-1,4-bis(4-octylphenyl)-1,4-dihydropyrrolo[3,2-b]pyrrole (2b)

Brown solid. Yield: 287 mg (11%). ^{1}H NMR (500 MHz, $CDCl_3$) δ 7.28 (t, *J* = 7.8 Hz, 2H), 7.22 (d, *J* = 8.2 Hz, 4H), 7.16 (d, *J* = 8.2 Hz, 4H), 6.85 (d, *J* = 7.6 Hz, 2H), 6.76 (s, 2H), 6.75 (d, *J* = 7.5 Hz, 2H), 2.62 (t, *J* = 7.7 Hz, 4H), 2.46 (s, 6H), 1.64 (q, *J* = 7.3 Hz, 4H), 1.42 - 1.19 (m, 20H), 0.88 ppm (t, *J* = 6.8 Hz, 6H); ^{13}C NMR (126 MHz, $CDCl_3$) δ 157.9, 151.3, 140.6, 138.1, 136.5, 135.6, 133.4, 128.9, 125.4, 119.8, 119.3, 96.4, 35.5, 31.9, 31.4, 29.5, 29.3, 29.2, 24.6, 22.7, 14.1 ppm. HRMS (EI): *m/z* calculated for $C_{46}H_{56}N_4$: 664.4505 [$M^{\cdot+}$]; found: 664.4498

2,5-Bis(5-bromopyrid-2-yl)-1,4-bis(4-octylphenyl)-1,4-dihydropyrrolo[3,2-b]pyrrole (2c)

Brown solid. Yield: 1340 mg (42%). ^{1}H NMR (500 MHz, $CDCl_3$) δ 8.54 (s, 2H), 7.53 (d, *J*= 8.5 Hz, 2H), 7.24 - 7.16 (m, 8H), 6.83 (d, *J* = 8.6 Hz, 2H), 6.81 (s, 2H), 2.64 (t, *J*= 7.5 Hz, 4H), 1.65 (quintet, *J* =7.3 Hz, 4H), 1.39 - 1.23 (m, 20H), 0.89 ppm (t, *J* = 6.8 Hz, 6H); ^{13}C NMR (126 MHz, $CDCl_3$) δ 149.9, 149.8, 141.5, 138.5, 137.3, 135.5, 134.1, 129.3, 125.3, 123.1, 117.0, 97.2, 35.5, 31.9, 31.4, 29.5, 29.3, 22.7, 14.1 ppm. HRMS (EI): *m/z* calculated for $C_{44}H_{50}N_4Br_2$: 792.2402 [$M^{\cdot+}$]; found: 792.2386.

2,5-Bis(4-bromopyrid-2-yl)-1,4-bis(4-octylphenyl)-1,4-dihydropyrrolo[3,2-b]pyrrole (2d)

Yellow solid. Yield: 932 mg (29%). ^{1}H NMR (500 MHz, $CDCl_3$) δ 8.27 (dd, *J* = 4.9, 1.1 Hz, 2H), 7.22 (s, 8H), 7.17 – 7.12 (m, 4H), 6.78 (s, 2H), 2.65 (t, *J* = 7.6 Hz, 4H), 1.65 (quintet, *J* = 7.2 Hz,

4H), 1.35 - 1.26 (m, 20H), 0.89 ppm (t, *J* = 6.7 Hz, 6H); ^{13}C NMR (126 MHz, $CDCl_3$) δ 153.0, 150.0, 141.3, 137.4, 135.4, 133.9, 132.1, 129.2, 125.3, 124.9, 123.5, 97.1, 35.5, 31.9, 31.4, 29.5, 29.3, 29.1, 22.7, 14.1 ppm. HRMS (EI): *m/z* calculated for $C_{44}H_{50}N_4Br_2$: 792.2402 [$M^{\cdot+}$]; found: 792.2388.

2,5-Bis(5-fluoropyrid-2-yl)-1,4-bis(4-octylphenyl)-1,4-dihydropyrrolo[3,2-b]pyrrole (2e)

Brown solid. Yield: 800 mg (30%). ^{1}H NMR (500 MHz, $CDCl_3$) δ 8.36 (d, *J* = 2.9 Hz, 2H), 7.24 - 7.11 (m, 10H), 6.97 (dd, *J* = 8.9, 4.3 Hz, 2H), 6.70 (s, 2H), 2.63 (t, *J* = 7.7 Hz, 4H), 1.64 (quintet, *J* = 7.1 Hz, 4H), 1.38 - 1.24 (m, 20H), 0.89 ppm (t, *J* = 6.8 Hz, 6H); ^{13}C NMR (126 MHz, $CDCl_3$) δ 157.5 (d, *J* = 255.2 Hz), 148.3 (d, *J* = 3.8 Hz), 141.0, 137.6, 137.4, 135.3, 133.1, 129.1, 125.2, 122.9 (d, *J* = 3.8 Hz), 122.5 (d, *J* = 18.8 Hz), 96.4, 35.5, 31.9, 31.4, 29.5, 29.3, 22.7, 14.1 ppm; ^{19}F NMR (470 MHz, $CDCl_3$) δ -130.92 ppm (dd, *J* = 8.4, 4.6 Hz). HRMS (EI): *m/z* calculated for $C_{44}H_{50}N_4F_2$: 672.4004 [$M^{\cdot+}$]; found: 672.4001.

1,4-Bis(4-octylphenyl)-2,5-bis(quinolin-2-yl)-1,4-dihydropyrrolo[3,2-b]pyrrole (2f)

Brown solid. Yield: 800 mg (28%). ^{1}H NMR (600 MHz, $CDCl_3$) δ 7.92 (br s, 2H), 7.87 (d, *J* = 9.0 Hz, 2H), 7.68 (d, *J* = 7.9 Hz, 2H), 7.63 (s, 2H), 7.43 (t, *J* = 7.4 Hz, 2H), 7.30 (d, *J* = 7.9 Hz, 4H), 7.19 (d, *J* = 7.7 Hz, 6H), 6.98 (br s, 2H), 2.64 (t, *J* = 7.6 Hz, 4H), 1.64 (quintet, *J* = 7.3 Hz, 4H), 1.30 (m, 20H), 0.88 ppm (t, *J* = 6.6 Hz, 6H); ^{13}C NMR (151 MHz, $CDCl_3$) δ 129.1, 127.4, 126.3, 125.9, 125.4, 121.2, 35.5, 31.0, 31.4, 29.5, 29.28, 29.24, 22.7, 14.1. HRMS (EI): *m/z* calculated for $C_{52}H_{56}N_4$: 736.4505 [$M^{\cdot+}$]; found: 736.4495.

2,5-Bis(6-chloroquinolin-2-yl)-1,4-bis(4-octylphenyl)-1,4-dihydropyrrolo[3,2-b]pyrrole (2g)

Brown solid. Yield: 1270 mg (39%). ^{1}H NMR (500 MHz, $CDCl_3$) δ 7.78 (d, *J* = 5.1 Hz, 2H), 7.77 (d, *J* = 5.3 Hz, 2H), 7.66 (d, *J* = 2.0 Hz, 2H), 7.54 (dd, *J* = 8.9, 2.3 Hz, 2H), 7.28 (d, *J* = 8.3 Hz, 4H), 7.24 (d, *J* = 8.7 Hz, 2H), 7.20 (d, *J* = 8.6 Hz, 2H), 6.94 (s, 2H), 2.66 (t, *J* = 7.7 Hz, 4H), 1.66 (quintet, *J* = 7.5 Hz, 4H), 1.37 – 1.24 (m, 20H), 0.90 (t, *J* = 7.0 Hz, 6H); ^{13}C NMR (126 MHz, $CDCl_3$) δ 151.9, 146.5, 141.1, 137.9, 136.9, 134.3, 134.2, 131.2, 130.6, 130.3, 129,0 126.9, 126.0, 125.4, 121.7, 97.4, 35.5, 31.9, 31.4, 29.5, 29.3, 29.2, 22.7, 14.1 ppm. HRMS (EI): *m/z* calculated for $C_{52}H_{54}N_4Cl_2$: 804.3726 [$M^{\cdot+}$]; found: 804.3735.

2,5-Bis(isoquinolin-3-yl)-1,4-bis(4-octylphenyl)-1,4-dihydropyrrolo[3,2-b]pyrrole (2h)

Dark brown solid. Yield: 160 mg (7%).^{1}H NMR (500 MHz, $CDCl_3$) δ 9.18 (s, 2H), 7.88 (d, *J* = 8.2 Hz, 2H), 7.56 (t, *J* = 7.6 Hz, 2H), 7.50-7.44 (m, 4H), 7.33 (d, *J* = 8.4 Hz, 4H), 7.24 (d, overlapped with a signal of chloroform, *J* = 10.6 Hz, 2H), 7.20 (d, *J* = 8.2 Hz, 4H), 6.98 (s, 2H), 2.65 (t, *J* = 7.6 Hz, 4H), 1.65 (quintet, *J* = 7.4 Hz, 4H), 1.39 – 1.21 (m, 20H), 0.89 ppm (t, *J* = 6.9 Hz, 6H); ^{13}C NMR (126 MHz, $CDCl_3$) δ 151.7, 145.1, 141, 137.9, 136.2, 134.0, 130.6, 129.2, 129.0, 127.7, 126.6, 126.5, 125.5, 117.6, 110.0, 97.1, 35.5, 31.9, 31.4, 29.5, 29.3, 29.2, 22.7, 14.1. HRMS (EI): *m/z* calculated for $C_{52}H_{56}N_4$: 736.4505 [$M^{\cdot+}$]; found: 736.4503.

General procedure for the synthesis of BN-embedded pyrrolo[3,2-b]pyrroles (BN-TAPPs)

The Schenk flask was charged with pyrrolo[3,2-b]pyrrole (0.4 mmol) and $PhBF_3K$ (530 mg, 2.88 mmol). Solids were evacuated and refilled with argon 3 times. Then, solids were dissolved in dry toluene, followed by addition of $SiCl_4$ (92 µL, 0.8 mmol) and Hünig's base (698 µL, 4 mmol). The resulting mixture was stirred at 135 °C overnight. Reaction mixture was cooled down to rt. The formed precipitate was diluted with DCM and the whole mixture was transferred to a round-bottom flask. After adding the proper amount of silica, all solvents were evaporated

and the residue was purified by column chromatography (SiO_2, DCM/hexanes). Product was recrystallized from $CHCl_3$/MeOH.

(1a)

Yellow solid. Yield: 201 mg (52%). ^{1}H NMR (500 MHz, $CDCl_3$) δ 8.06 (d, *J* = 5.7 Hz, 2H), 7.51 (t, *J* = 7.8 Hz, 2H), 7.12 - 7.04 (m, 16H), 6.99 (d, *J* = 8.5 Hz, 4H), 6.94 (d, *J* = 8.5 Hz, 2H), 6.92 - 6.88 (m, 8H), 6.82 (t, *J* = 6.6 Hz, 2H), 2.63 (t, *J* = 7.6 Hz, 4H), 1.64 (quintet, *J* = 7.3 Hz, 4H), 1.39 - 1.25 (m, 20H), 0.90 ppm (t, *J* = 6.8 Hz, 6H); ^{13}C NMR (126 MHz, $CDCl_3$) δ 150.8, 149.6, 144.8, 141.9, 140.5, 139.4, 137.4, 136.4, 133.6, 128.9, 127.2, 126.9, 125.2, 116.9, 115.8, 35.6, 32.0, 31.6, 29.6, 29.3, 29.2, 22.7, 14.1 ppm; ^{10}B NMR (54 MHz, $CDCl_3$) δ 49.7 ppm. HRMS (ESI): *m/z* calculated for $C_{68}H_{71}B_2N_4$: 965.5865 [M+H$^+$]; found: 965.5900.

(1b)

Orange solid. Yield: 60 mg (16%). ^{1}H NMR (500 MHz, $CDCl_3$) δ 7.41 (t, *J* = 8.0 Hz, 2H), 7.11 - 6.98 (m, 16H), 6.88 (d, *J* = 7.6 Hz, 4H), 6.85 - 6.73 (m, 10H), 6.61 (d, *J* = 7.5 Hz, 2H), 2.71 (t, *J* = 7.7 Hz, 4H), 2.07 (s, 6H), 1.77 (quintet, *J* = 7.1 Hz, 4H), 1.51 - 1.28 (m, 20H), 0.90 ppm (t, *J* = 6.5 Hz, 6H); ^{13}C NMR (126 MHz, $CDCl_3$) δ 157.0, 152.1, 147.3, 141.8, 139.4, 136.6, 136.3, 133.5, 128.8, 127.6, 126.9, 124.7, 118.8, 113.3, 35.7, 32.0, 31.9, 29.6, 29.3, 29.2, 22.7, 22.5, 14.1 ppm; ^{10}B NMR (54 MHz, $CDCl_3$) δ 133.9 ppm; HRMS (ESI): *m/z* calculated for $C_{70}H_{75}B_2N_4$: 993.6178 [M+H$^+$]; found: 993.6196.

(1c)

Orange solid. Yield: 110 mg (10%). ^{1}H NMR (500 MHz, $CDCl_3$) δ 8.13 (d, *J* = 2.1 Hz, 2H), 7.58 (dd, *J* = 8.9, 2.1 Hz, 2H), 7.14 - 7.02 (m, 16H), 6.98 (d, *J* = 8.0 Hz, 4H), 6.92 - 6.85 (m, 8H), 6.79 (d, *J* = 8.9 Hz, 2H), 2.63 (t, *J* = 7.6 Hz, 4H), 1.64 (quintet, *J* = 7.3 Hz, 4H), 1.39 - 1.25 (m, 20H), 0.91 ppm (t, *J* = 6.8 Hz, 6H); ^{13}C NMR (126 MHz, $CDCl_3$) δ 149.3, 145.8, 142.32, 142.27, 140.9, 137.2, 135.9, 134.7, 133.5, 131.1, 129.0, 127.9, 127.1, 125.5, 116.6, 111.2, 35.6, 32.0, 31.6, 29.5, 29.3, 29.2, 22.7, 14.1 ppm; ^{10}B NMR (54 MHz, $CDCl_3$) δ 42.6 ppm. HRMS (ESI): *m/z* calculated for $C_{68}H_{69}B_2N_4Br_2$: 1121.4075 [M+H$^+$]; found: 1121.4098.

(1d)

Orange solid. Yield: 130 mg (29%). ^{1}H NMR (500 MHz, $CDCl_3$) δ 7.85 (d, *J* = 6.2 Hz, 2H), 7.13 - 6.99 (m, 22H), 6.96 (dd, *J* = 6.2, 1.9 Hz, 2H), 6.90 - 6.84 (m, 8H), 2.66 (t, *J* = 7.5 Hz, 4H), 1.65 (quintet, *J* = 6.8 Hz, 4H), 1.38 - 1.25 (m, 20H), 0.90 ppm (t, *J* = 6.7 Hz, 6H); ^{13}C NMR (126 MHz, $CDCl_3$) δ 151.1, 145.2, 142.5, 141.0, 136.9, 136.6, 135.7, 133.4, 129.1, 127.1, 127.1, 125.5, 120.3, 119.0, 35.5, 32.0, 31.6, 29.6, 29.3, 29.1, 22.7, 14.1 ppm; ^{10}B NMR (54 MHz, $CDCl_3$) δ 105.0 ppm. HRMS (ESI): *m/z* calculated for $C_{68}H_{69}B_2N_4Br_2$: 1121.4075 [M+H$^+$]; found: 1121.4099.

(1e)

Orange solid. Yield: 168 mg (42%). ^{1}H NMR (500 MHz, $CDCl_3$) δ 7.99 (t, *J* = 2.6 Hz, 2H), 7.37 - 7.30 (m, 2H), 7.14 - 7.04 (m, 16H), 6.99 (d, *J* = 8.2 Hz, 4H), 6.93 - 6.86 (m, 10H), 2.63 (t, *J* = 7.6 Hz, 4H), 1.64 (quintet, *J* = 7.5 Hz, 4H), 1.40 - 1.27 (m, 20H), 0.91 (t, *J* = 6.9 Hz, 6H); ^{13}C NMR (126 MHz, $CDCl_3$) δ 155.1 (d, *J* = 247.0 Hz), 148.1, 142.2, 140.3, 136.5, 136.1, 133.5, 133.2, 129.0, 127.9 (d, *J* = 19.8 Hz), 127.1, 125.5, 116.1 (d, *J* = 5.2 Hz), 35.6, 32.0, 31.6, 29.5, 29.3, 29.2, 22.7, 14.1 ppm; ^{19}F NMR (470 MHz, $CDCl_3$) δ -132.54 – -132.61 ppm (m). ^{10}B NMR (54

MHz, $CDCl_3$) δ -28.1 ppm. HRMS (ESI): *m/z* calculated for $C_{68}H_{69}B_2N_4F_2$: 1001.5676 [M+H$^+$]; found: 1001.5705.

(1f)

Maroon solid. Yield: 77 mg (18%). ^{1}H NMR (500 MHz, $CDCl_3$) δ 7.92 (d, *J* = 9.0 Hz, 2H), 7.70 (d, *J* = 8.3 Hz, 2H), 7.60 (dd, *J* = 7.6, 1.9 Hz, 2H), 7.23 - 7.17 (m, covered by a signal from $CHCl_3$, 4H), 7.15 (d, *J* = 8.9 Hz, 2H), 7.09 (d, *J* = 8.3 Hz, 4H), 7.05 - 6.93 (m, 16H), 6.79 - 6.75 (m, 8H), 2.76 (t, *J* = 7.5 Hz, 4H), 1.80 (quintet, *J* = 4H), 1.52 - 1.30 (m, 20H), 0.91 (t, *J* = 7.0 Hz, 6H) ppm. ^{13}C NMR (126 MHz, $CDCl_3$) δ 151.9, 148.7, 142.9, 142.4, 140.8, 140.2, 139.1, 136.4, 133.7, 131.1, 129.1, 128.2, 127.9, 126.9, 125.5, 124.7, 124.6, 122.9, 115.4, 35.8, 31.9, 31.8, 29.6, 29.4, 29.3, 22.7, 14.1 ppm. ^{10}B NMR (54 MHz, $CDCl_3$) δ 39.4 ppm. HRMS (ESI): *m/z* calculated for $C_{76}H_{75}B_2N_4$: 1065.6178 [M+H$^+$]; found: 1065.6211.

(1g)

Dark red solid. Yield: 107 mg (24%). ^{1}H NMR (500 MHz, $CDCl_3$) δ 7.84 (d, *J* = 9.1 Hz, 2H), 7.65 (d, *J* = 9.3 Hz, 2H), 7.60 (d, *J* = 2.3 Hz, 2H), 7.19 (d, *J* = 9 Hz, 2H), 7.18 (dd, *J* = 9.5, 2.5 Hz, 2H), 7.10 (d, *J* = 8.0 Hz, 4H), 7.08 - 6.98 (m, 12H), 6.96 (d, *J* = 8.3 Hz, 4H), 6.79 - 6.70 (m, 8H), 2.76 (t, *J* = 7.6 Hz, 4H), 1.80 (quintet, *J* = 7.45 Hz, 4H), 1.53 - 1.30 (m, 20H), 0.92 (t, *J* = 7 Hz, 6H) ppm. ^{13}C NMR (126 MHz, $CDCl_3$) δ 151.7, 142.6, 141.3, 141, 139.3, 139.2, 136.2, 133.6, 131.8, 130.4, 129.2, 127.8, 127, 126.8, 126.2, 125, 124.3, 116.5, 35.8, 31.95, 31.9, 29.6, 29.3, 29.25, 22.7, 14.1 ppm. ^{10}B NMR (54 MHz, $CDCl_3$) δ -30.8 ppm. HRMS (ESI): *m/z* calculated for $C_{76}H_{73}B_2N_4Cl_2$: 1133.5398 [M+H$^+$]; found: 1133.5417.

(1h)

Orange solid. Yield: 16 mg (8%). ^{1}H NMR (500 MHz, $CDCl_3$) δ 8.75 (s, 2H), 7.67 (d, *J* = 8.0 Hz, 2H), 7.54 (t, *J* = 7.5 Hz, 2H), 7.45 (d, *J* = 8.4 Hz, 2H), 7.30 (t, *J* = 7.4 Hz, 2H), 7.18 (d, *J* = 8.0 Hz, 4H), 7.12 - 7.05 (m, 14H), 7.02 (d, *J* = 8.0 Hz, 4H), 6.98 (d, *J* = 6.1 Hz, 8H), 2.69 (t, *J* = 7.5 Hz, 4H), 1.70 (quintet, *J* = 6.4 Hz, 4H), 1.36 (m, 20H), 0.92 (t, *J* = 6.4 Hz, 6H) ppm. ^{13}C NMR (126 MHz, $CDCl_3$) δ 150.3, 149.0, 144.9, 141.7, 140.2, 138.3, 136.7, 136.6, 133.6, 133.1, 129.2, 128.8, 127.3, 127.0, 126.5, 125.9, 125.2, 124.2, 109.8, 35.6, 32.0, 31.7, 29.6, 29.4, 29.2, 22.7, 14.2 ppm. ^{10}B NMR (54 MHz, $CDCl_3$) δ 29.43 ppm. HRMS (ESI): *m/z* calculated for $C_{76}H_{75}B_2N_4$: 1065.6178 [M+H$^+$]; found: 1065.6227.

Synthesis of compound 1i

The Schlenk flask was charged with **3c** (74 mg, 0.065 mmol), 4-ethynylbenzonitrile (24 mg, 0.19 mmol), Cs_2CO_3 (69 mg, 0,21 mmol), palladium (II) acetate (1.3 mg, 6.6 µmol) and triphenylphosphine (6.2 mg, 26.4 mmol). After three vacuum/argon cycles, reagents were dissolved in anhydrous DMSO (5 ml). The resulting mixture was stirred at 80 °C for 24 hours. The mixture was cooled to room temperature and evaporated. The residue was purified by column chromatography (SiO_2, DCM/hexanes, 2:3). Product was recrystallized from $CHCl_3$/MeOH.

Red solid. Yield: 75 mg (95%). ^{1}H NMR (500 MHz, $CDCl_3$) δ 8.22 (d, *J* = 1.8 Hz, 2H), 7.64 - 7.55 (m, 6H), 7.50 (d, *J* = 8.5 Hz, 4H), 7.15 - 7.05 (m, 16H), 7.03 - 6.98 (m, 4H), 6.94 - 6.87 (m, 10H), 2.65 (t, *J* = 7.6 Hz, 4H), 1.66 (quintet, *J* = 7.3 Hz, 4H), 1.39 - 1.27 (m, 20H), 0.91 ppm (t, *J* = 6.8 Hz, 6H); ^{13}C NMR (126 MHz, $CDCl_3$) δ 149.3, 148.5, 147.8, 142.5, 141.64, 141.60, 138.6, 135.7, 133.6, 132.1, 131.9, 129.1, 127.12, 127.10, 127.08, 125.6, 118.3, 115.6, 112.3, 112.0, 110.0, 91.6, 89.5, 35.6, 32.0, 31.6, 29.6, 29.3, 29.2, 22.7, 14.2 ppm; ^{10}B NMR (54 MHz, $CDCl_3$) δ -53.2 ppm. HRMS (EI): *m/z* calculated for $C_{86}H_{76}B_2N_6$: 1214.6318 [M$^{\cdot+}$]; found: 1214.6331.

Synthesis of compound 1j

The Schlenk flask was charged with **3c** (56 mg, 0.05 mmol), bis(4-methoxyphenyl)amine (34.4 mg, 0.15 mmol), bis(tri-*tert*-butylphosphine)palladium(0) (1.27 mg, 2.5 µmol) and sodium *tert*-butoxide (48.1 mg, 0.5 mmol). After three vacuum/argon cycles, reagents were dissolved in anhydrous toluene (5 ml). The resulting mixture was stirred at 100 °C for 24 hours. Reaction mixture was cooled to rt and quenched with a few drops of water. All solvents were evaporated off, and the residue was purified by column chromatography (SiO_2, DCM/hexanes, 1:1). Product was recrystallized from $CHCl_3$/MeOH.

Red solid. Yield: 50 mg (70%). ^1H NMR (500 MHz, $CDCl_3$) δ 7.84 (br s, 2H), 7.15 - 6.97 (m, 18H), 6.96 - 6.82 (m, 20H), 6.78 - 6.61 (m, 10H), 3.74 (s, 12H), 2.56 (t, *J* = 7.7 Hz, 4H), 1.59 (quintet, *J* = 7Hz, 4H), 1.36 - 1.20 (m, 20H), 0.87 ppm (t, *J* = 6.8 Hz, 6H); ^{13}C NMR (126 MHz, $CDCl_3$) δ 156.2, 150.1, 141.5, 139.8, 139.2, 138.1, 136.6, 133.6, 128.7, 127.1, 126.8, 125.7, 125.0, 114.9, 55.5, 35.5, 31.9, 31.7, 29.5, 29.3, 29.2, 22.7, 14.1 ppm; ^{10}B NMR (54 MHz, $CDCl_3$) δ 40.6 ppm. HRMS (ESI): *m/z* calculated for $C_{96}H_{97}B_2N_6O_4$: 1419.7757 [M+H$^+$]; found: 1419.7809.

Synthesis of compound 1k

The Schlenk flask was charged with **2g** (52.1 mg, 45.9 mmol), 4-ethynylbenzonitrile (22.9 mg, 180 mmol), Cs_2CO_3 (78 mg, 239 mmol), XPhos (6.6 mg, 14 µmol) and bis(acetonitrile)dichloropalladium(II) (1.2 mg, 4.6 µmol). After three vacuum/argon cycles, reagents were dissolved in anhydrous dioxane (5 mL). The resulting mixture was stirred at 90 °C for 16 hours. Reaction mixture was cooled to room temperature. Solvent was evaporated off, and the residue was purified by column chromatography (SiO_2, DCM/hexanes, 2:3).

Maroon solid. Yield: 56% (42 mg). ^1H NMR (500 MHz, $CDCl_3$) δ 7.90 (d, *J* = 9.2 Hz, 2H), 7.82 (d, *J* = 1.4 Hz, 2H), 7.70 (d, *J* = 9.1 Hz, 2H), 7.57 (dd, *J* = 35.9, 8.2 Hz, 8H), 7.34 (dd, *J* = 9.1, 1.6 Hz, 2H), 7.19 (d, *J* = 9.1 Hz, 2H), 7.12 (d, *J* = 8.0 Hz, 4H), 7.08 - 6.99 (m, 12H), 6.98 (d, *J* = 8.0 Hz, 4H), 6.78 (d, *J* = 6.9 Hz, 8H), 2.78 (t, *J* = 7.6 Hz, 4H), 1.88 - 1.75 (quintet, *J* = 7.6 Hz, 4H), 1.53 - 1.30 (m, 20H), 0.92 (t, *J* = 6.7 Hz, 6H) ppm. ^{13}C NMR (126 MHz, $CDCl_3$) δ 147.1, 143.4, 138.03, 138.01, 136.7, 135.1, 135.0, 131.1, 129.1, 128.9, 127.29, 127.26, 126.9, 124.5, 123.04, 123.01, 122.32, 120.5, 120.3, 118.3, 113.7, 111.6, 106.9, 88.1, 84.5, 31.0, 27.2, 27.1, 24.9, 24.6, 24.5, 18.0 ppm. ^{10}B NMR (54 MHz, $CDCl_3$) δ 46.1 ppm. HRMS (APCI): *m/z* calculated for $C_{94}H_{81}B_2N_6$: 1315.6709 [M+H]$^{\cdot+}$; found: 1315.6750.

Synthesis of compound 1l

The Schlenk flask was charged with **2g** (104 mg, 92 mmol), 4-ethynyl-N,N-dimethylaniline (13 mg, 90 mmol), Cs_2CO_3 (156 mg, 478 mmol), XPhos (13.2 mg, 28 µmol) and bis(acetonitrile)dichloropalladium(II) (2,4mg, 9.2 µmol). After three vacuum/argon cycles, reagents were dissolved in anhydrous dioxane (10 mL). The resulting mixture was stirred at 90 °C for 16 hours. Reaction mixture was cooled to room temperature. Solvent were evaporated off, and the residue was purified by column chromatography (SiO_2, DCM/hexanes, 2:3).

Brown solid. Yield: 100mg (80%). ^1H NMR (500 MHz, $CDCl_3$) δ 7.88 (d, *J* = 9.1 Hz, 2H), 7.74 (d, *J* = 1.4 Hz, 2H), 7.65 (d, *J* = 9.1 Hz, 2H), 7.35 (d, *J* = 8.9 Hz, 4H), 7.32 (dd, *J* = 9.2, 1.7 Hz, 2H), 7.16 (d, *J* = 9.0 Hz, 2H), 7.11 (d, *J* = 8.0 Hz, 4H), 7.08 - 6.96 (m, 16H), 6.79 (d, *J* = 6.8 Hz, 8H), 6.64 (d, *J* = 8.3 Hz, 4H), 2.98 (s, 12H), 2.78 (t, *J* = 7.5 Hz, 4H), 1.82 (quintet, *J* = 7.7 Hz, 4H), 1.53 - 1.30 (m, 20H), 0.93 (t, *J* = 6.8 Hz, 6H) ppm. ^{13}C NMR (126 MHz, $CDCl_3$) δ 151.3, 150.2, 148.4, 142.5, 142.1, 141.1, 139.5, 139.5, 136.3, 134.0, 133.7, 132.7, 130.2, 129.2, 127.8, 127.0, 125.5, 124.8, 122.7, 120.6, 115.9, 111.8, 109.5, 92.5, 86.7, 40.2, 35.8, 32.0, 31.9, 29.6, 29.3, 29.3,

22.7, 14.1 ppm. ^{10}B NMR (54 MHz, $CDCl_3$) δ -123.7 ppm. HRMS (APCI): *m/z* calculated for $C_{96}H_{93}B_2N_6$: 1351.7648 $[M+H]^{\cdot+}$; found: 1351.7673.

3. Crystallographic data

Single crystal of **1a** suitable for x-ray diffraction analysis was obtained by slowly diffusing hexanes into its tetrahydrofuran solution. A total of 4584 frames were collected. The total exposure time was 61.12 hours. The frames were integrated with the Bruker SAINT software package using a narrow-frame algorithm.

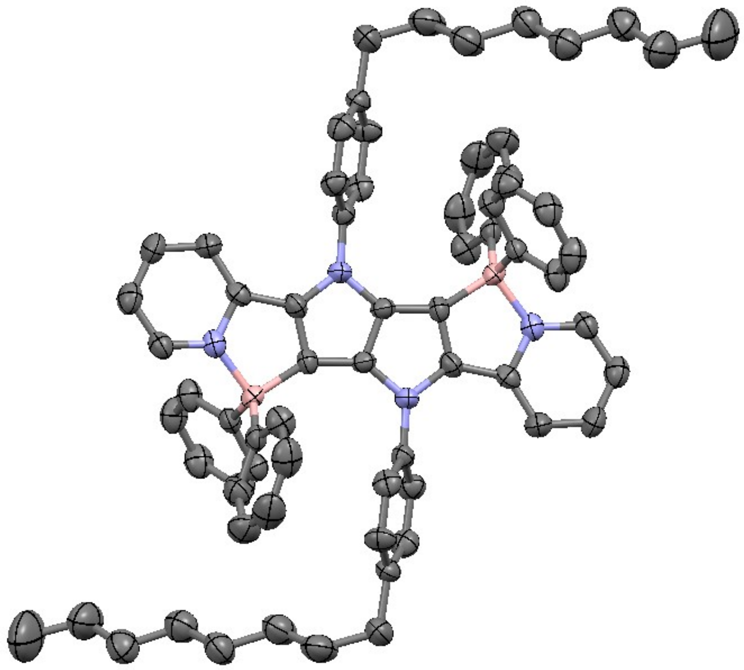

Fig. S1. Crystal structure of **1a** with thermal ellipsoids, shown at 50% probability. The hydrogen atoms in the structure were omitted for clarity

Table S1. Crystallographic data for 1a

Chemical Formula	$C_{68}H_{70}B_2N_4$	
Formula weight	964.90 g/mol	
Temperature	296(2) K	
Wavelength	1.54178 Å	
Crystal size	0.114 x 0.204 x 0.310 mm	
Crystal habit	fluorescent yellow plate	
Crystal system	monoclinic	
Space group	$P2_1/c$	
Unit cell dimensions	a = 16.0079(13) Å	α = 90°
	b = 12.8244(9) Å	β = 90.002(6)°
	c = 13.7166(11) Å	γ = 90°
Volume	2815.9(4) $Å^3$	
Z	2	
Diffractometer	Bruker APEX-II CCD	
Radiation source	fine-focus sealed tube	
Reflections collected	50300	
Independent reflections	4258 [R(int) = 0.1183]	
Tmax, Tmin	0.9460, 0.8620	

Absorption correction	numerical	
Refinement method	Full-matrix least-squares on F^2	
Function minimized	$\Sigma\ w(F_o^2 - F_c^2)^2$	
Data/restraints/parameters	4258 / 0 / 380	
Goodness-of-fit on F2	1.107	
Final R indices	1801 data; I>2σ(I)	R1 = 0.0838, wR2 = 0.1338
	all data	R1 = 0.2420, wR2 = 0.1690
Largest diff. Peak and hole	0.243 and -0.237 $eÅ^{-3}$	

4. Spectroscopic properties

When dissolved in toluene, pyridyl-substituted TAPPs **2a**-**2e** strongly absorb UV-light, with absorption maxima in the 375-394 nm region and extinction coefficient in the 23000 - 57000 $M^{-1}\cdot cm^{-1}$ range, whereas their emission maxima are observed between 419 - 436 nm (Table S2). Changing the substituent to quinoline (compounds **2f** and **2g**) leads to ca. 50 nm bathochromic shift of both absorption and emission maxima. This effect is much less pronounced in the case of isoquinoline substituted pyrrolo[3,2-*b*]pyrrole **2h**. All TAPPs exhibit moderate Stokes shifts in the 1730-3150 cm^{-1} with fluorescence quantum yields (Φ_{fl}) typically higher than 0.4, indicative of rigid structure undergoing small structural changes after absorption. The only exception to this trend is compound **2d** bearing bromine substituents which shows smaller emission yield, very likely due to the heavy atom effect, which, interestingly, is less marked for similar dibromo-derivative **2c**.

Table S2. Spectroscopic properties of compounds 2a-2h

Compound	Solvent	λ_{abs}^{max} [nm]	ε_{max} [$M^{-1}\cdot cm^{-1}$]	λ_{em}^{max} [nm]	Stokes shift [cm^{-1}]	Φ_{fl}
**2a**	toluene	377	46000	419	2660	0.66^{a}
**2b**	toluene	382	30900	421	2430	0.69^{a}
**2c**	toluene	394	29200	436	2440	0.60^{a}
**2d**	toluene	390	27100	428	2280	0.12^{a}
**2e**	toluene	375	30300	419	2800	0.56^{a}
**2f**	toluene	432	56600	467	1730	0.44^{b}
**2g**	toluene	443	46900	477	1610	0.48^{b}
**2h**	toluene	401	23700	459	3150	0.45^{a}

^{a}Determined with quinine sulfate in H_2SO_4 (0.5 M) as a standard. ^{b}Determined with coumarin 153 in ethanol as a standard.

Photostability measurements

Photostability of compounds **1a** and **2a** was determined through the variation in absorption of each sample at the appropriate absorption maximum wavelength (λ_{abs}) with respect to irradiation time. Toluene was selected as the solvent. Concentrations giving similar optical densities (A ≈ 1) were used. Quartz cells of samples were irradiated with a 300 W Xe lamp (Asahi spectra MAX-350, light power: 0.115 W/cm^2) for 180 min at 25 °C equipped with a UV/vis mirror module through a glass fiber. The absorption spectra were measured at appropriate times during the irradiation. BODIPY (difluoro{2-[1-(3,5-dimethyl-2*H*-pyrrol-2-ylidene-*N*)ethyl]-3,5-dimethyl-1*H*-pyrrolato-*N*}boron), DPP (2,5-dimethyl-3,6-bis(3,4-dimethoxyphenyl)pyrrolo[3,4-*c*]pyrrole-1,4(2*H*,5*H*)-dione) and Alexa Fluor 555 were used as references.

5. Absorption (blue line) and normalized emission (red line) spectra measured in toluene.

1a

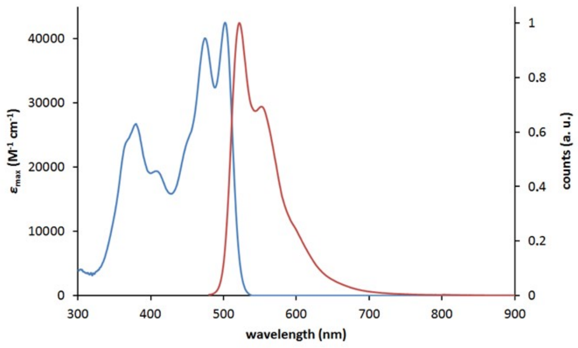

1b

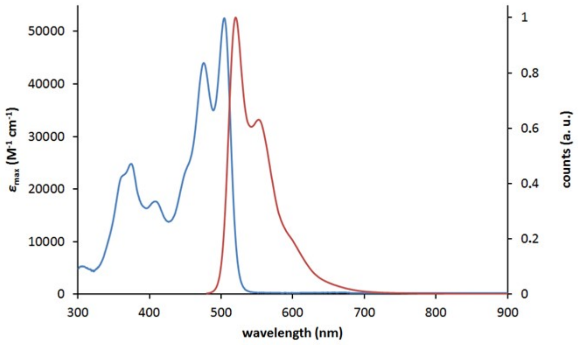

1c

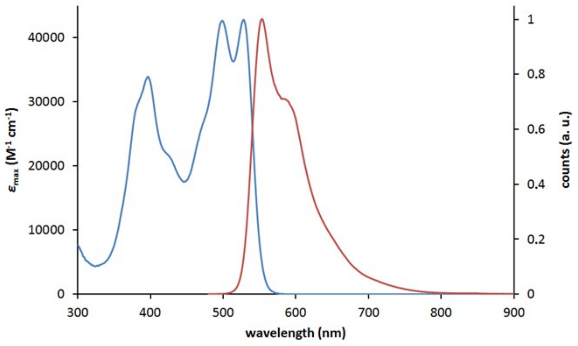

1d

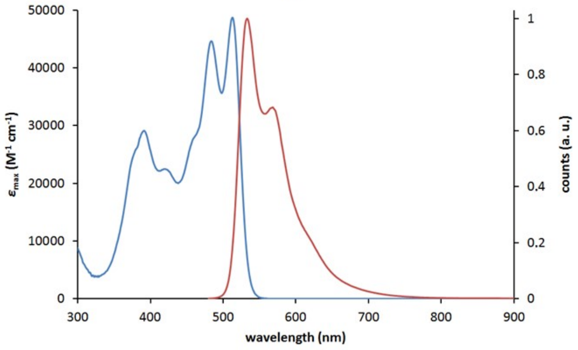

1e

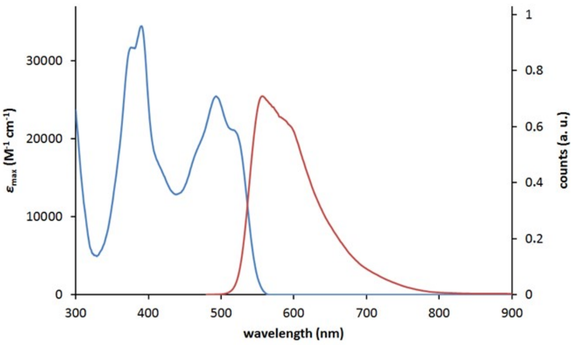

1f

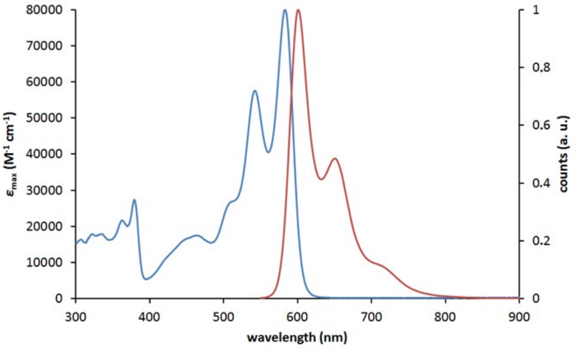

1g

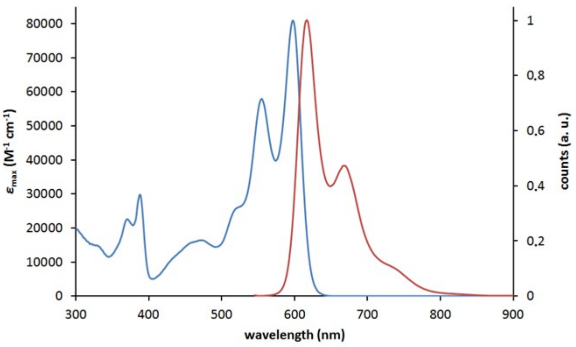

1h

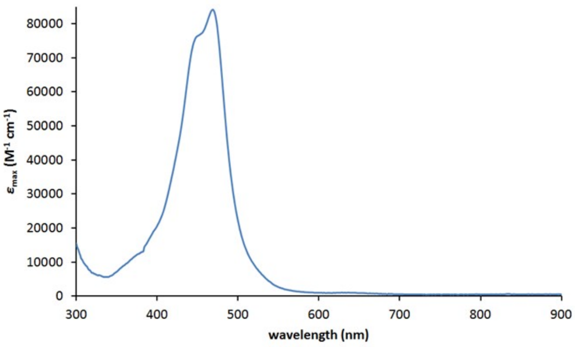

1i

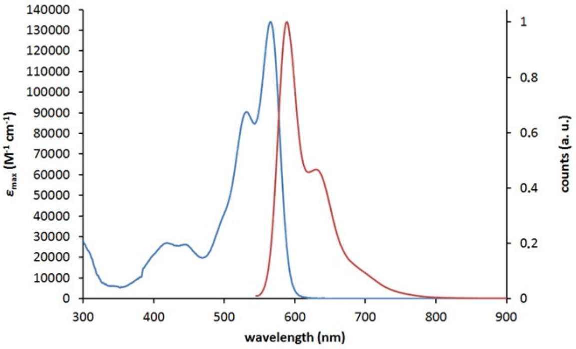

1j

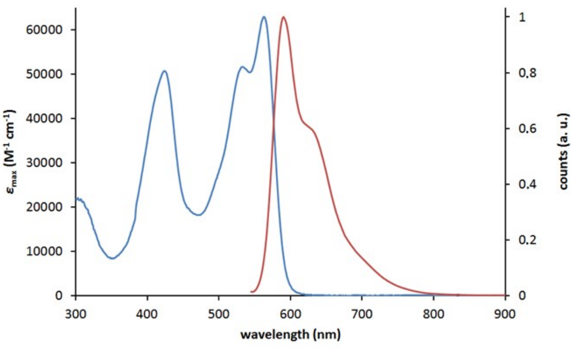

1k

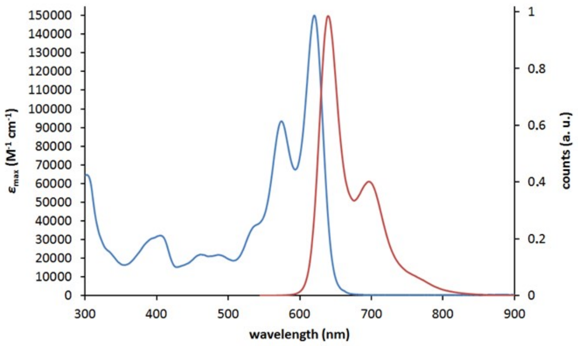

εmax (M-1 cm-1)
counts (a. u.)
wavelength (nm)
150000
140000
130000
120000
110000
100000
90000
80000
70000
60000
50000
40000
30000
20000
10000
0
1
0,8
0,6
0,4
0,2
0
300
400
500
600
700
800
900

1l

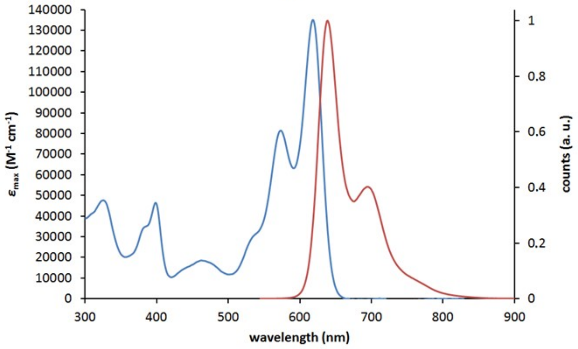

εmax (M-1 cm-1)
counts (a. u.)
wavelength (nm)
140000
130000
120000
110000
100000
90000
80000
70000
60000
50000
40000
30000
20000
10000
0
1
0,8
0,6
0,4
0,2
0
300
400
500
600
700
800
900

2b

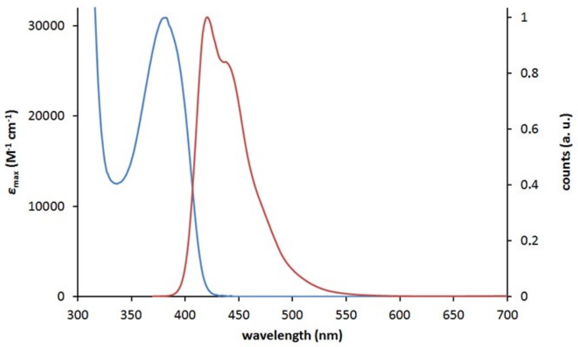

εmax (M-1 cm-1)
counts (a. u.)
wavelength (nm)
30000
20000
10000
0
1
0,8
0,6
0,4
0,2
0
300
350
400
450
500
550
600
650
700

2c

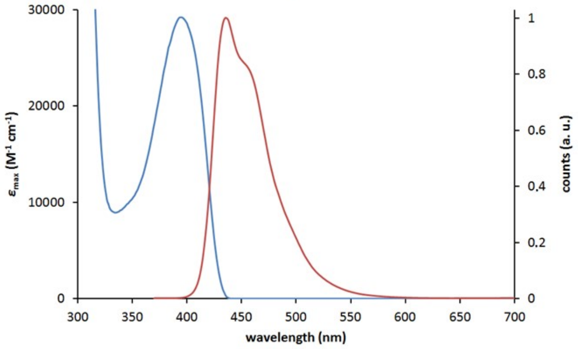

εmax (M-1 cm-1)
counts (a. u.)
wavelength (nm)
30000
20000
10000
0
1
0,8
0,6
0,4
0,2
0
300
350
400
450
500
550
600
650
700

2d

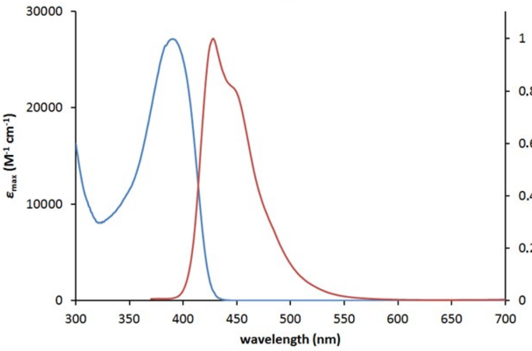
30000
20000
10000
0
$ε_{max}$ (M^{-1} cm^{-1})
1
0,8
0,6
0,4
0,2
0
counts (a. u.)
300 350 400 450 500 550 600 650 700
wavelength (nm)

2e

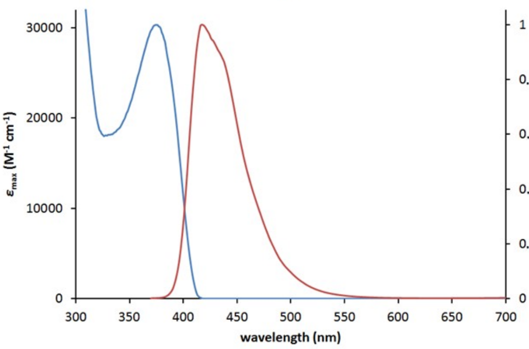
30000
20000
10000
0
$ε_{max}$ (M^{-1} cm^{-1})
1
0,8
0,6
0,4
0,2
0
counts (a. u.)
300 350 400 450 500 550 600 650 700
wavelength (nm)

2f

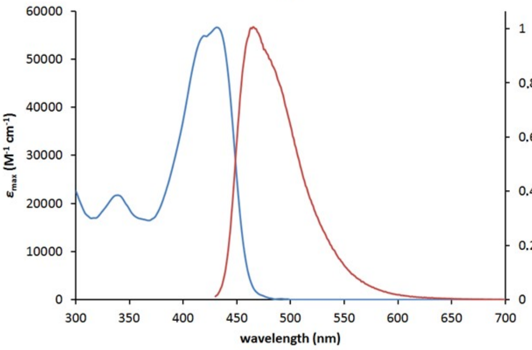
60000
50000
40000
30000
20000
10000
0
$ε_{max}$ (M^{-1} cm^{-1})
1
0,8
0,6
0,4
0,2
0
counts (a. u.)
300 350 400 450 500 550 600 650 700
wavelength (nm)

2g

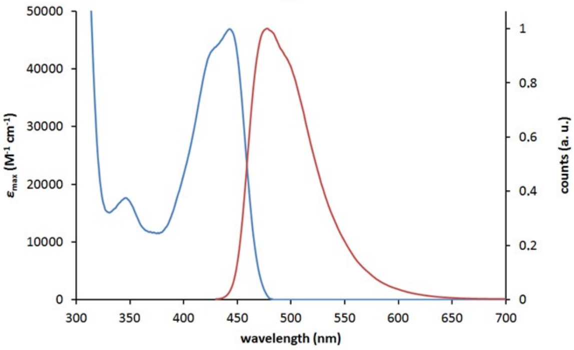
50000
40000
30000
20000
10000
0
$ε_{max}$ (M^{-1} cm^{-1})
1
0,8
0,6
0,4
0,2
0
counts (a. u.)
300 350 400 450 500 550 600 650 700
wavelength (nm)

2h

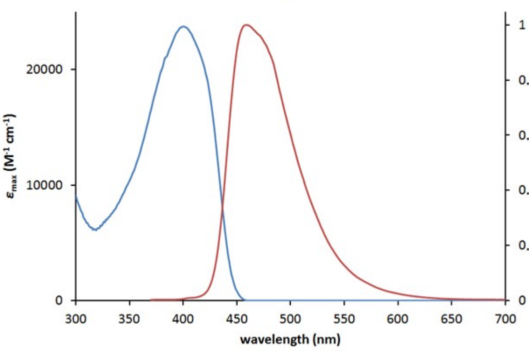
20000
10000
0
$ε_{max}$ (M^{-1} cm^{-1})
1
0,8
0,6
0,4
0,2
0
counts (a. u.)
300 350 400 450 500 550 600 650 700
wavelength (nm)

6. Electrochemical properties

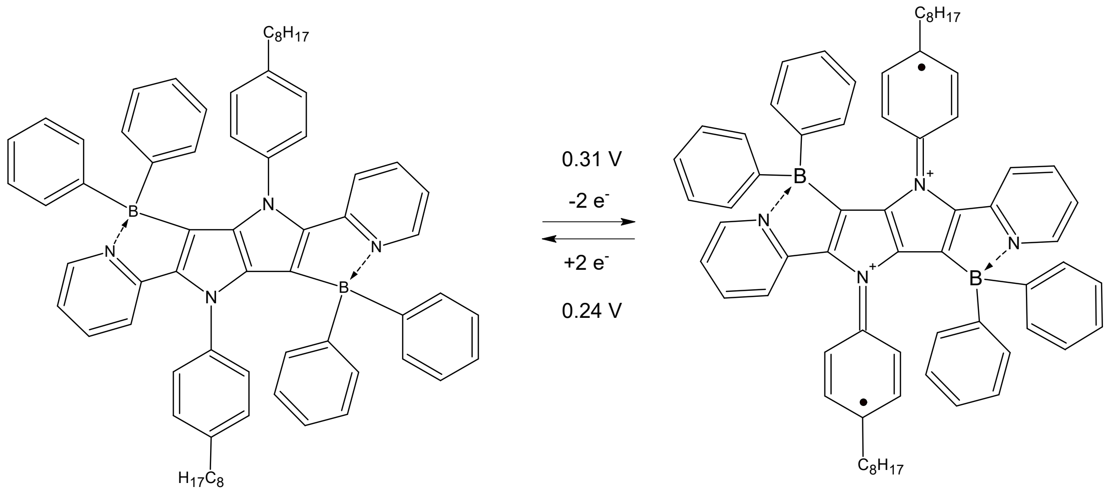

1a **(1a)2•+**

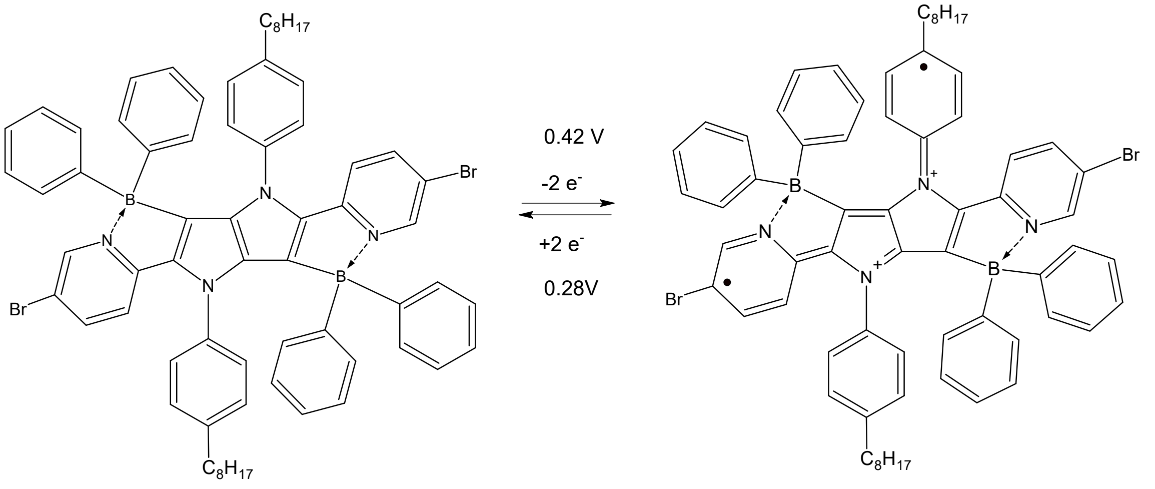

1c **(1c)2•+**

Scheme S1. Structural changes during the oxidation of **1a** and **1c** under the polarization of the first reversible oxidation peak

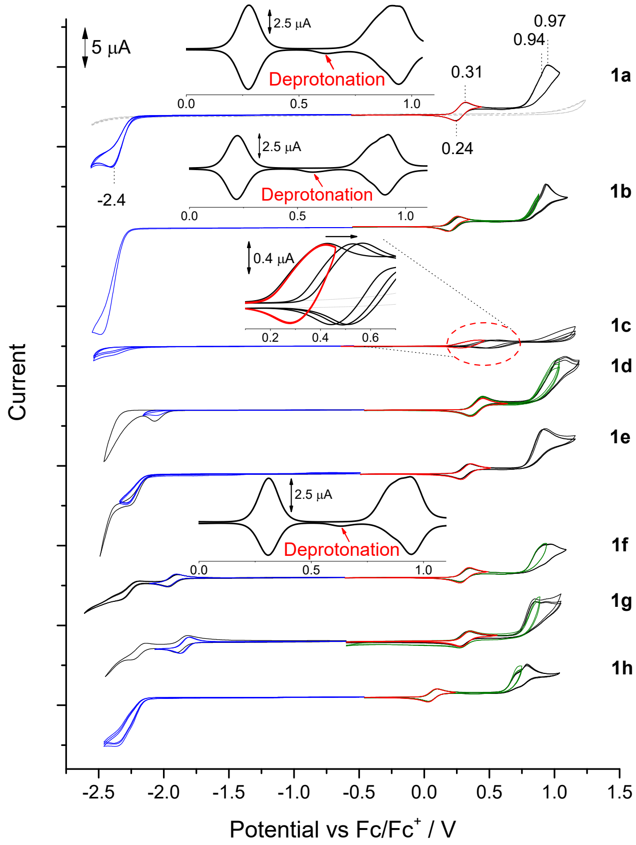

Fig. S2. Cyclic voltammograms in the solution of BN-TAPP-series compounds (1 mM) in 0.1 M Bu4NBF4/DCM. Oxidation (red, black CVs), reduction (blue, black CVs), second oxidation (green, black CVs).

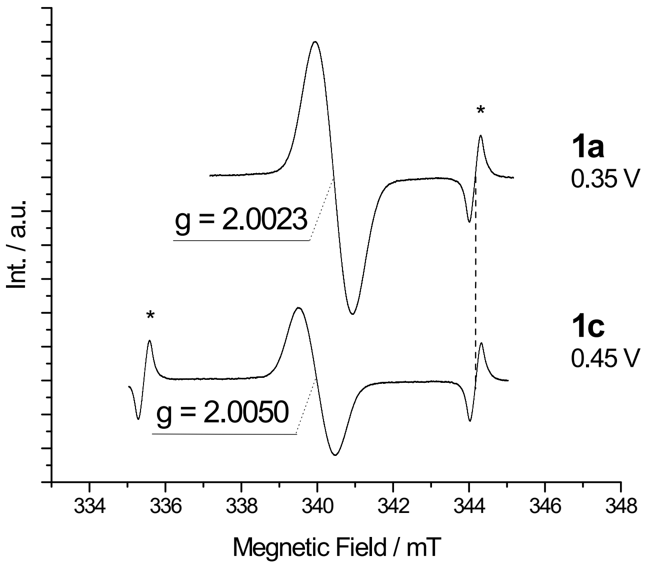

Fig. S3. In-situ ESR spectra of **1a** and **1c** in 0.1 M Bu_4NBF_4/DCM under the polarization of the first reversible oxidation peak; 1 mT, Amp. 50, 1mW.

7. Calculations

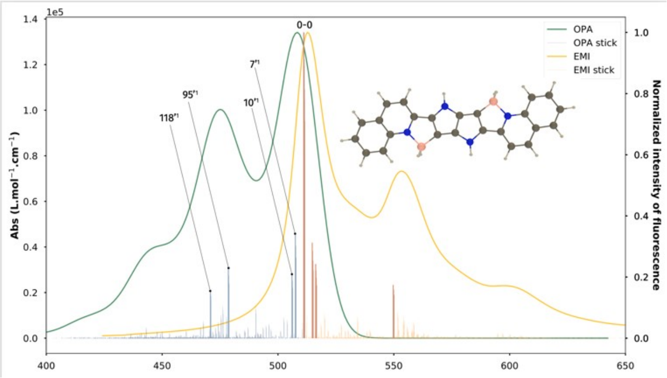

Fig. S4. Vibrationally resolved one-photon absorption and emission spectra for a model of compound of **1f**

8. ^{1}H, ^{13}C, ^{19}F and ^{10}B NMR spectra

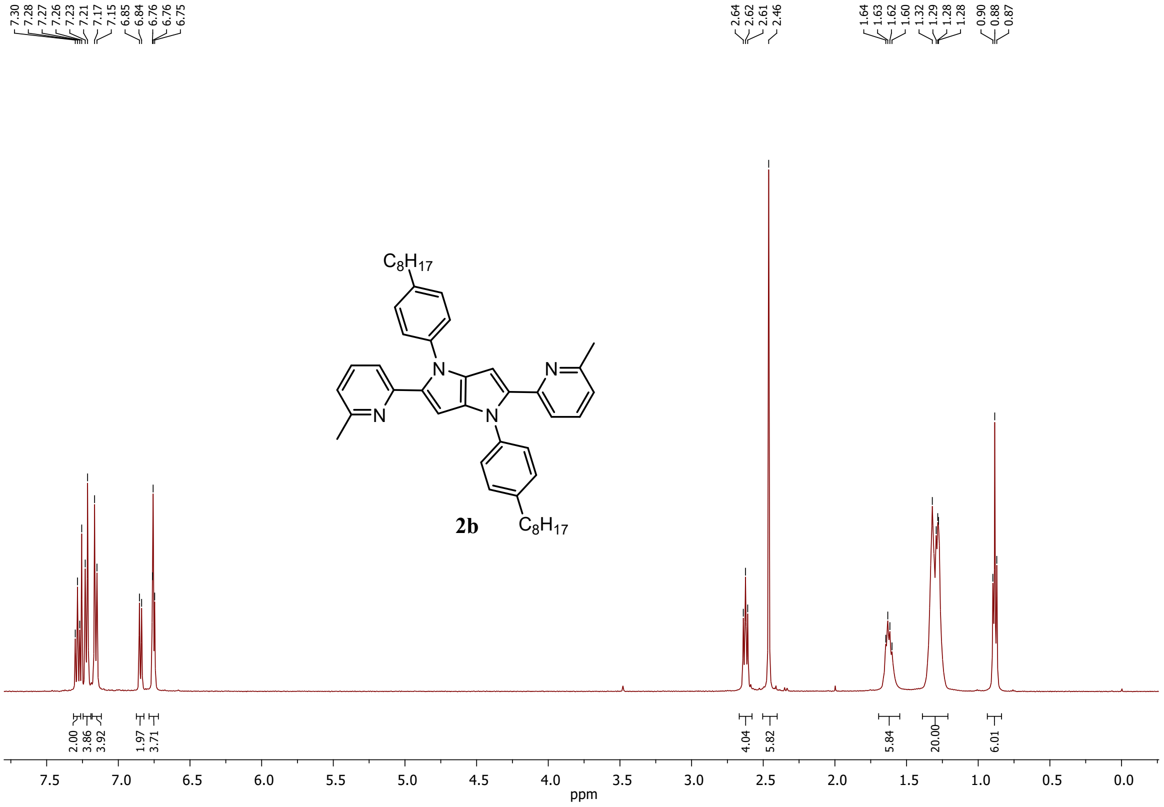

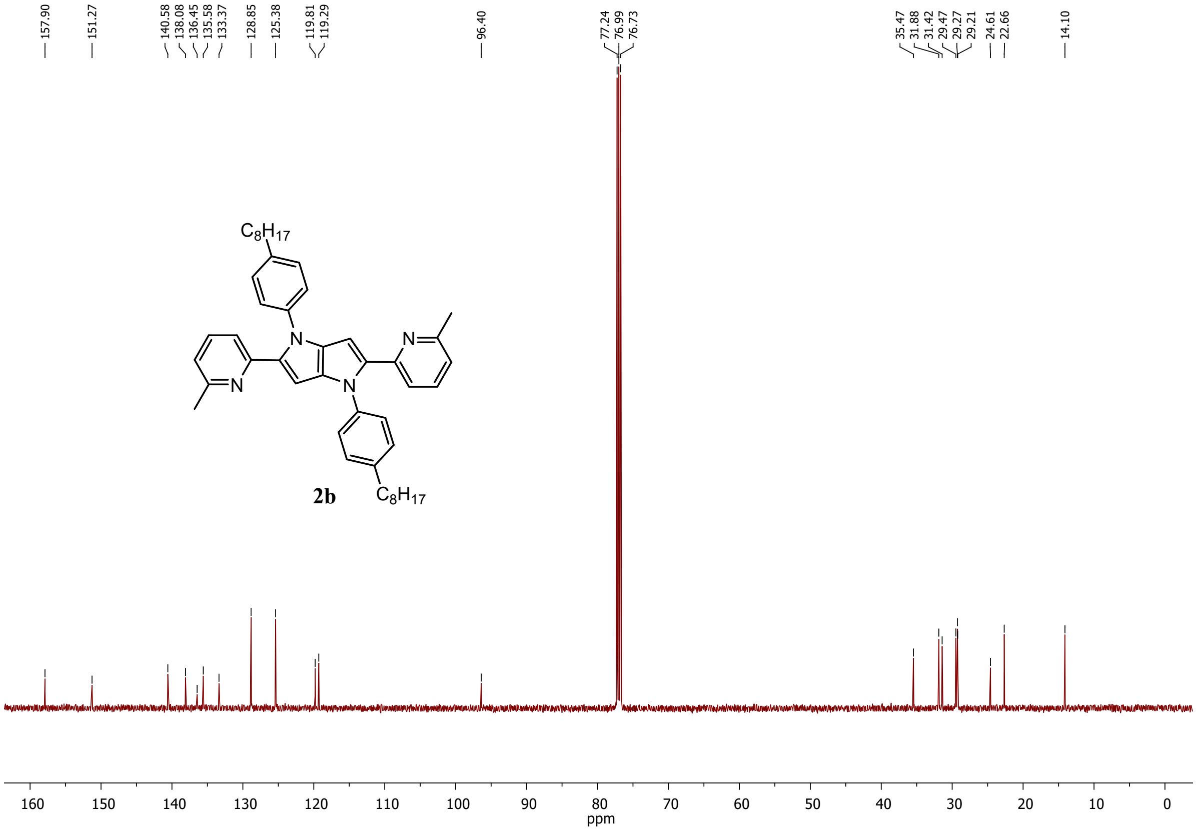

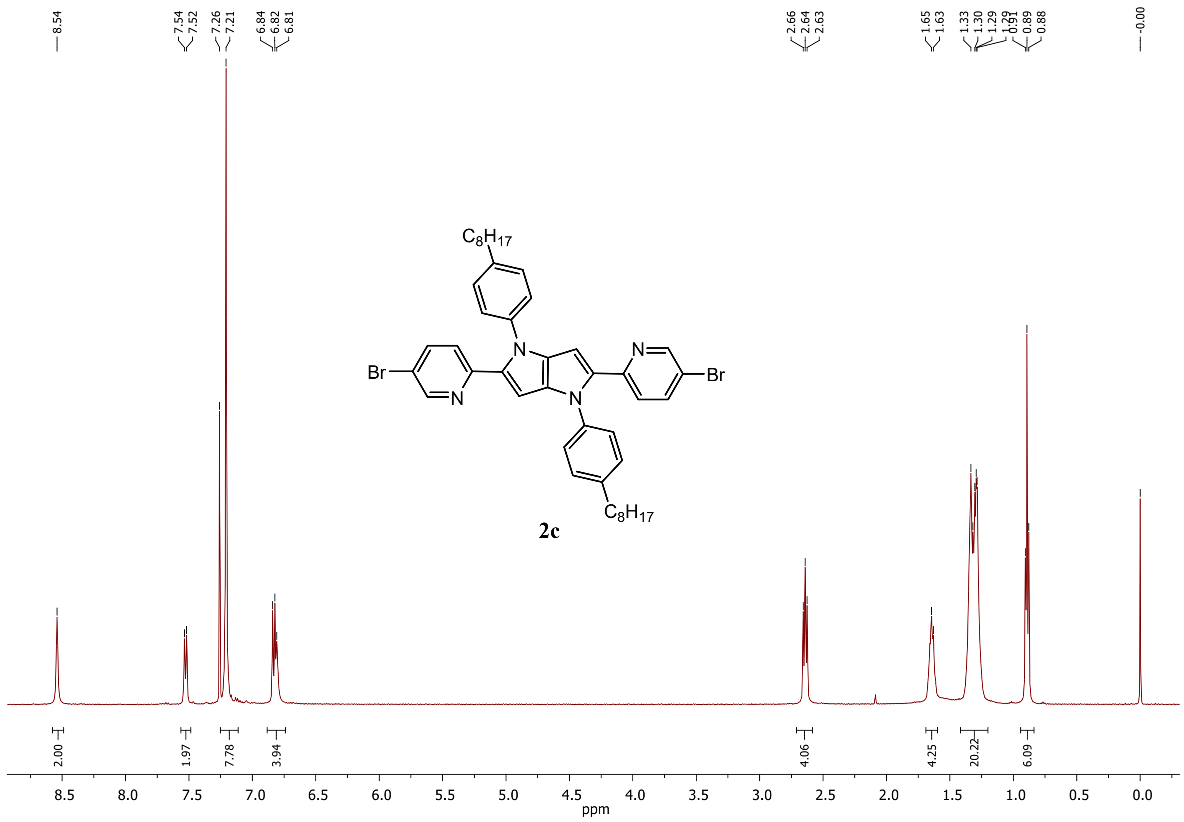
C8H17
Br
N
2c
ppm

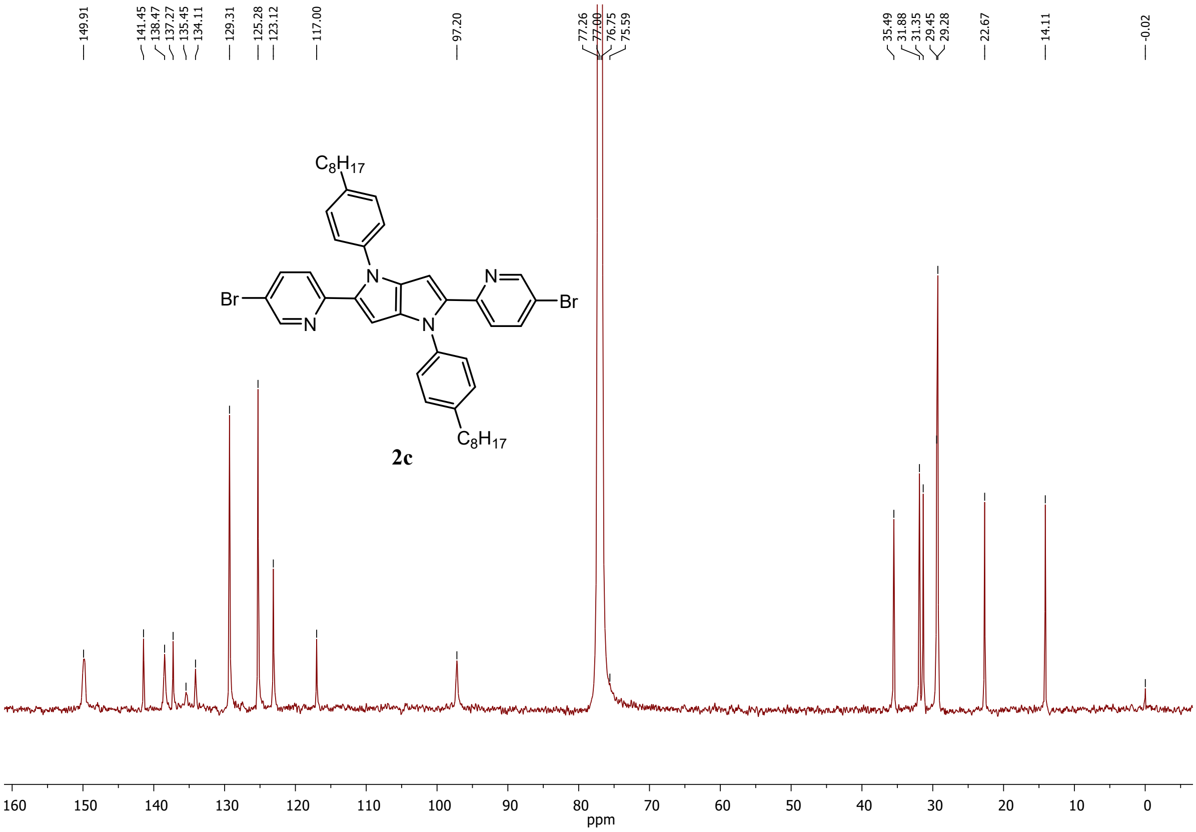
C8H17
Br
N
2c
ppm

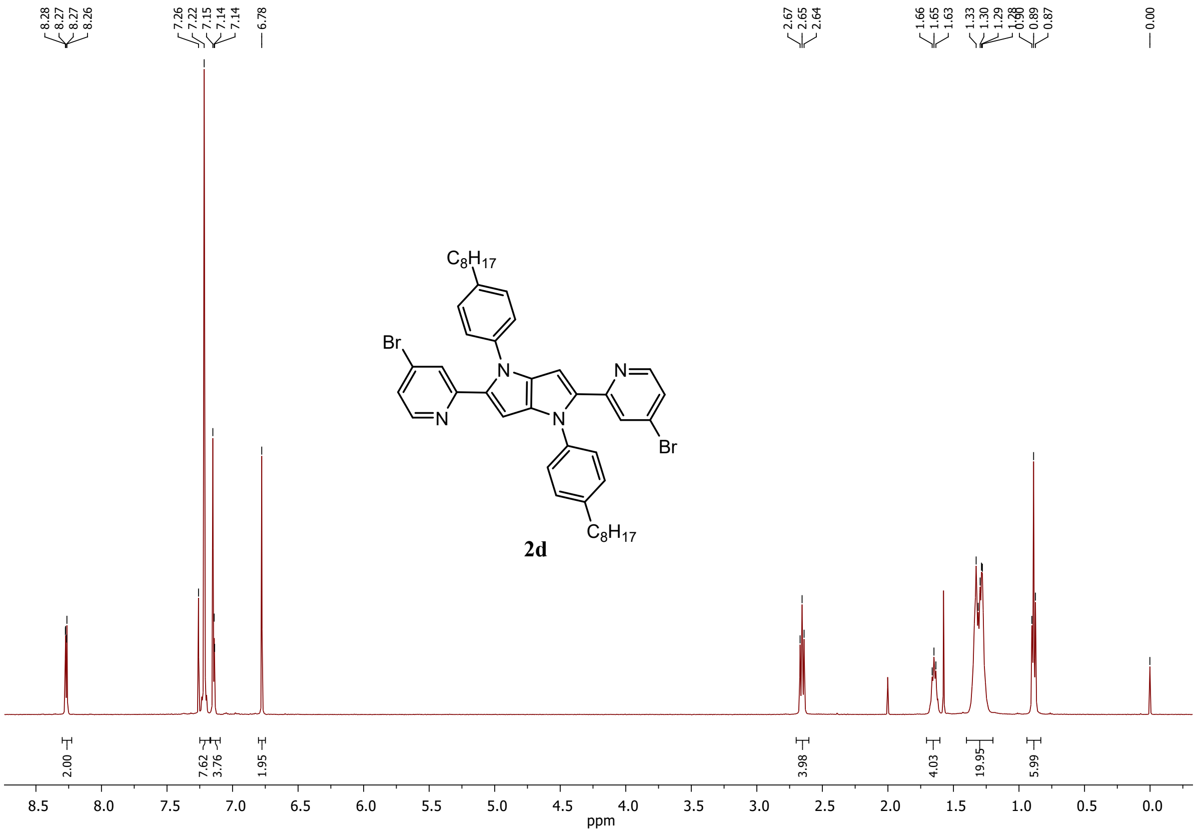
8.28
8.27
8.27
8.26
7.26
7.22
7.15
7.14
7.14
6.78
2.67
2.65
2.64
1.66
1.65
1.63
1.33
1.30
1.29
0.89
0.87
0.00
Br
N
N
N
N
Br
C_8H_{17}
C_8H_{17}
2d
2.00
7.62
3.76
1.95
3.98
4.03
19.95
5.99
8.5
8.0
7.5
7.0
6.5
6.0
5.5
5.0
4.5
4.0
3.5
3.0
2.5
2.0
1.5
1.0
0.5
0.0
ppm

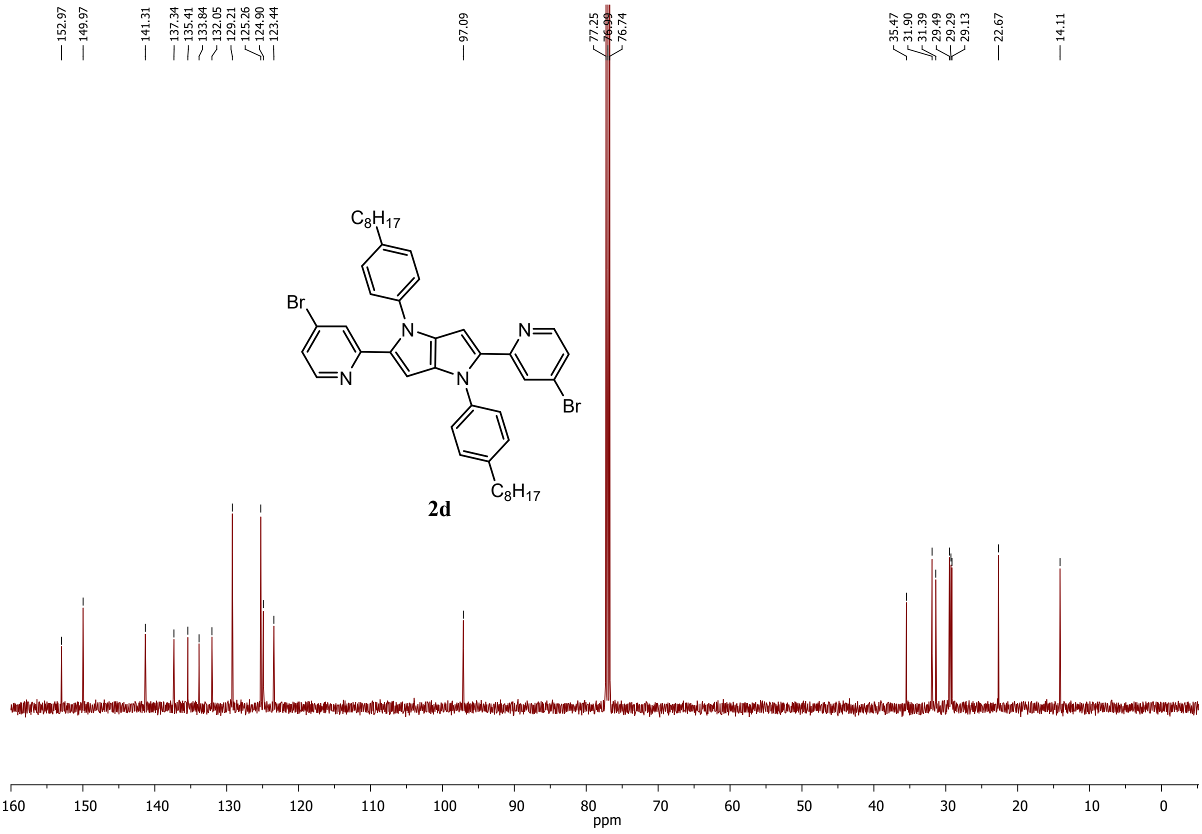
152.97
149.97
141.31
137.34
135.41
133.84
132.05
129.21
125.26
124.90
123.44
97.09
77.25
76.99
76.74
35.47
31.90
31.39
29.49
29.29
29.13
22.67
14.11
Br
N
N
N
N
Br
C_8H_{17}
C_8H_{17}
2d
160
150
140
130
120
110
100
90
80
70
60
50
40
30
20
10
0
ppm

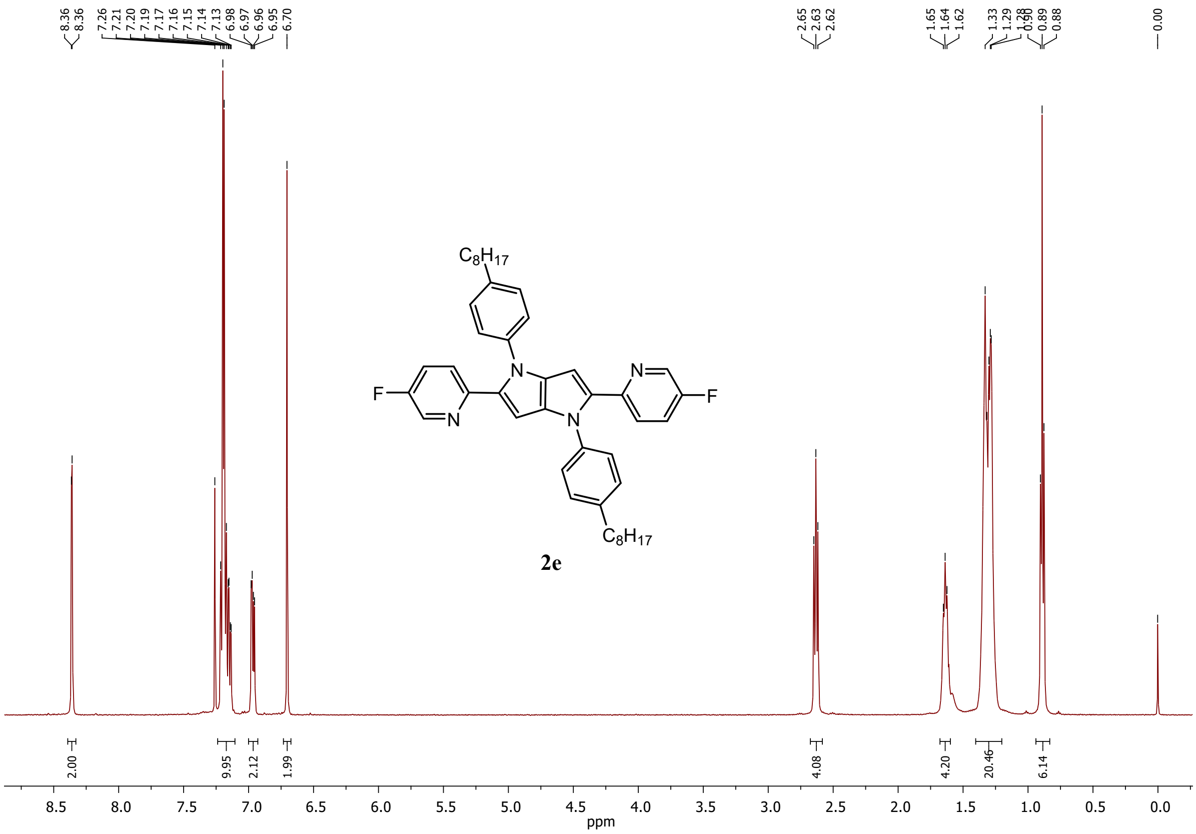
2e
ppm

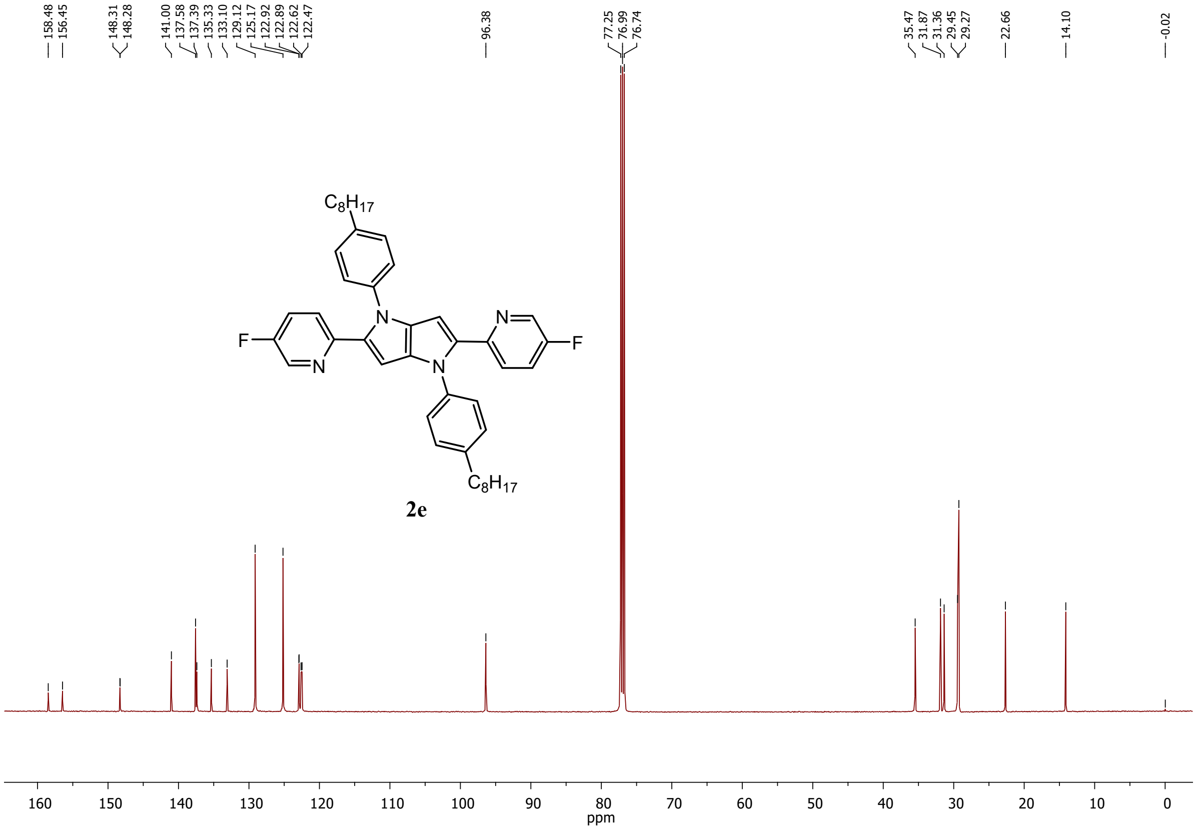
2e
ppm

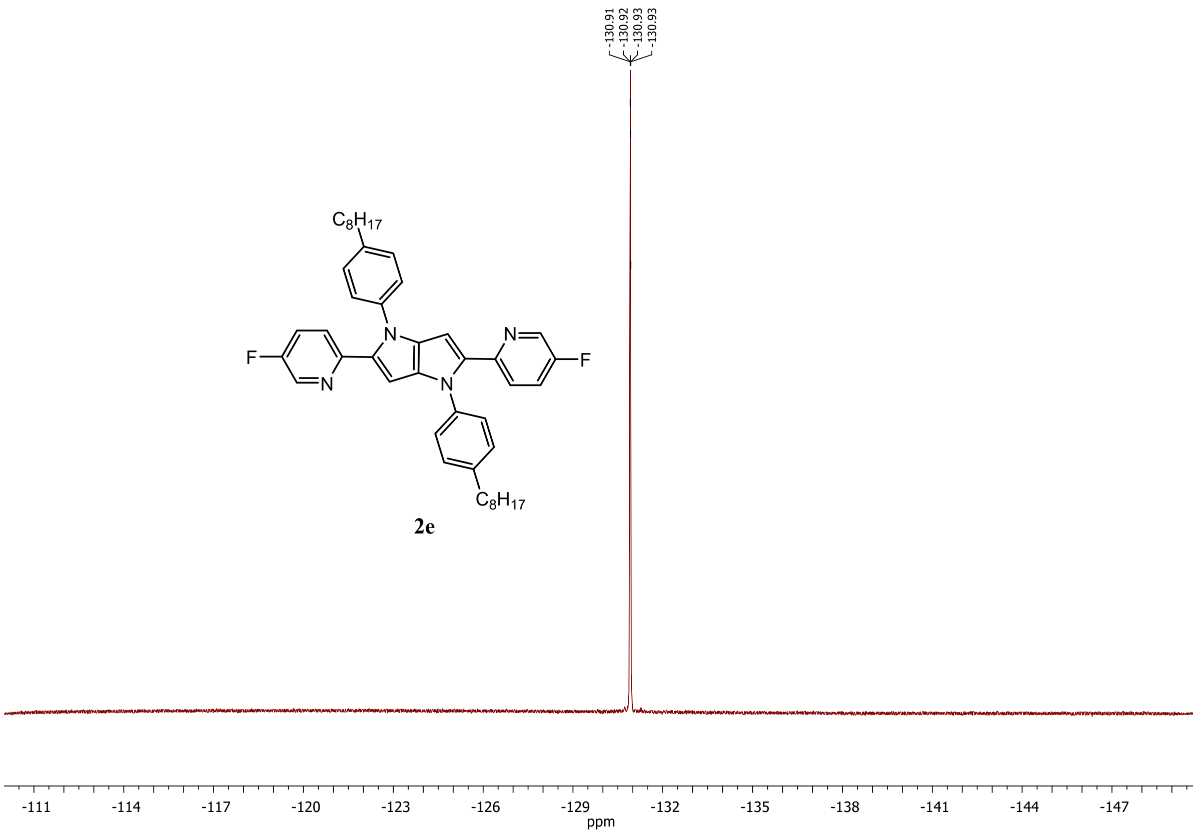
-130.91
-130.92
-130.93
-130.93
C_8H_{17}
F
N
N
N
N
F
C_8H_{17}
2e
-111 -114 -117 -120 -123 -126 -129 -132 -135 -138 -141 -144 -147
ppm

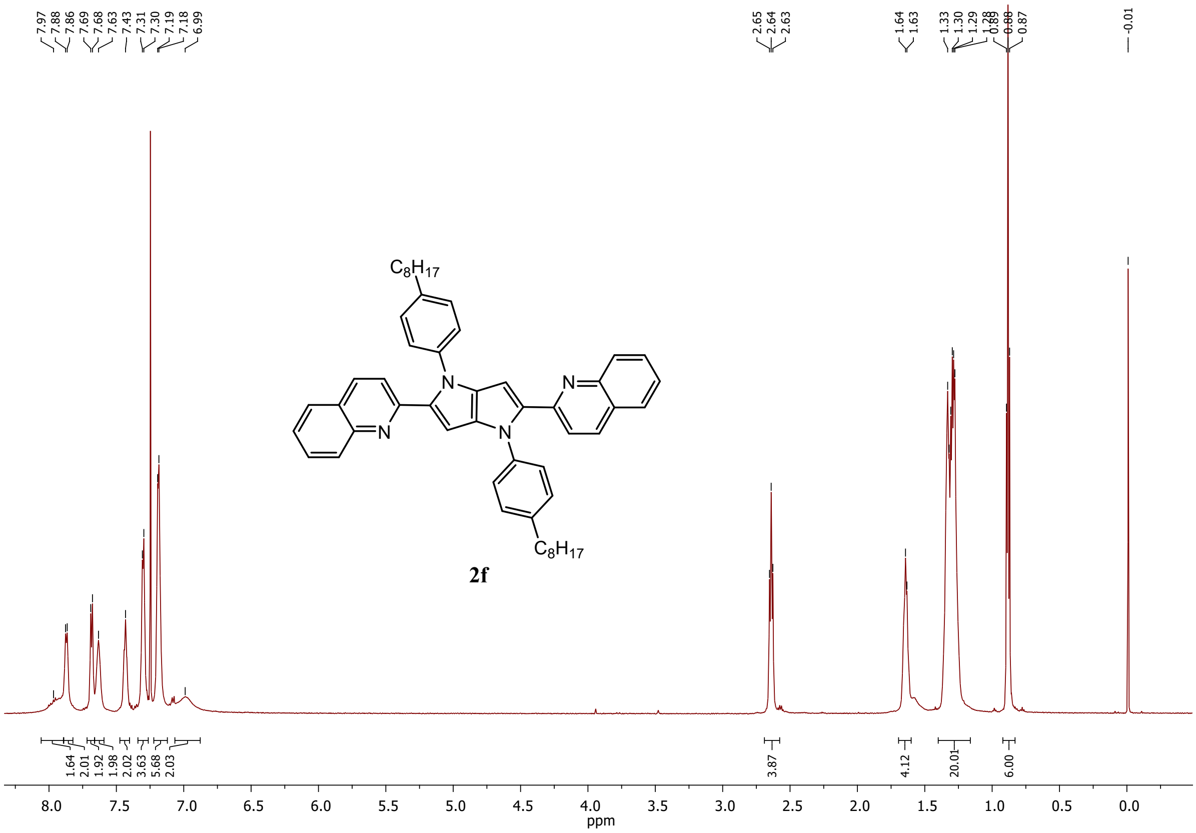
7.97
7.88
7.86
7.69
7.68
7.63
7.43
7.31
7.30
7.19
7.18
6.99
2.65
2.64
2.63
1.64
1.63
1.33
1.30
1.29
0.87
-0.01
C_8H_{17}
N
N
N
N
C_8H_{17}
2f
1.64
2.01
1.92
1.98
2.02
3.63
5.68
2.03
3.87
4.12
20.01
6.00
8.0 7.5 7.0 6.5 6.0 5.5 5.0 4.5 4.0 3.5 3.0 2.5 2.0 1.5 1.0 0.5 0.0
ppm

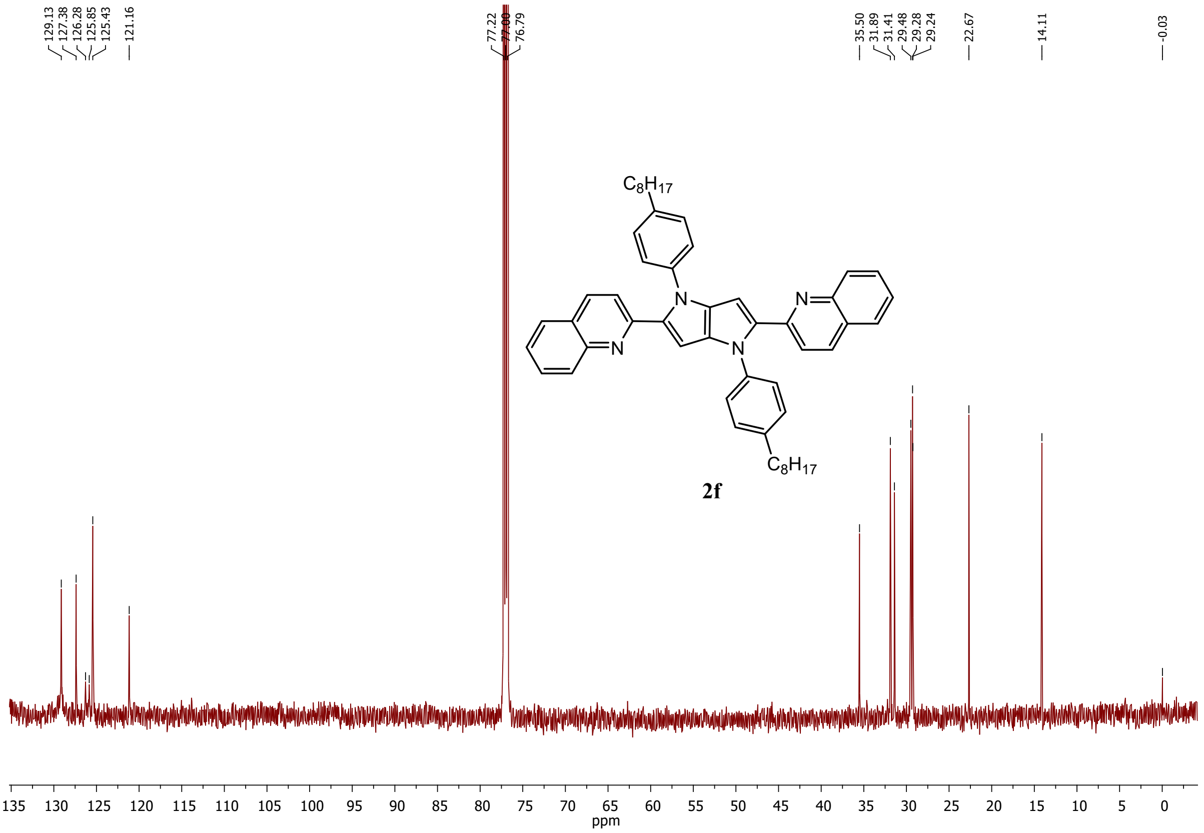
129.13
127.38
126.28
125.85
125.43
121.16
77.22
77.00
76.79
35.50
31.89
31.41
29.48
29.28
29.24
22.67
14.11
-0.03
C8H17
N
2f
ppm

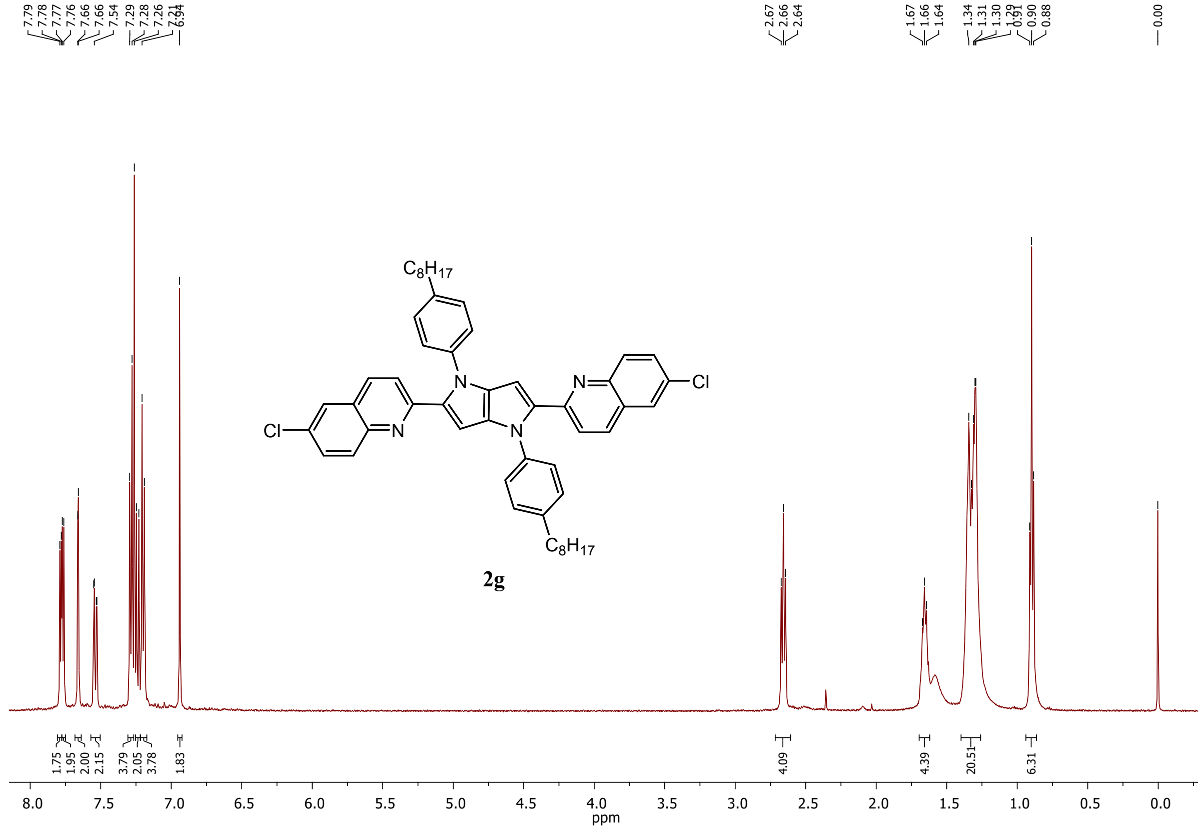
7.79
7.78
7.77
7.76
7.66
7.66
7.54
7.29
7.28
7.26
7.21
6.94
2.67
2.66
2.64
1.67
1.66
1.64
1.34
1.31
1.30
1.29
0.91
0.90
0.88
0.00
C8H17
N
Cl
2g
1.75
1.95
2.00
2.15
3.79
2.05
3.78
1.83
4.09
4.39
20.51
6.31
ppm

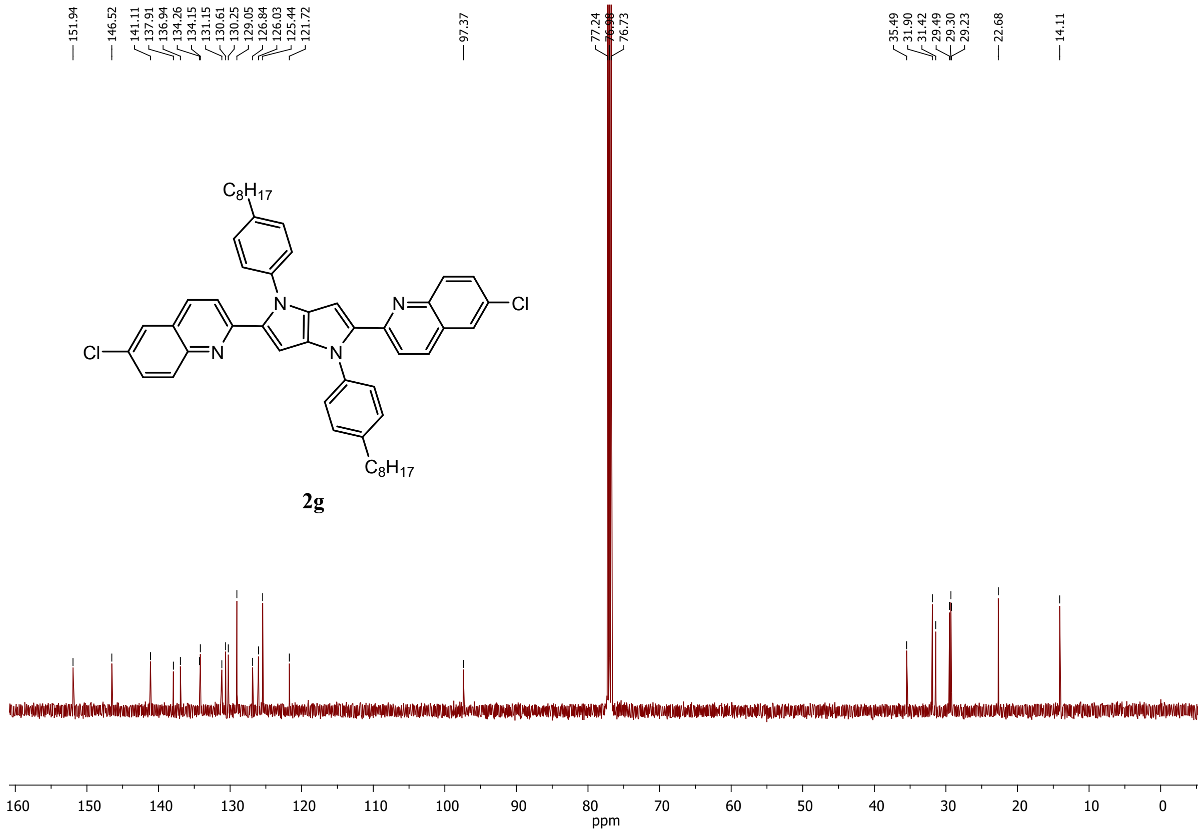
151.94
146.52
141.11
137.91
136.94
134.26
134.15
131.15
130.61
130.25
129.05
126.84
126.03
125.44
121.72
97.37
77.24
76.73
35.49
31.90
31.42
29.49
29.30
29.23
22.68
14.11
C_8H_{17}
N
Cl
ppm

2g

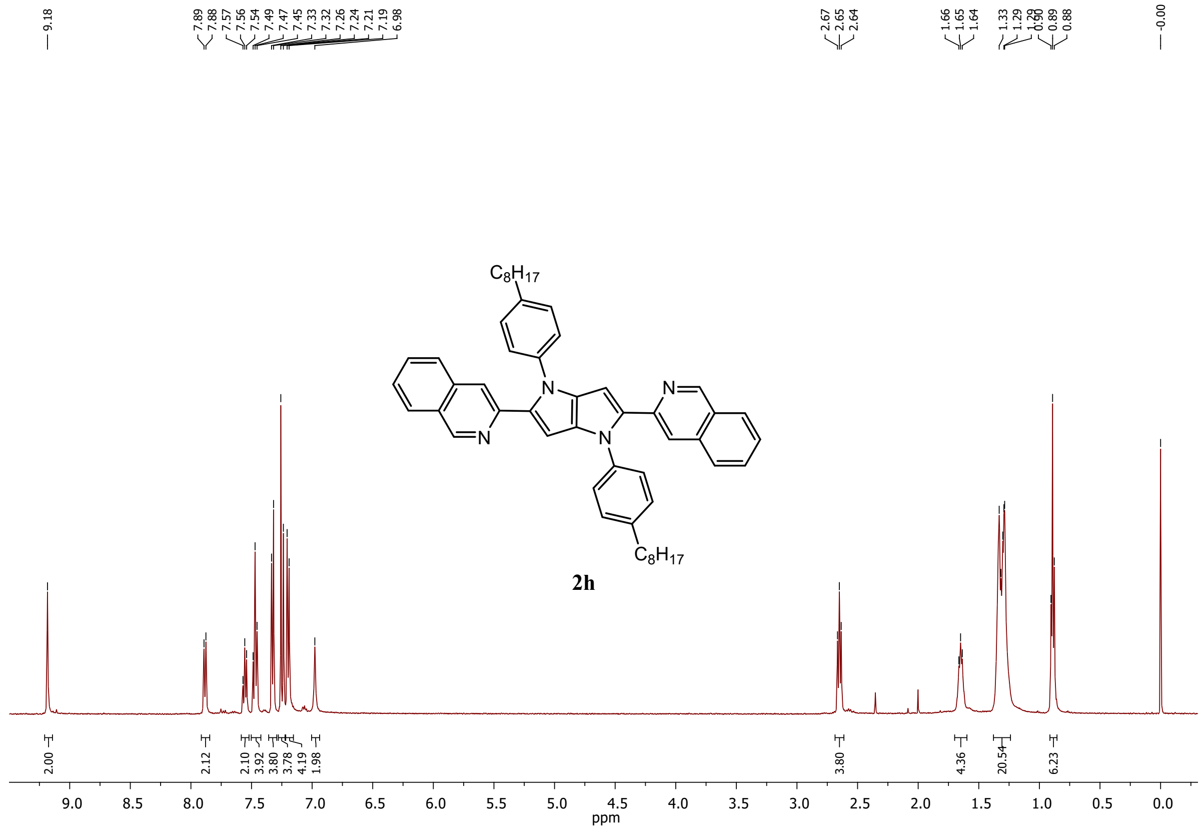
9.18
7.89
7.88
7.57
7.56
7.54
7.49
7.47
7.45
7.33
7.32
7.26
7.24
7.21
7.19
6.98
2.67
2.65
2.64
1.66
1.65
1.64
1.33
1.29
0.89
0.88
-0.00
C_8H_{17}
N
2.00
2.12
2.10
3.92
3.80
3.78
4.19
1.98
3.80
4.36
20.54
6.23
ppm

2h

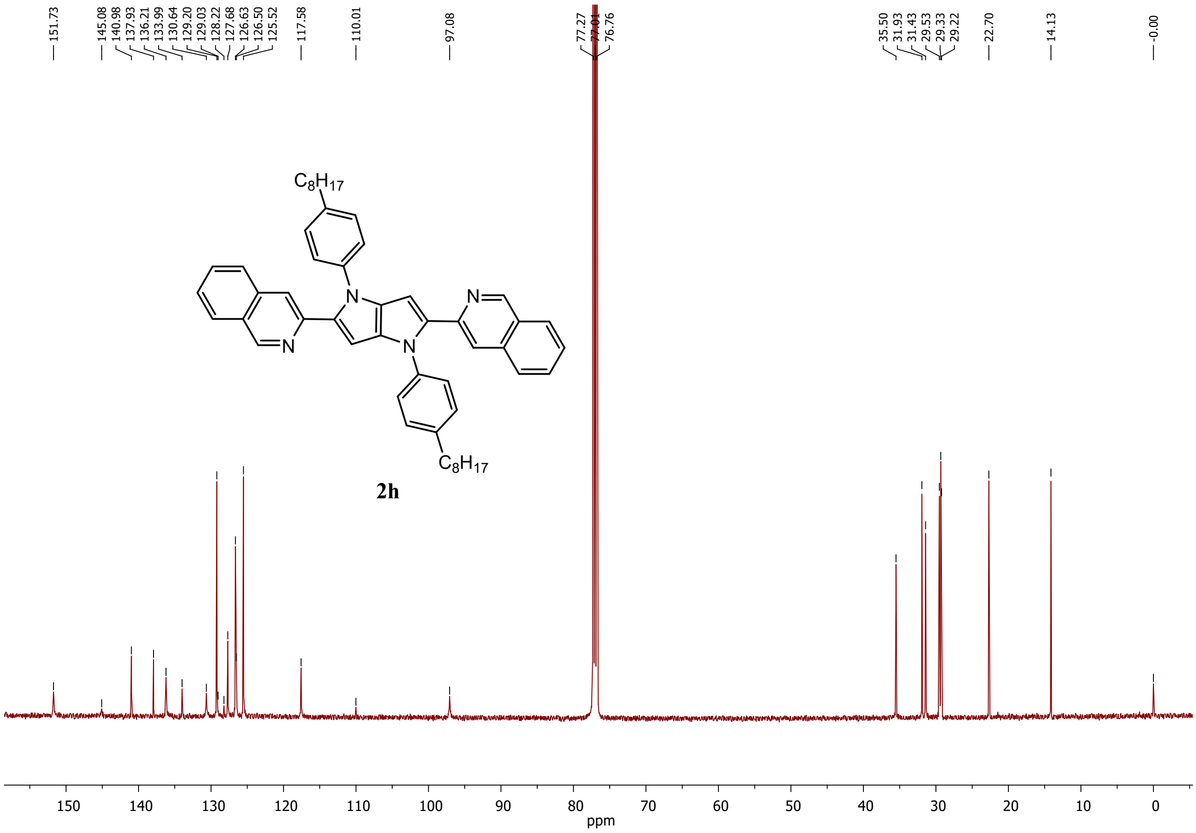
151.73
145.08
140.98
137.93
136.21
133.99
130.64
129.20
129.03
128.22
127.68
126.63
126.50
125.52
117.58
110.01
97.08
77.27
77.01
76.76
35.50
31.93
31.43
29.53
29.33
29.22
22.70
14.13
-0.00
C_8H_{17}
N
N
N
N
C_8H_{17}
2h
ppm

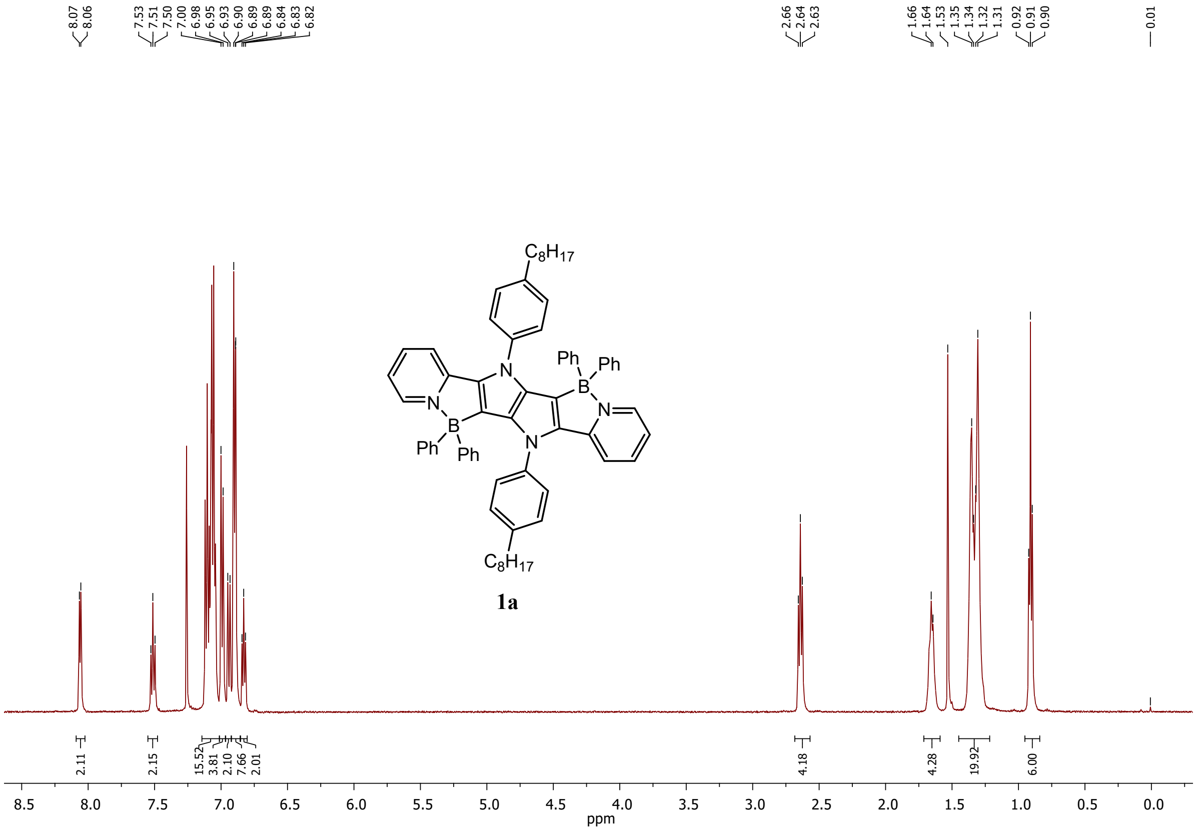
8.07
8.06
7.53
7.51
7.50
7.00
6.98
6.95
6.93
6.90
6.89
6.89
6.84
6.83
6.82
2.66
2.64
2.63
1.66
1.64
1.53
1.35
1.34
1.32
1.31
0.92
0.91
0.90
0.01
C_8H_{17}
Ph
Ph
B
N
N
N
N
B
Ph
Ph
C_8H_{17}
1a
2.11
2.15
15.52
3.81
2.10
7.66
2.01
4.18
4.28
19.92
6.00
ppm

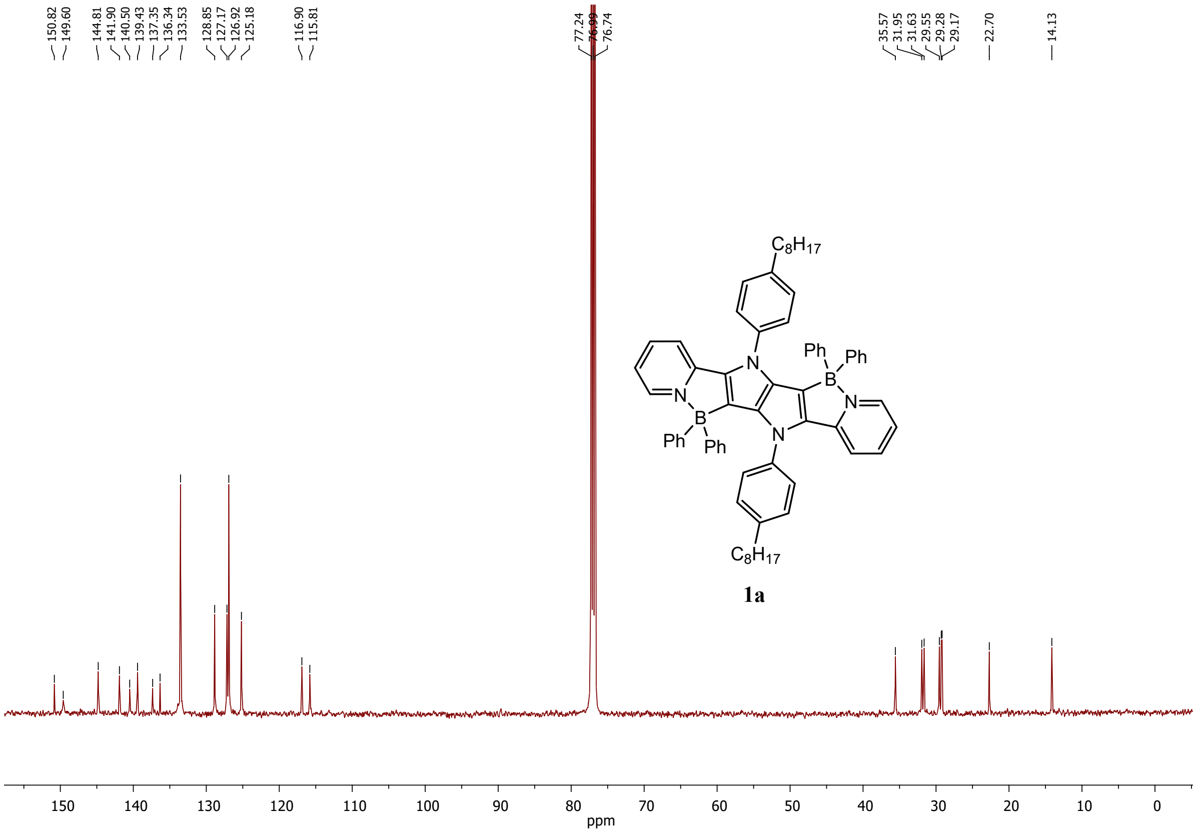

150.82
149.60
144.81
141.90
140.50
139.43
137.35
136.34
133.53
128.85
127.17
126.92
125.18
116.90
115.81
77.24
76.99
76.74
35.57
31.95
31.63
29.55
29.28
29.17
22.70
14.13
C_8H_{17}
Ph
B
N
1a
ppm

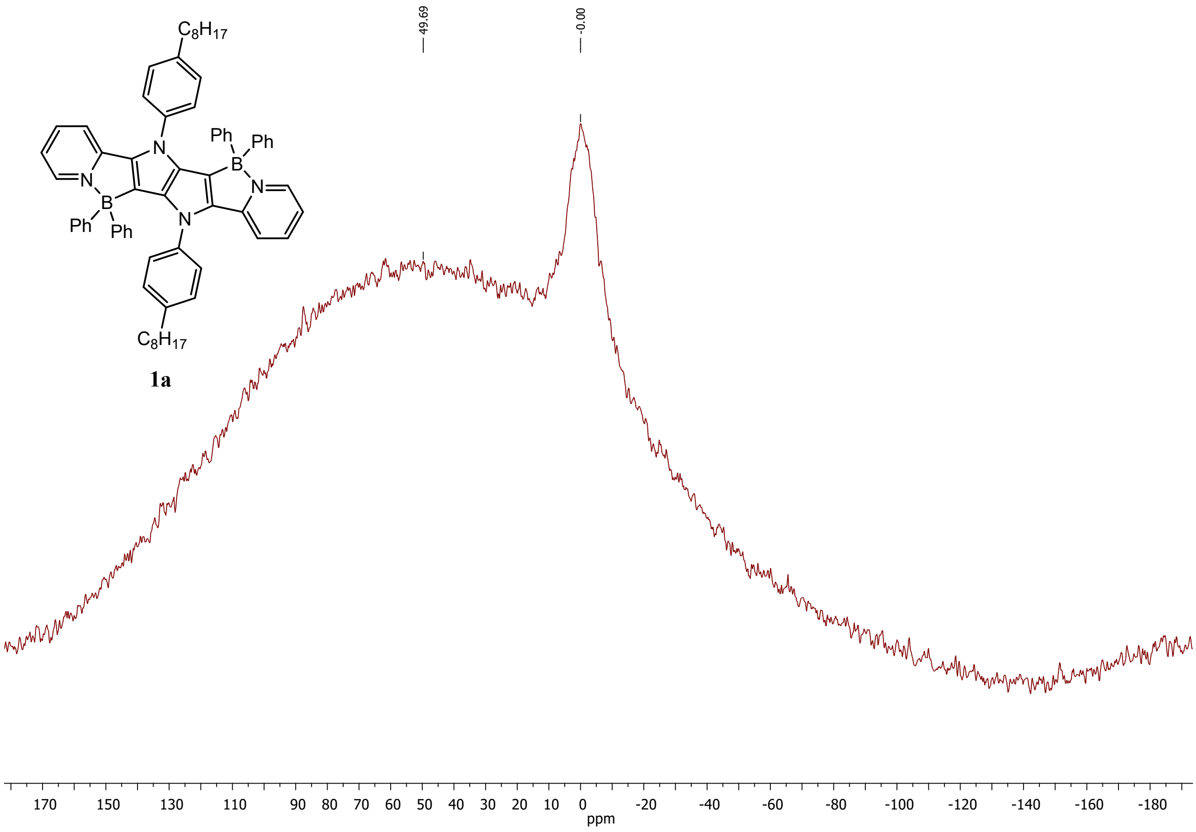

49.69
-0.00
C_8H_{17}
Ph
B
N
1a
ppm

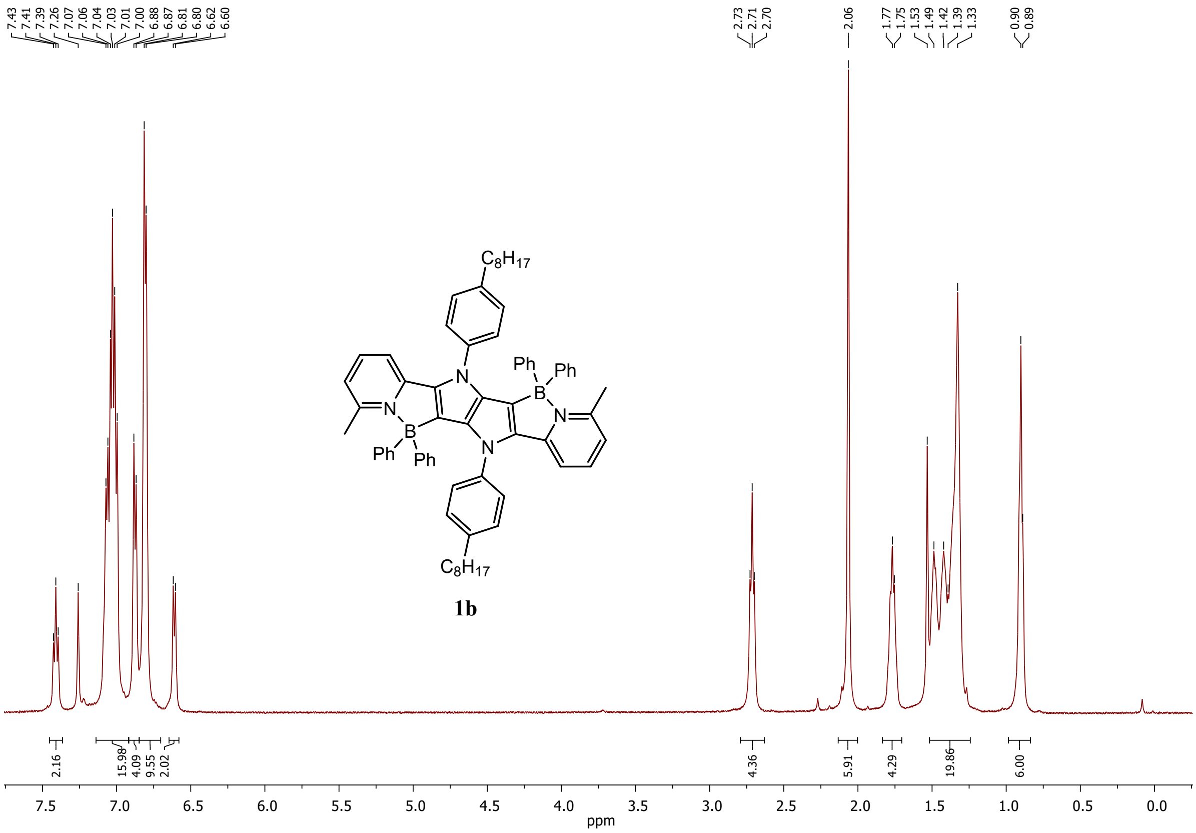
7.43
7.41
7.39
7.26
7.07
7.06
7.04
7.03
7.01
7.00
6.88
6.87
6.81
6.80
6.62
6.60
2.73
2.71
2.70
2.06
1.77
1.75
1.53
1.49
1.42
1.39
1.33
0.90
0.89
C_8H_{17}
Ph
B
N
1b
2.16
15.98
4.09
9.55
2.02
4.36
5.91
4.29
19.86
6.00
ppm

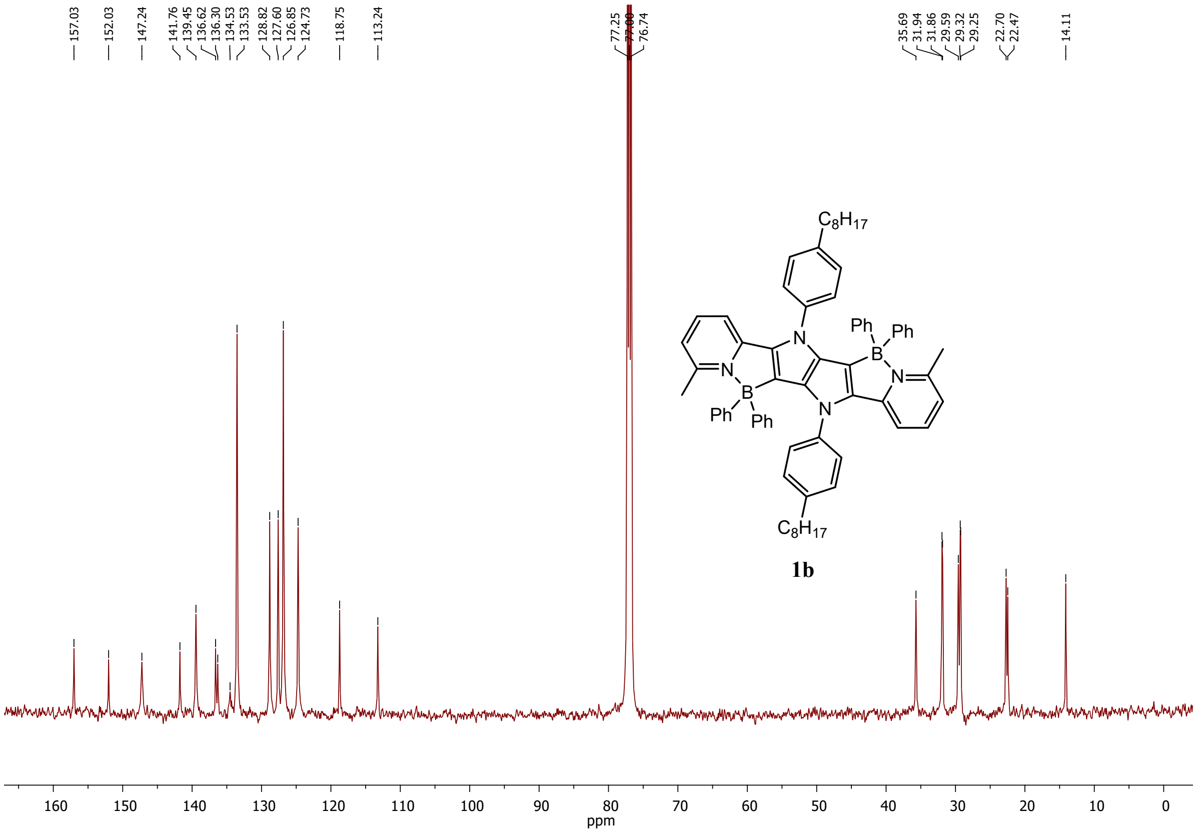
157.03
152.03
147.24
141.76
139.45
136.62
136.30
134.53
133.53
128.82
127.60
126.85
124.73
118.75
113.24
77.25
77.00
76.74
35.69
31.94
31.86
29.59
29.32
29.25
22.70
22.47
14.11
C_8H_{17}
Ph
B
N
1b
ppm

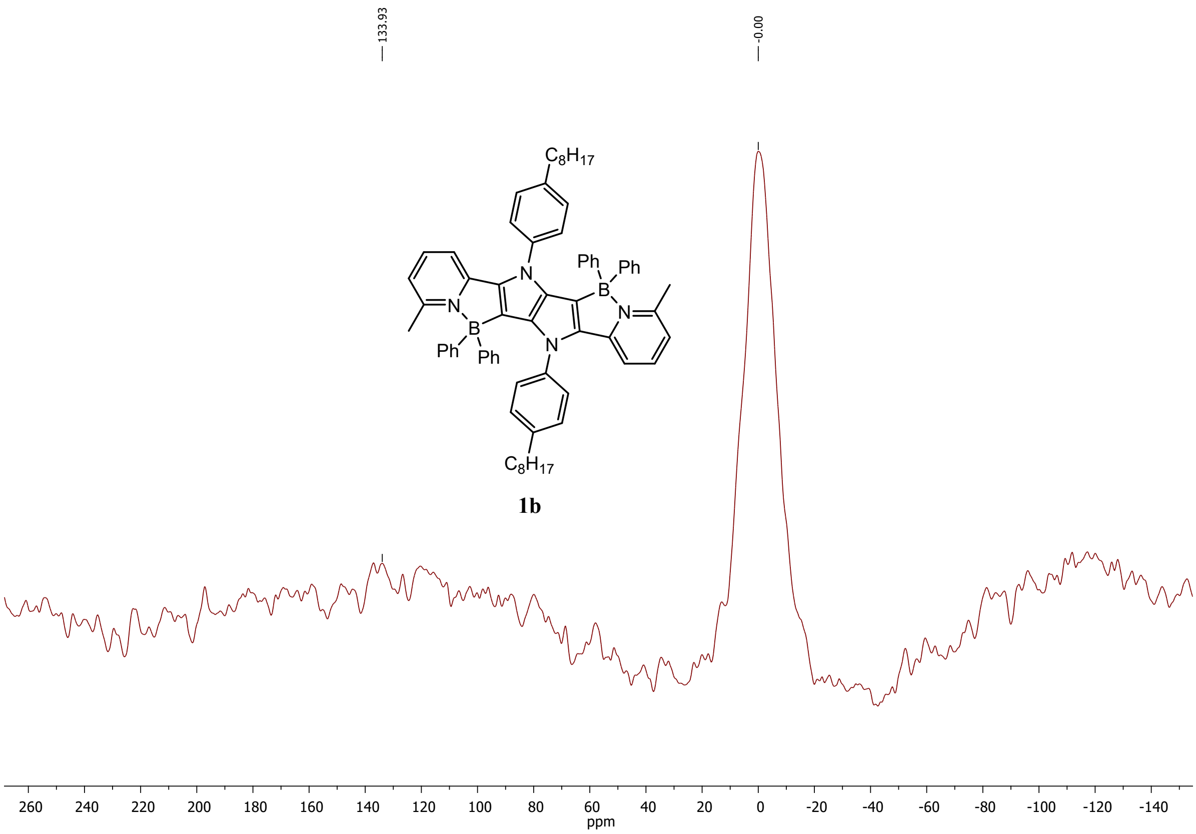
133.93
-0.00
C8H17
Ph
Ph
B
N
N
N
B
Ph
Ph
C8H17
260 240 220 200 180 160 140 120 100 80 60 40 20 0 -20 -40 -60 -80 -100 -120 -140
ppm

1b

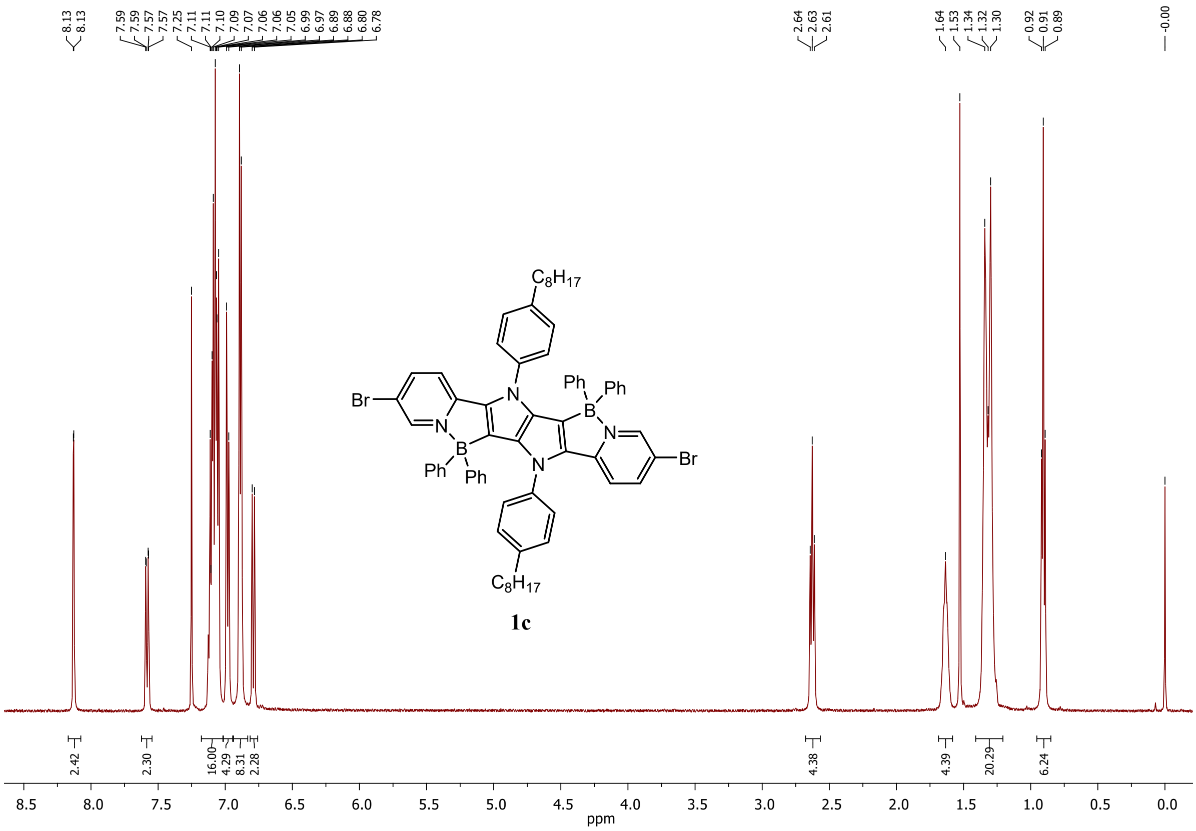
8.13
8.13
7.59
7.59
7.57
7.57
7.25
7.11
7.11
7.10
7.09
7.07
7.06
7.06
7.05
6.99
6.97
6.89
6.88
6.80
6.78
2.64
2.63
2.61
1.64
1.53
1.34
1.32
1.30
0.92
0.91
0.89
-0.00
C8H17
Br
Ph
Ph
B
N
N
N
B
Ph
Ph
Br
C8H17
2.42
2.30
16.00
4.29
8.31
2.28
4.38
4.39
20.29
6.24
8.5 8.0 7.5 7.0 6.5 6.0 5.5 5.0 4.5 4.0 3.5 3.0 2.5 2.0 1.5 1.0 0.5 0.0
ppm

1c

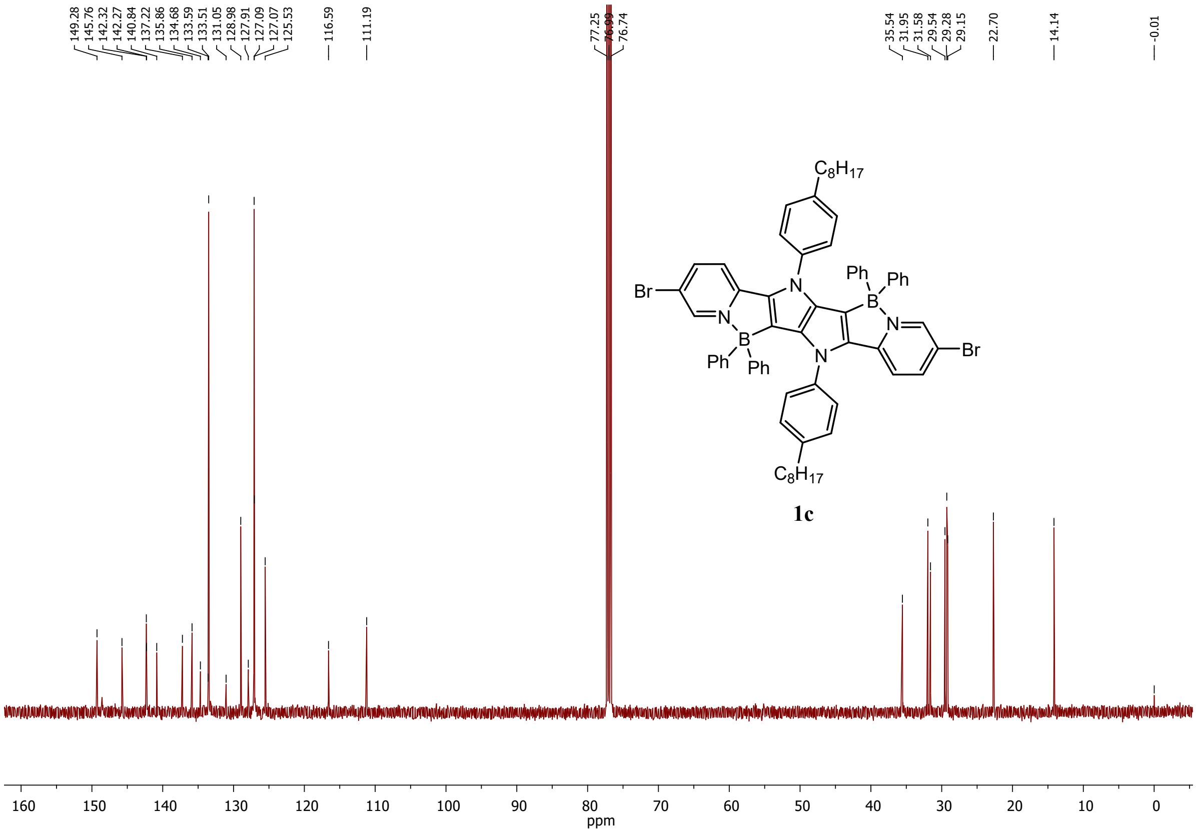
149.28
145.76
142.32
142.27
140.84
137.22
135.86
134.68
133.59
133.51
131.05
128.98
127.91
127.09
127.07
125.53
116.59
111.19
77.25
76.99
76.74
35.54
31.95
31.58
29.54
29.28
29.15
22.70
14.14
-0.01
C8H17
Br
N
Ph
Ph
B
N
Br
N
B
Ph
Ph
N
C8H17
1c
ppm

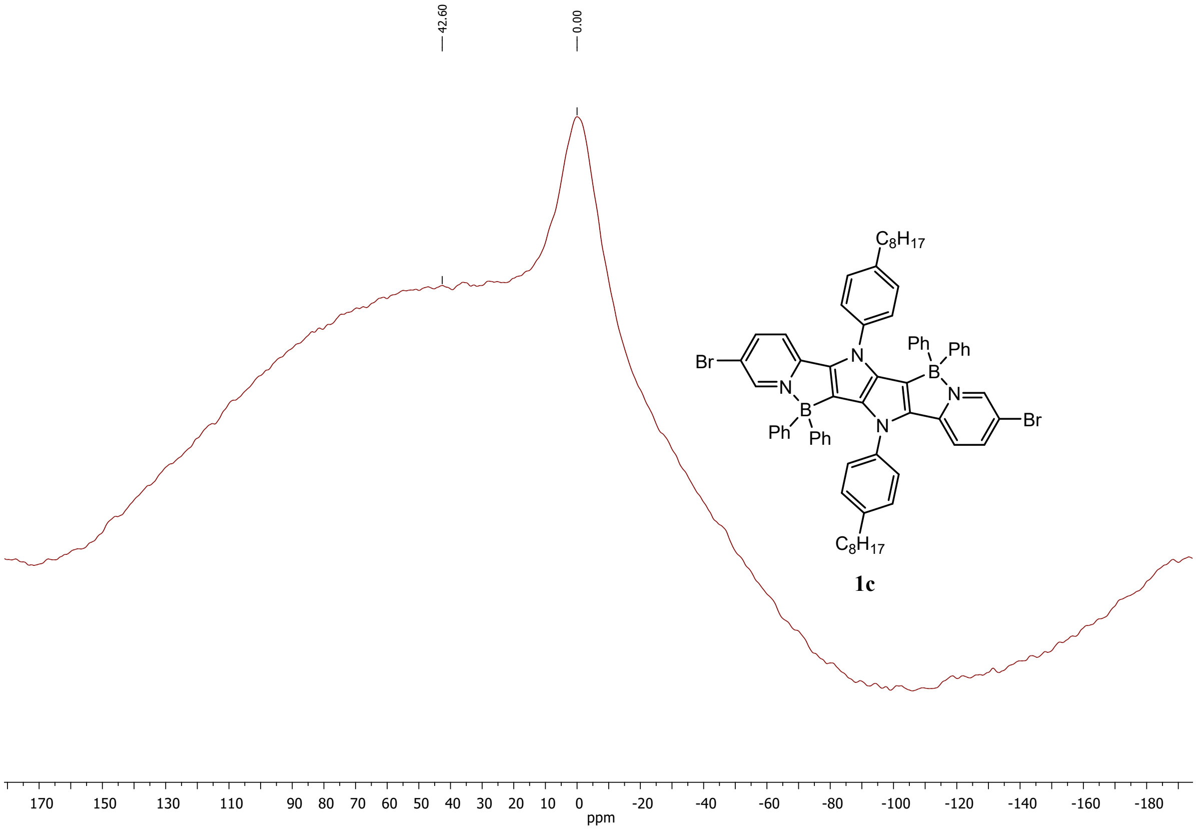
42.60
0.00
C8H17
Br
N
Ph
Ph
B
N
Br
N
B
Ph
Ph
N
C8H17
1c
ppm

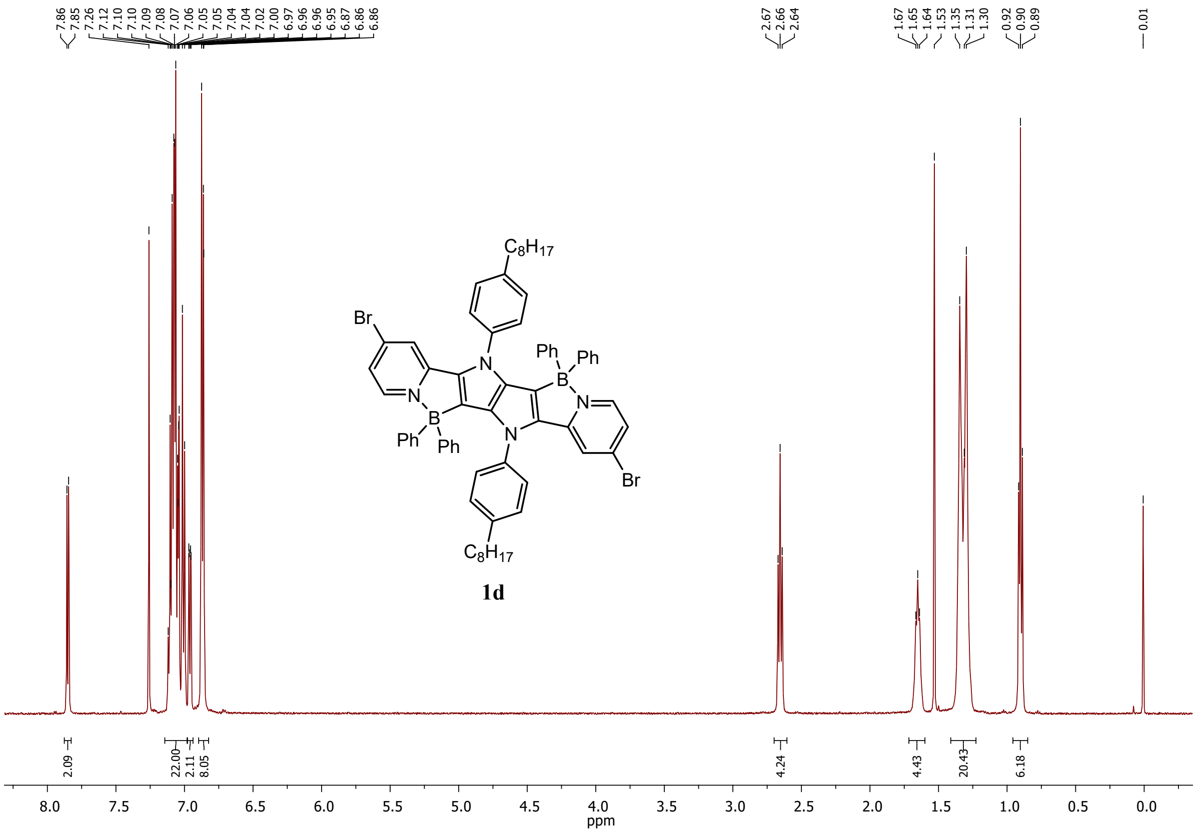
7.86 7.85 7.26 7.12 7.10 7.10 7.09 7.08 7.07 7.06 7.05 7.05 7.04 7.04 7.02 7.00 6.97 6.96 6.96 6.95 6.87 6.86 6.86
2.67 2.66 2.64
1.67 1.65 1.64 1.53 1.35 1.31 1.30 0.92 0.90 0.89
0.01
Br
C8H17
Ph
N
B
1d
2.09
22.00
2.11
8.05
4.24
4.43
20.43
6.18
ppm

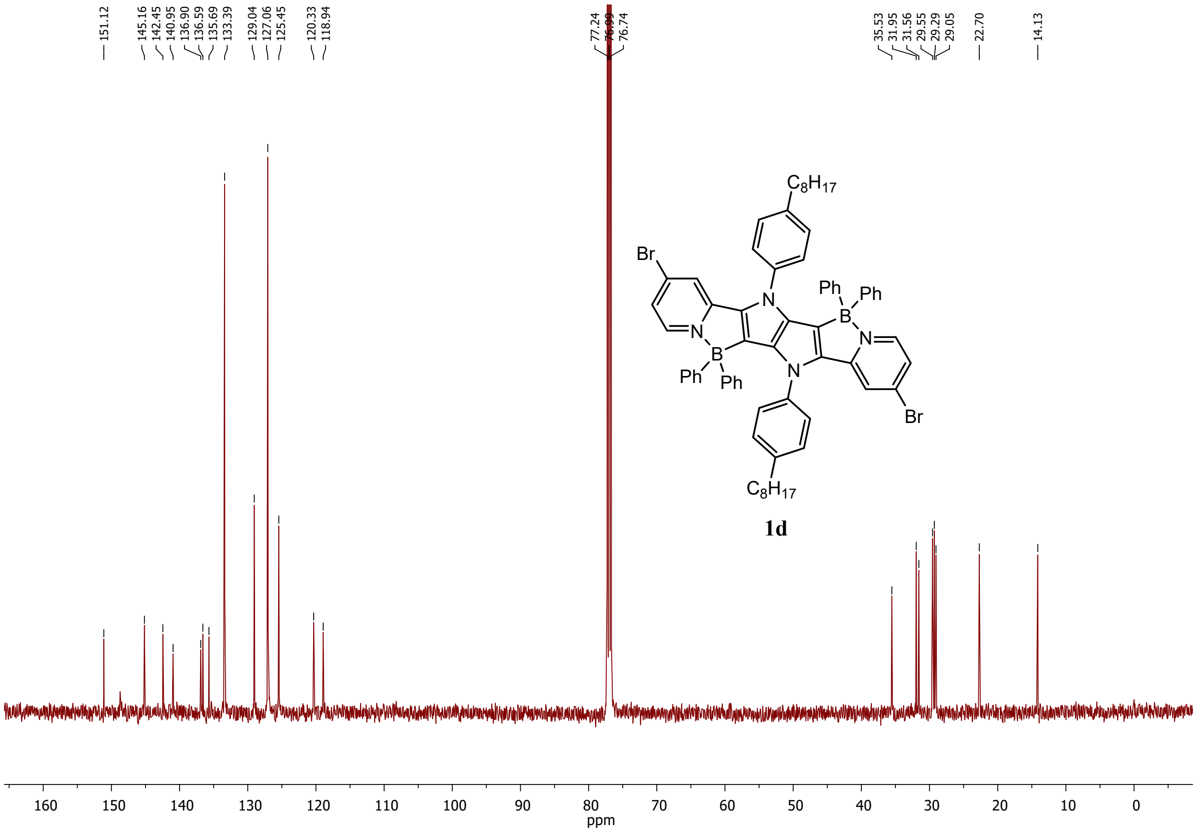
151.12
145.16 142.45 140.95 136.90 136.59 135.69 133.39
129.04 127.06 125.45
120.33 118.94
77.24 76.99 76.74
35.53 31.95 31.56 29.55 29.29 29.05
22.70
14.13
1d
ppm

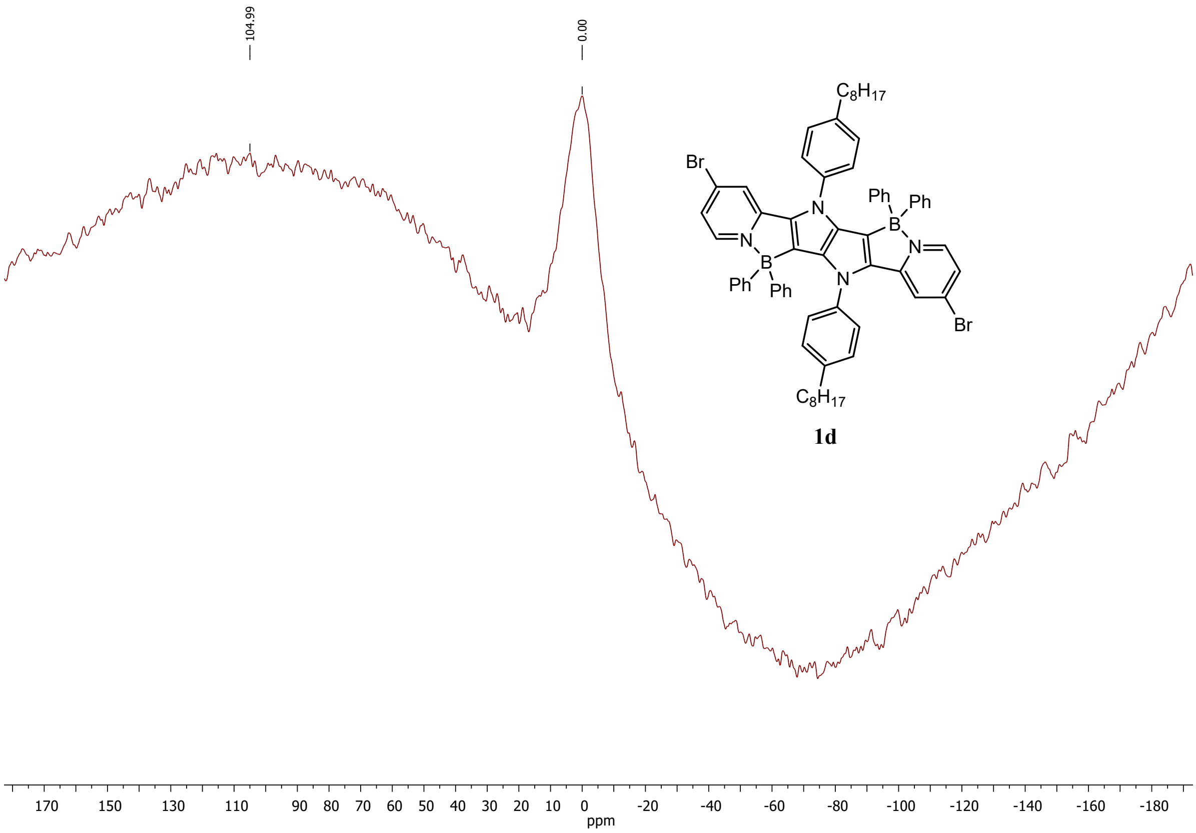
104.99
0.00
Br
Ph
B
N
C8H17
1d
170 150 130 110 90 80 70 60 50 40 30 20 10 0 -20 -40 -60 -80 -100 -120 -140 -160 -180
ppm

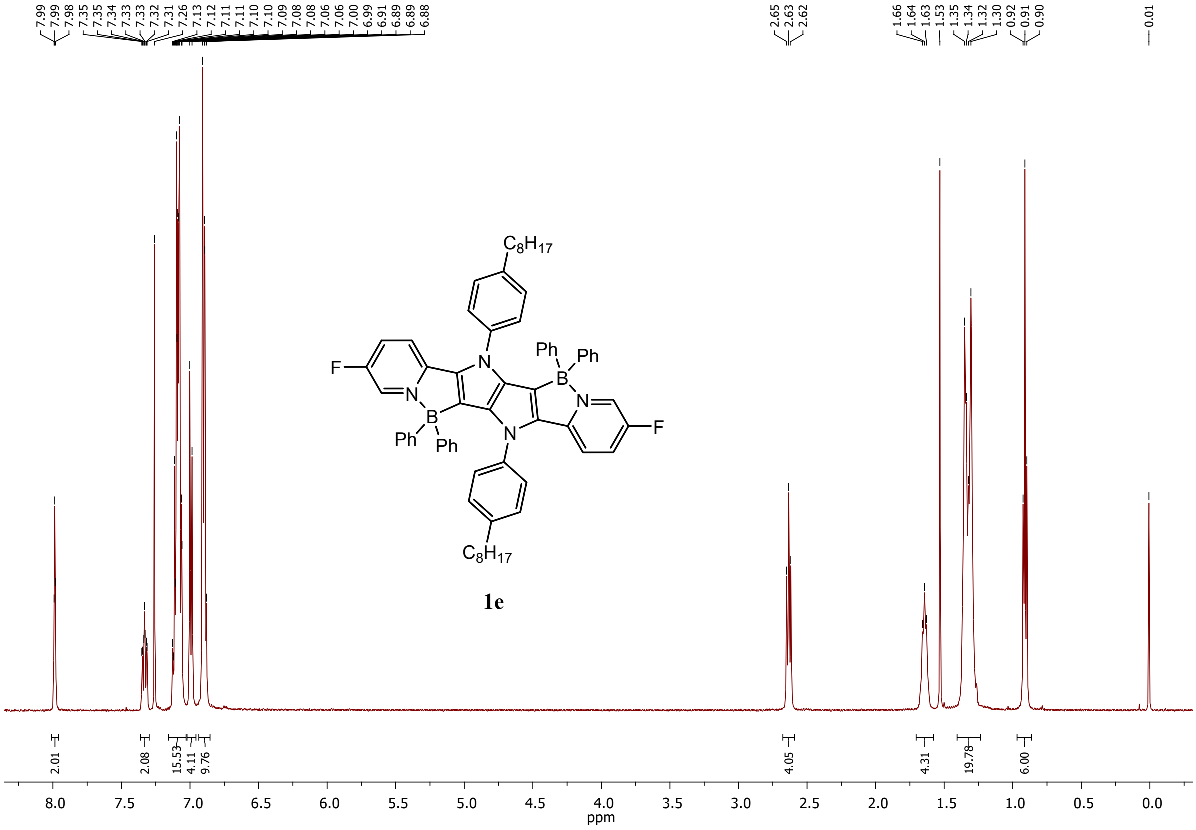
7.99 7.99 7.98 7.35 7.35 7.34 7.33 7.33 7.32 7.31 7.26 7.13 7.12 7.11 7.11 7.10 7.09 7.08 7.08 7.06 7.00 6.99 6.91 6.89 6.89 6.88
2.65 2.63 2.62
1.66 1.64 1.63 1.53 1.35 1.34 1.32 1.30 0.92 0.91 0.90
0.01
F
Ph
B
N
C8H17
1e
2.01 2.08 15.53 4.11 9.76 4.05 4.31 19.78 6.00
8.0 7.5 7.0 6.5 6.0 5.5 5.0 4.5 4.0 3.5 3.0 2.5 2.0 1.5 1.0 0.5 0.0
ppm

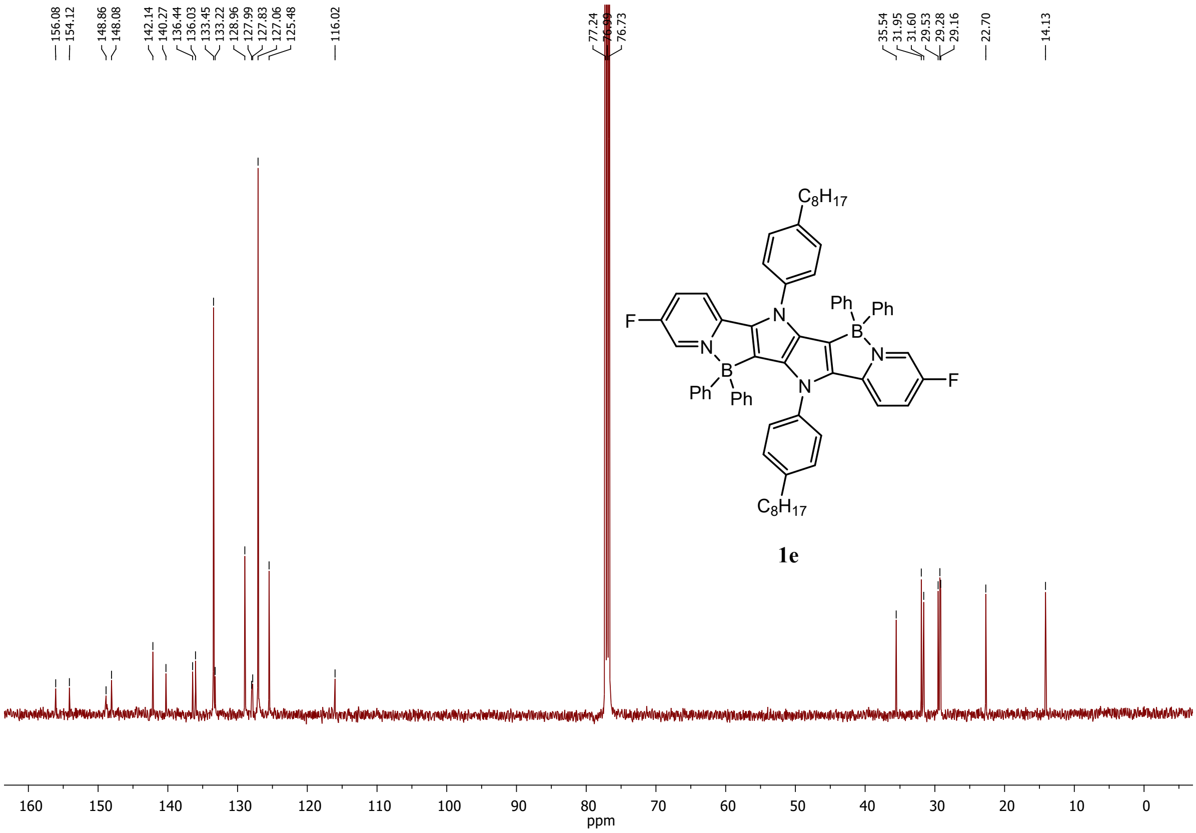
156.08
154.12
148.86
148.08
142.14
140.27
136.44
136.03
133.45
133.22
128.96
127.99
127.83
127.06
125.48
116.02
77.24
76.73
35.54
31.95
31.60
29.53
29.28
29.16
22.70
14.13
C8H17
F
N
Ph
B
1e
160 150 140 130 120 110 100 90 80 70 60 50 40 30 20 10 0
ppm

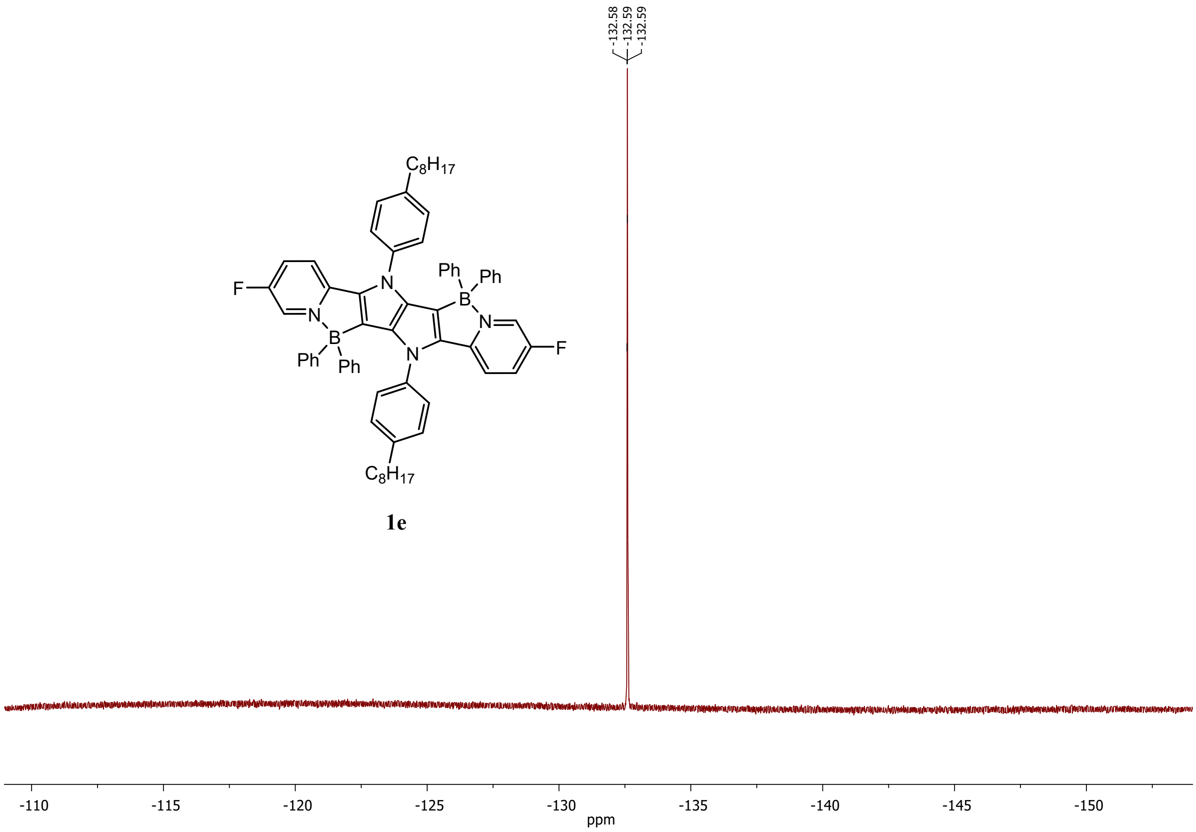
-132.58
-132.59
-132.59
C8H17
F
N
Ph
B
1e
-110 -115 -120 -125 -130 -135 -140 -145 -150
ppm

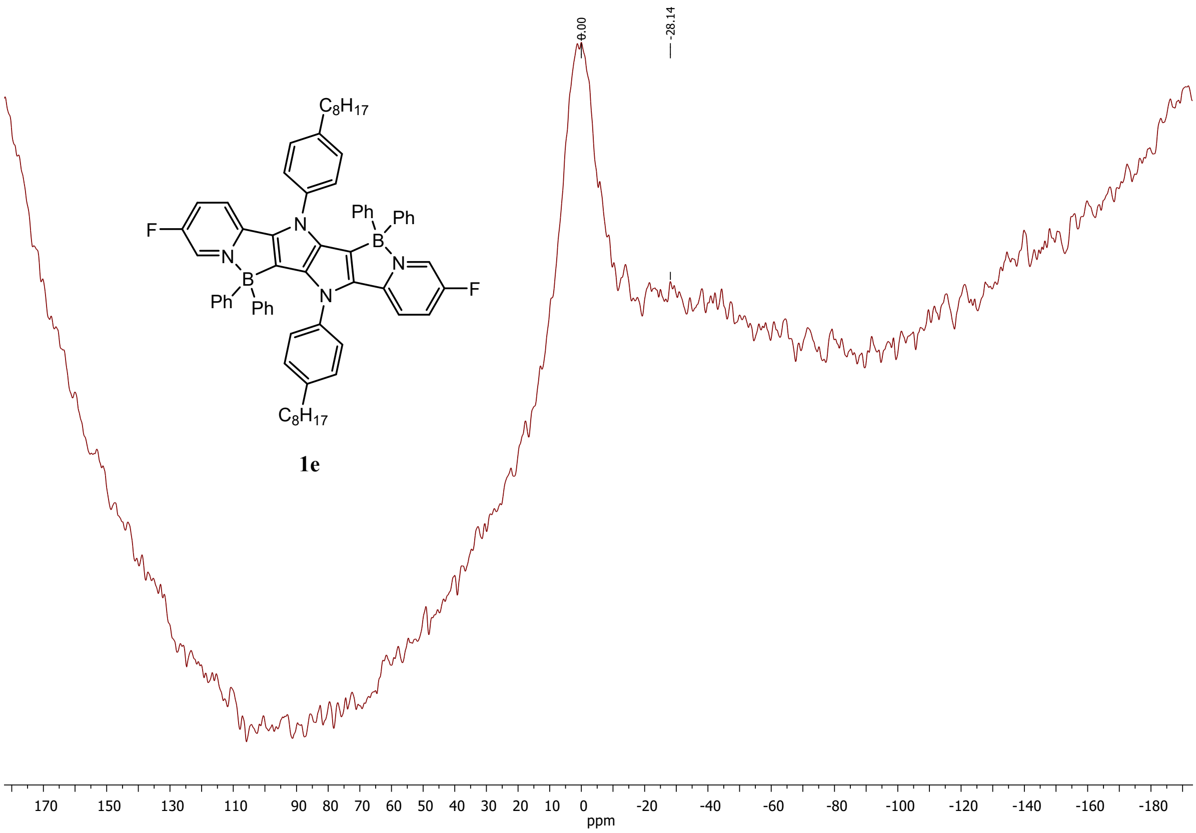
0.00
-28.14
C_8H_{17}
F
N
B
Ph
1e
170 150 130 110 90 80 70 60 50 40 30 20 10 0 -20 -40 -60 -80 -100 -120 -140 -160 -180
ppm

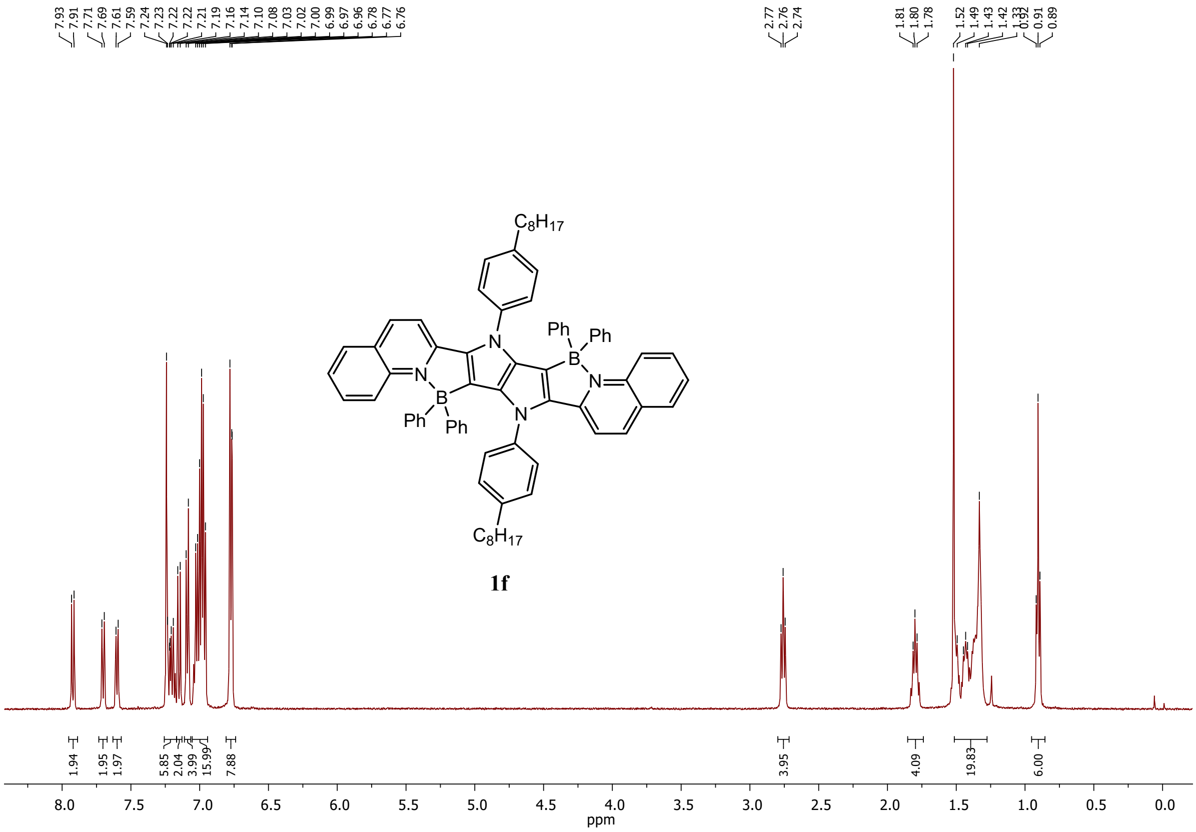
7.93 7.91 7.69 7.71 7.61 7.59 7.24 7.23 7.22 7.22 7.21 7.19 7.16 7.14 7.10 7.08 7.03 7.02 7.00 6.99 6.97 6.96 6.78 6.77 6.76
2.77 2.76 2.74
1.81 1.80 1.78 1.52 1.49 1.43 1.42 1.33 0.92 0.91 0.89
C_8H_{17}
N
B
Ph
1f
1.94 1.95 1.97 5.85 2.04 3.99 15.99 7.88 3.95 4.09 19.83 6.00
8.0 7.5 7.0 6.5 6.0 5.5 5.0 4.5 4.0 3.5 3.0 2.5 2.0 1.5 1.0 0.5 0.0
ppm

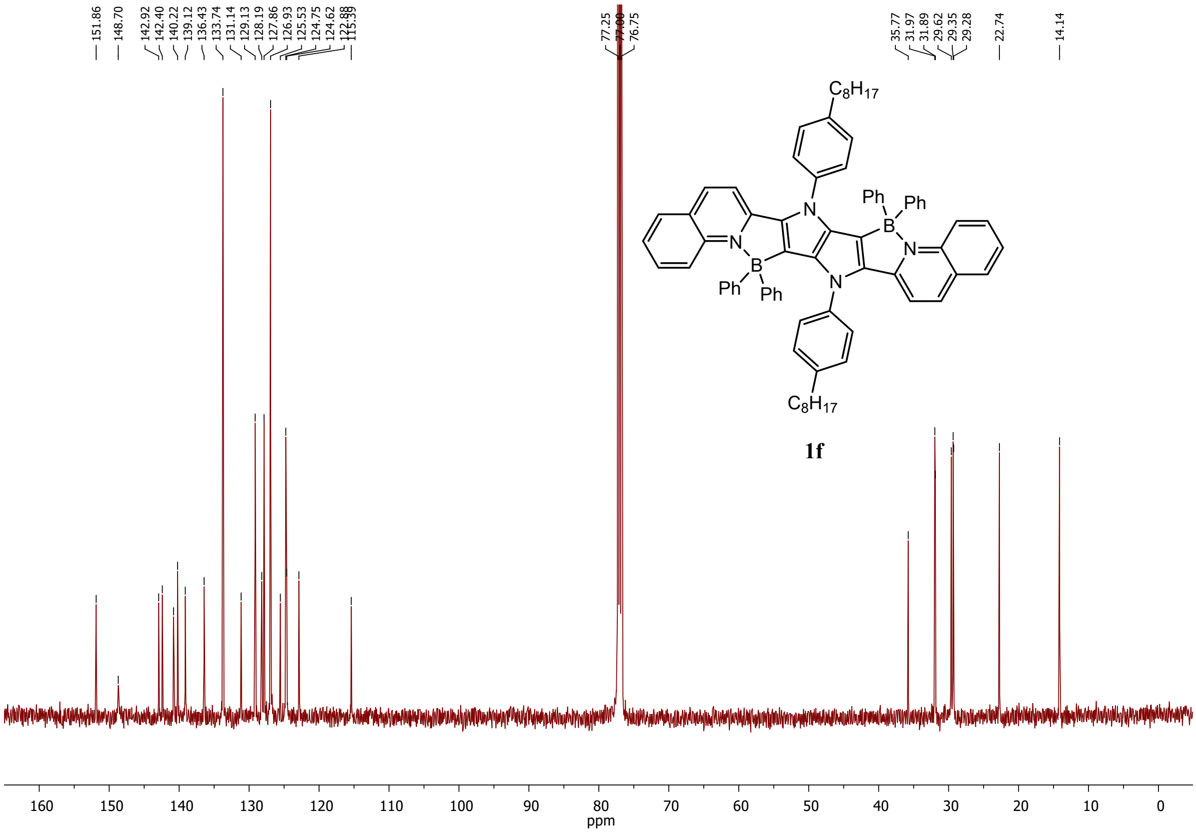
151.86
148.70
142.92
142.40
140.22
139.12
136.43
133.74
131.14
129.13
128.19
127.86
126.93
125.53
124.75
124.62
77.25
77.00
76.75
35.77
31.97
31.89
29.62
29.35
29.28
22.74
14.14
C_8H_{17}
Ph
Ph
B
N
1f
ppm

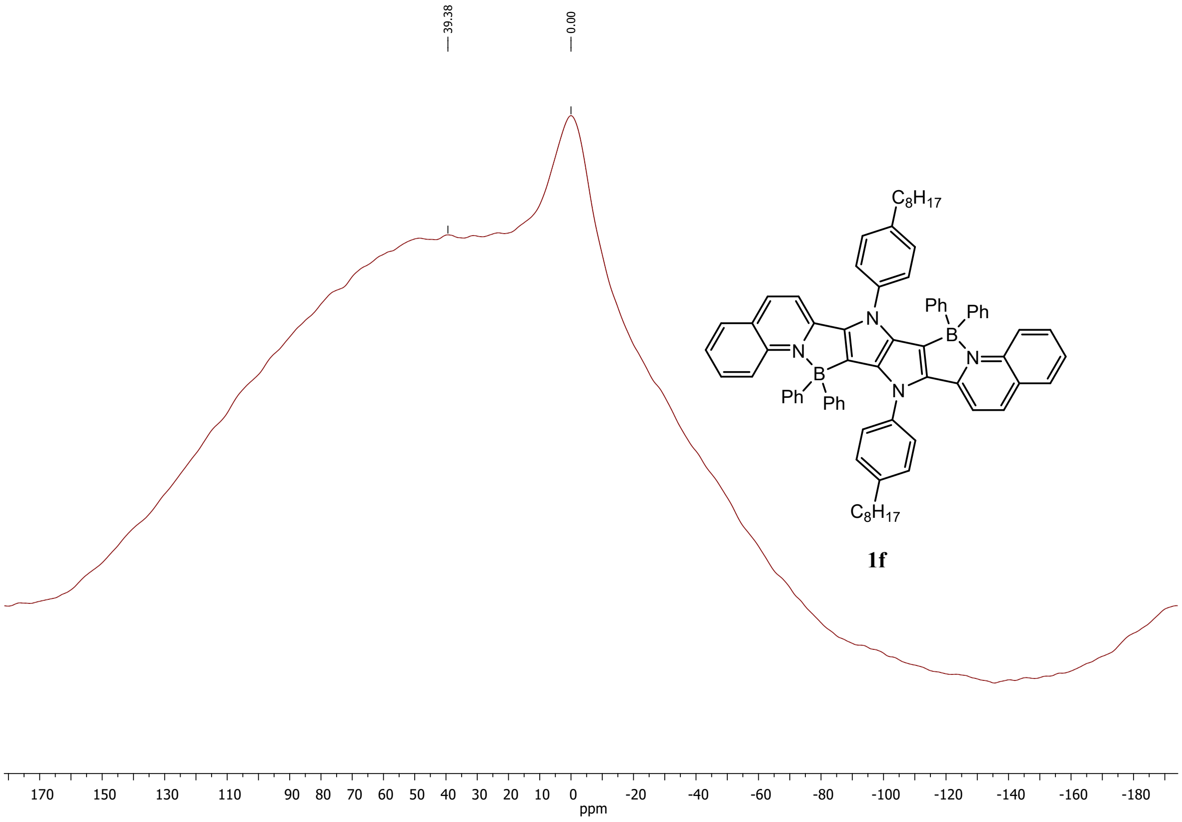
39.38
0.00
C_8H_{17}
Ph
Ph
B
N
1f
ppm

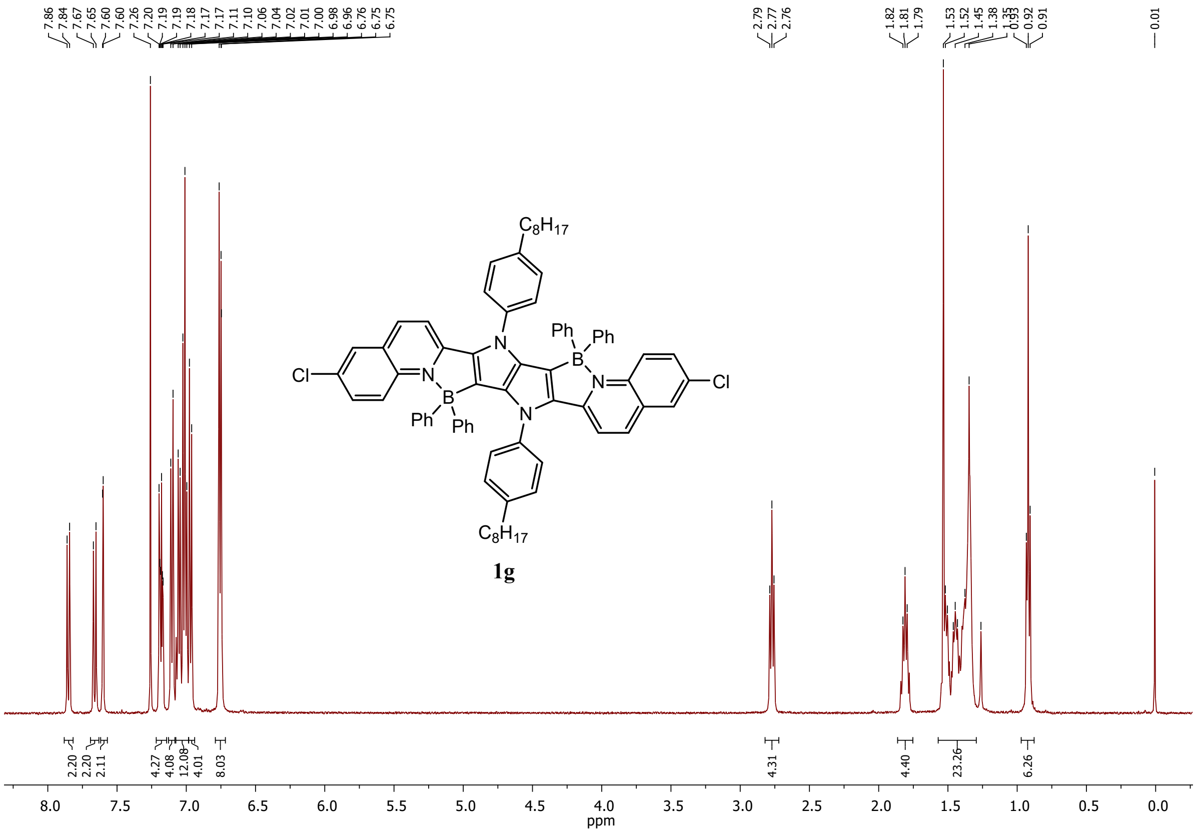
7.86 7.84 7.67 7.65 7.60 7.60 7.26 7.20 7.19 7.19 7.18 7.17 7.17 7.11 7.10 7.06 7.04 7.02 7.01 7.00 6.98 6.96 6.76 6.75 6.75
2.79 2.77 2.76
1.82 1.81 1.79
1.53 1.52 1.45 1.38 1.35 0.93 0.92 0.91
0.01
2.20 2.20 2.11 4.27 4.08 12.08 4.01 8.03 4.31 4.40 23.26 6.26
ppm
C_8H_{17}
Ph
Ph
Cl
N
B
1g

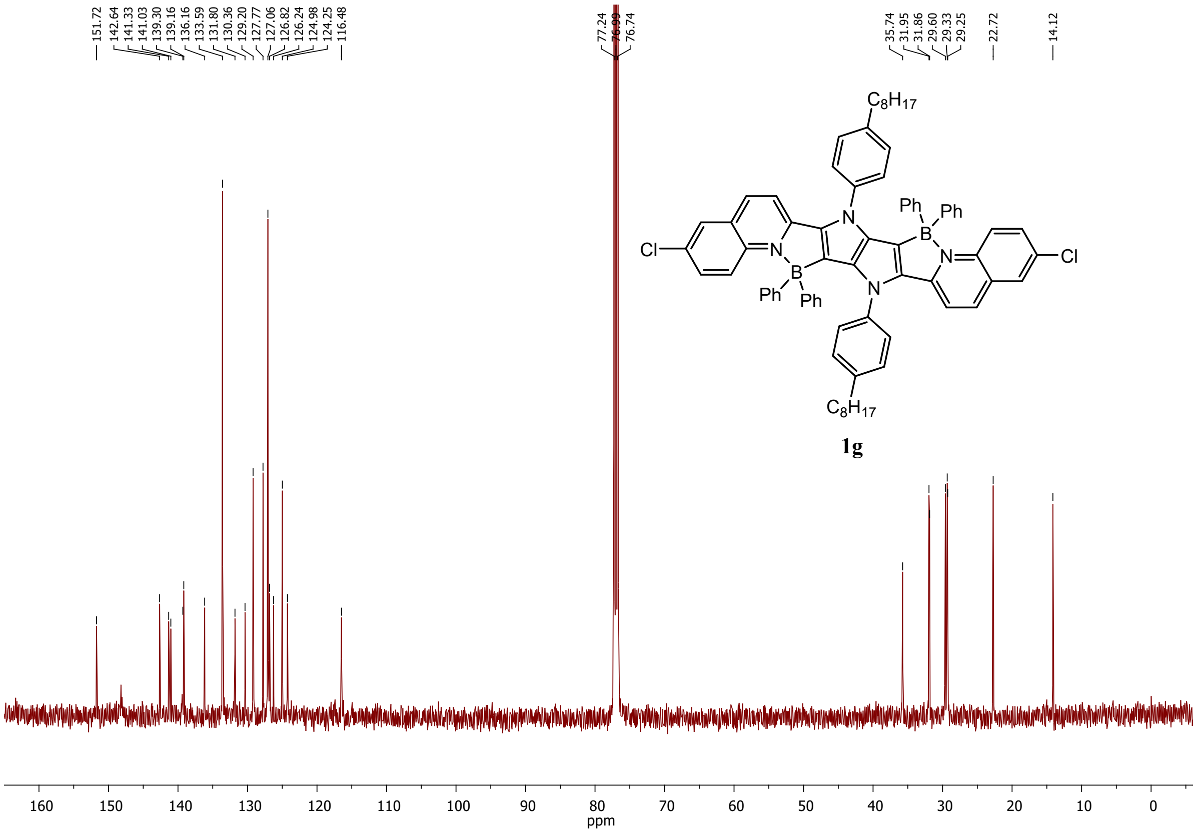
151.72 142.64 141.33 141.03 139.30 139.16 136.16 133.59 131.80 130.36 129.20 127.77 127.06 126.82 126.24 124.98 124.25 116.48
77.24 76.99 76.74
35.74 31.95 31.86 29.60 29.33 29.25 22.72 14.12
ppm
C_8H_{17}
Ph
Ph
Cl
N
B
1g

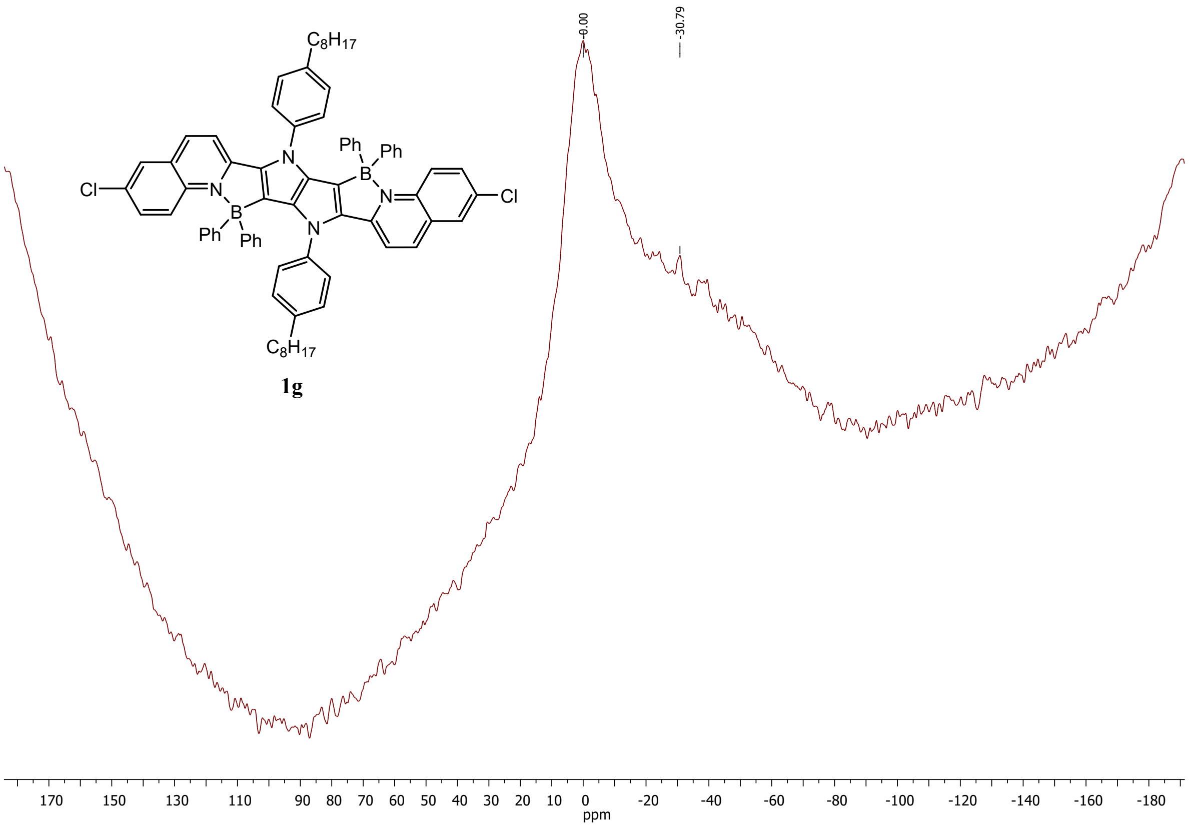

1g

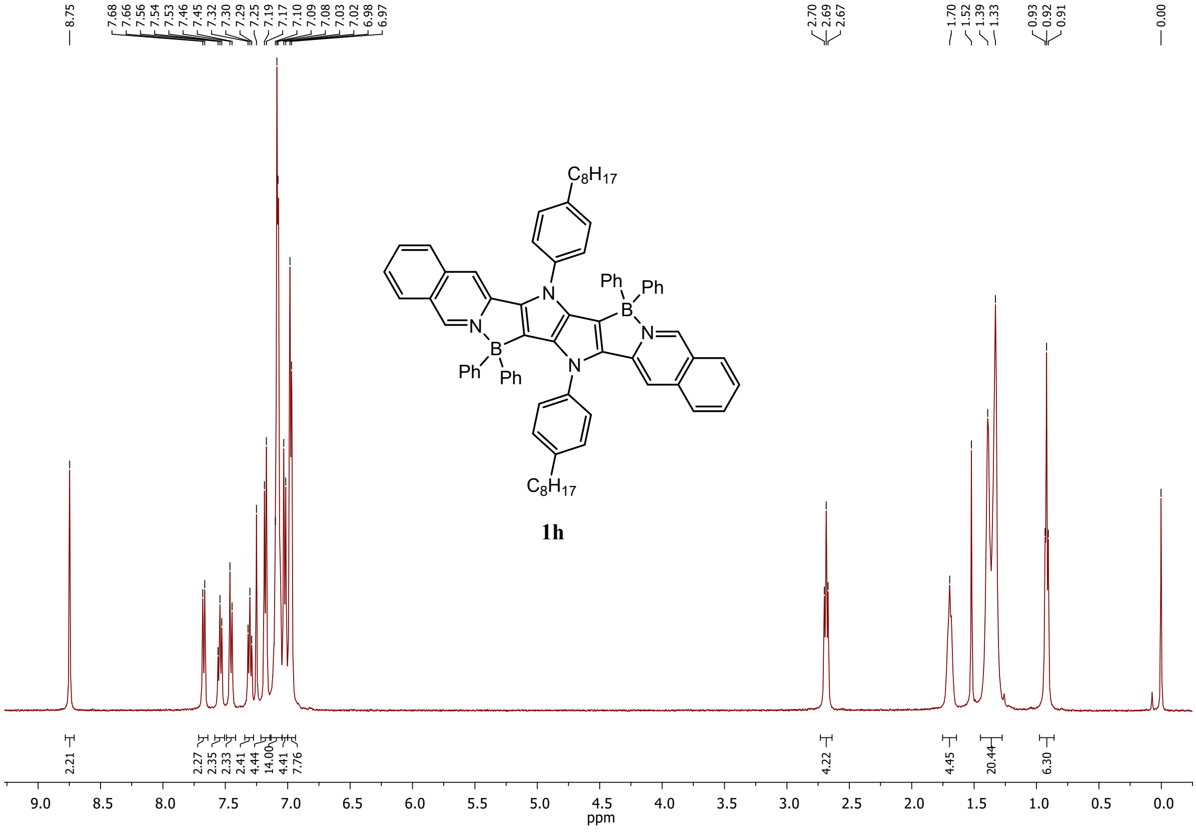

1h

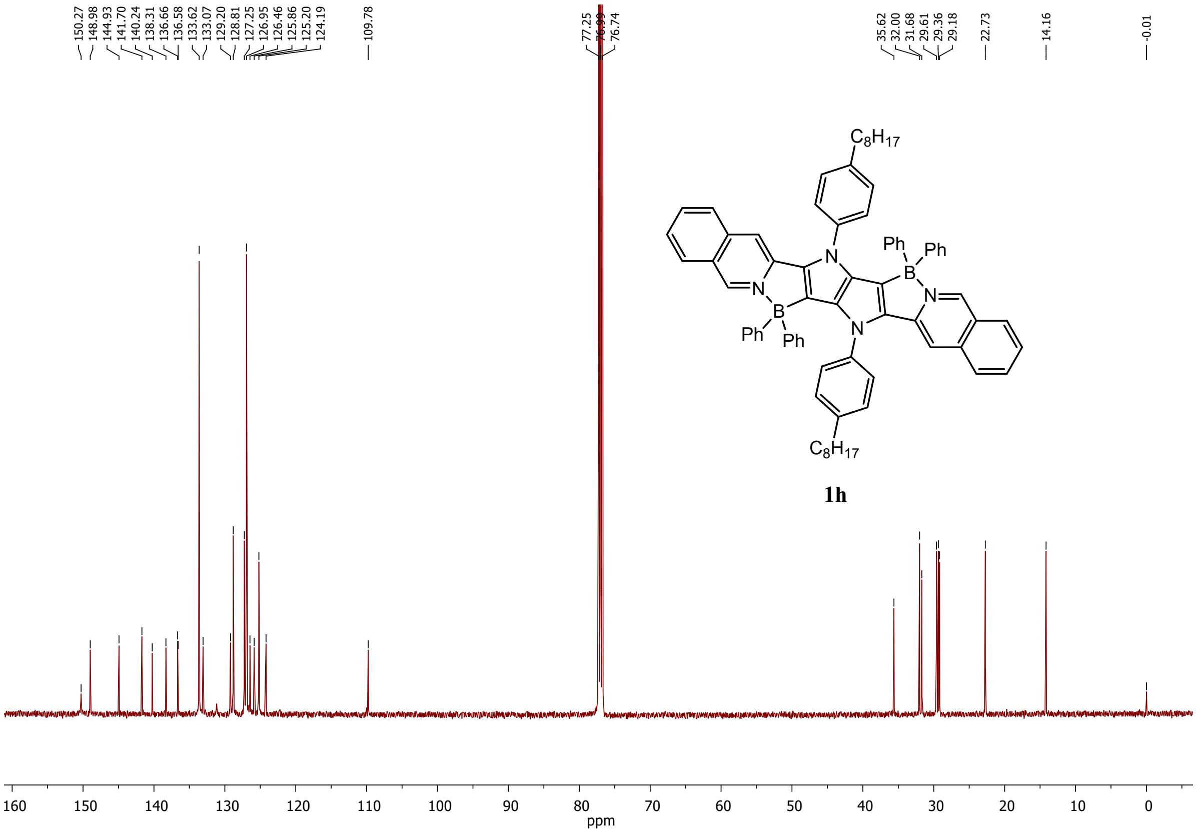
150.27
148.98
144.93
141.70
140.24
138.31
136.66
136.58
133.62
133.07
129.20
128.81
127.25
126.95
126.46
125.86
125.20
124.19
109.78
77.25
76.99
76.74
35.62
32.00
31.68
29.61
29.36
29.18
22.73
14.16
-0.01
C8H17
N
Ph
Ph
B
N
B
Ph
Ph
N
C8H17
1h
ppm

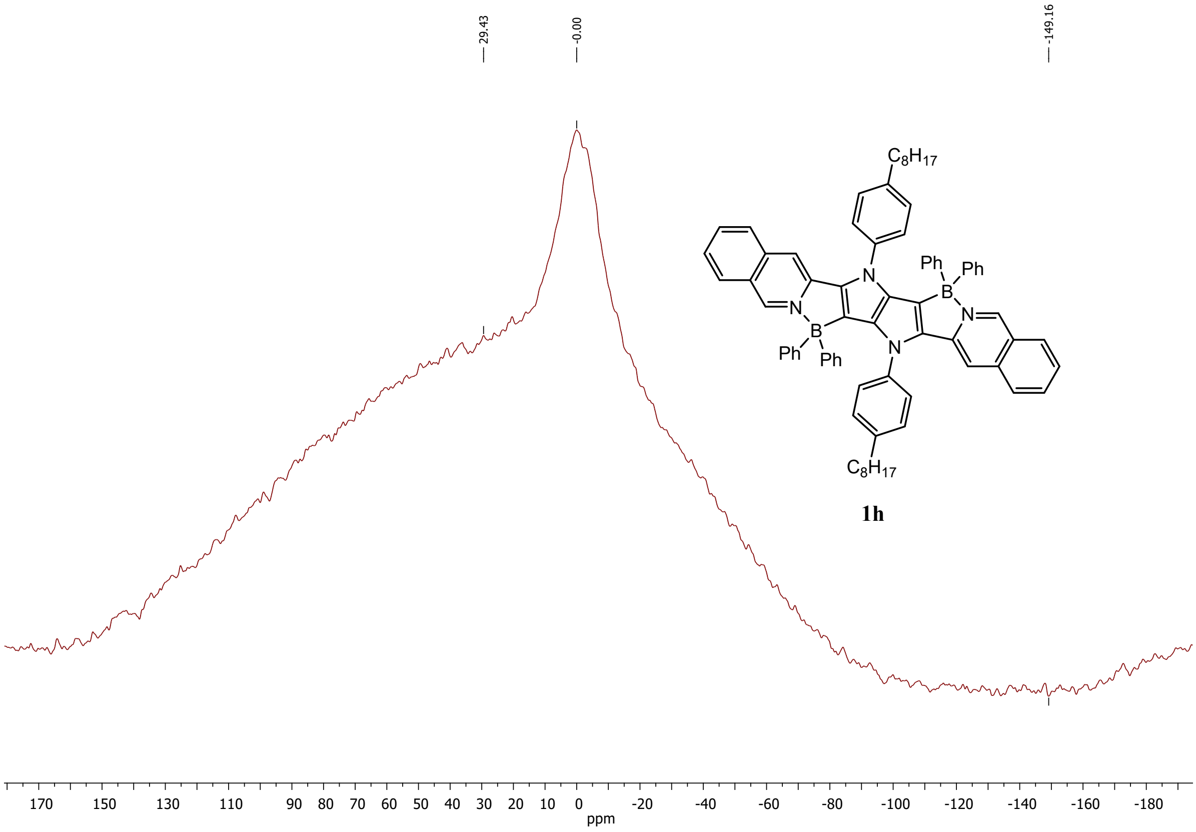
29.43
-0.00
-149.16
C8H17
N
Ph
Ph
B
N
B
Ph
Ph
N
C8H17
1h
ppm

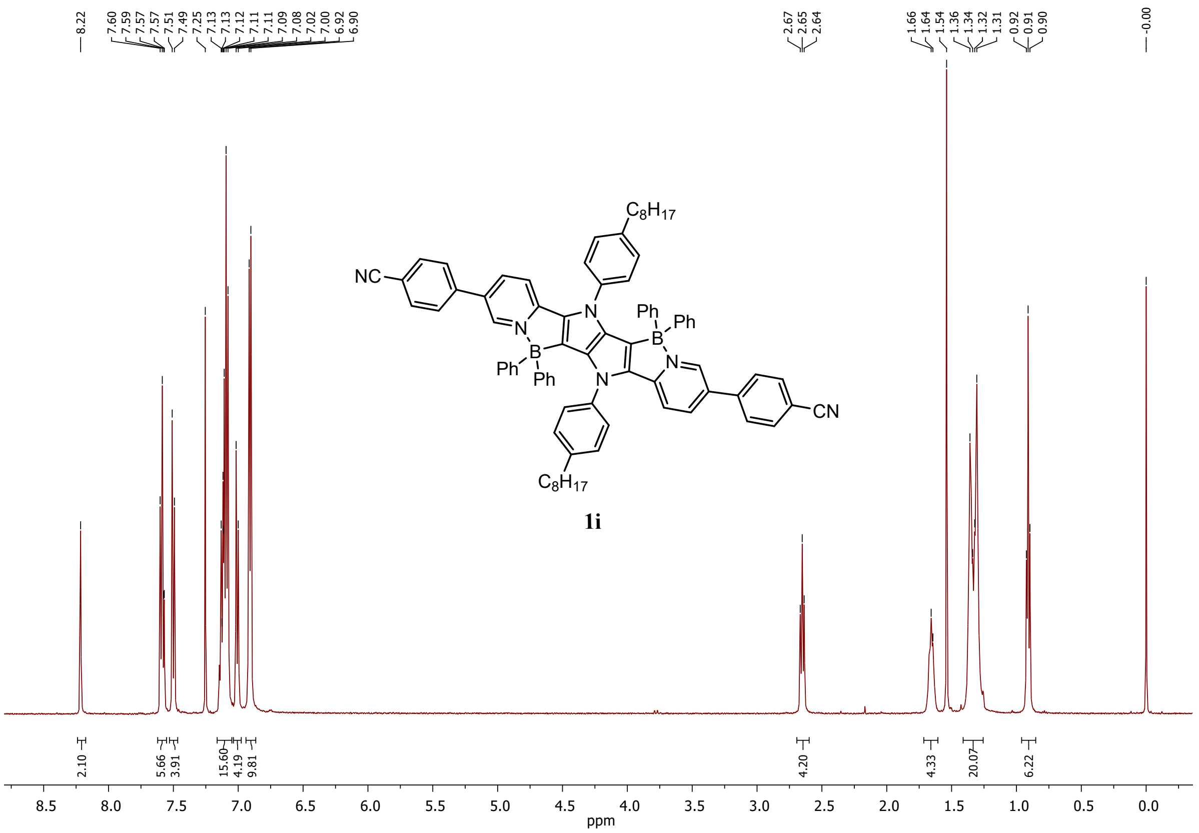

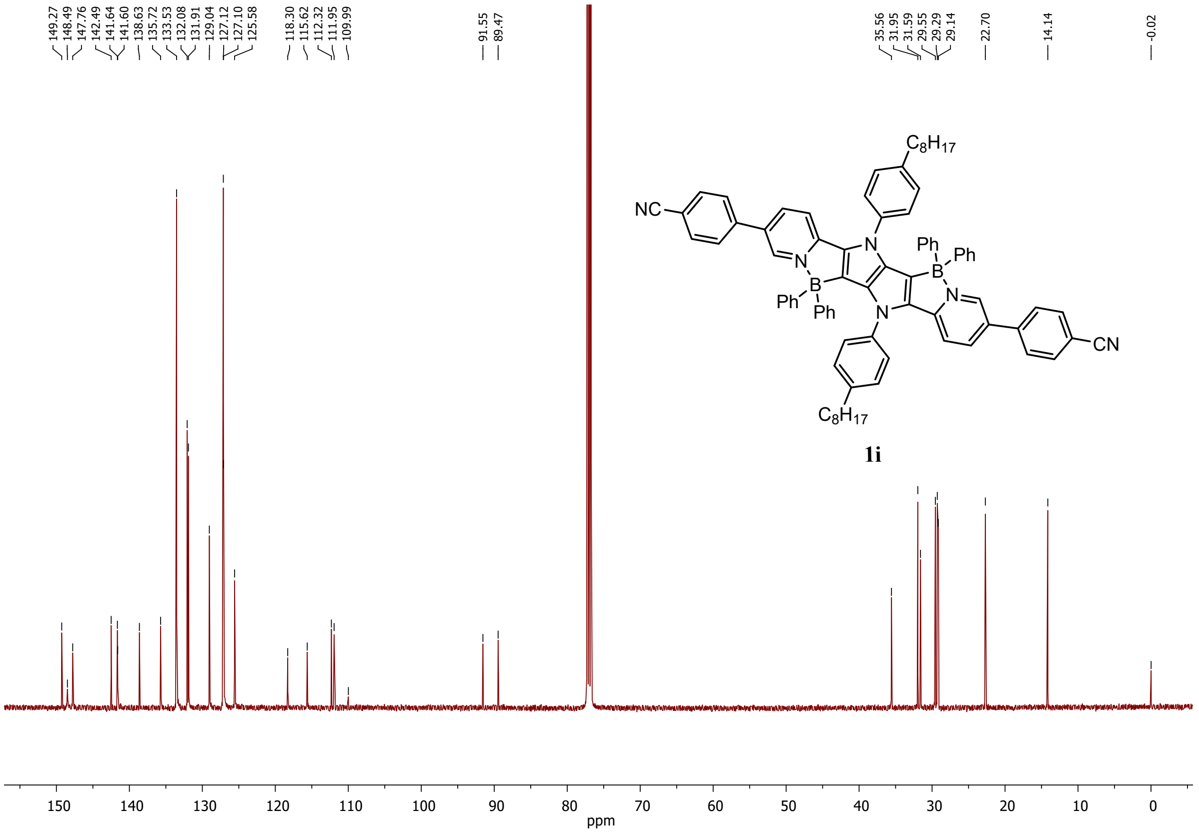

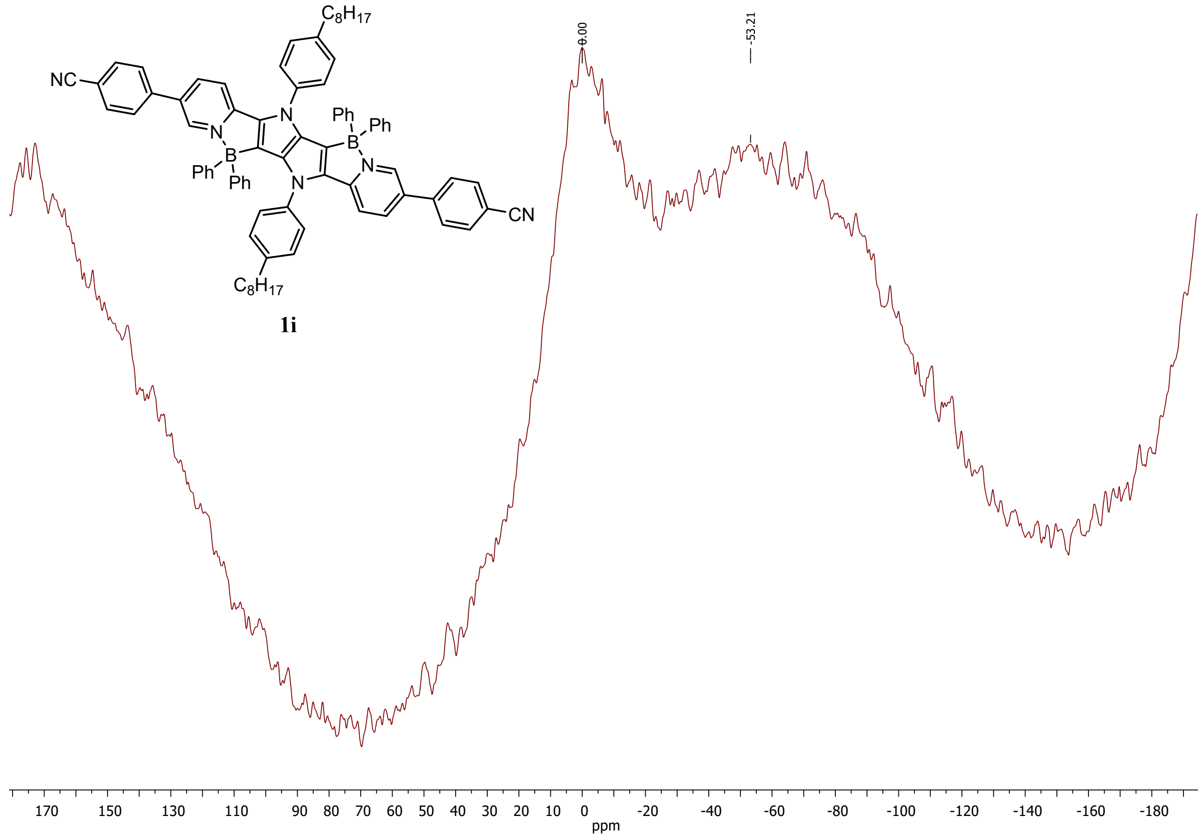

0.00
-53.21
NC
CN
C_8H_{17}
Ph
B
N
1i
170 150 130 110 90 80 70 60 50 40 30 20 10 0 -20 -40 -60 -80 -100 -120 -140 -160 -180
ppm

(1j)

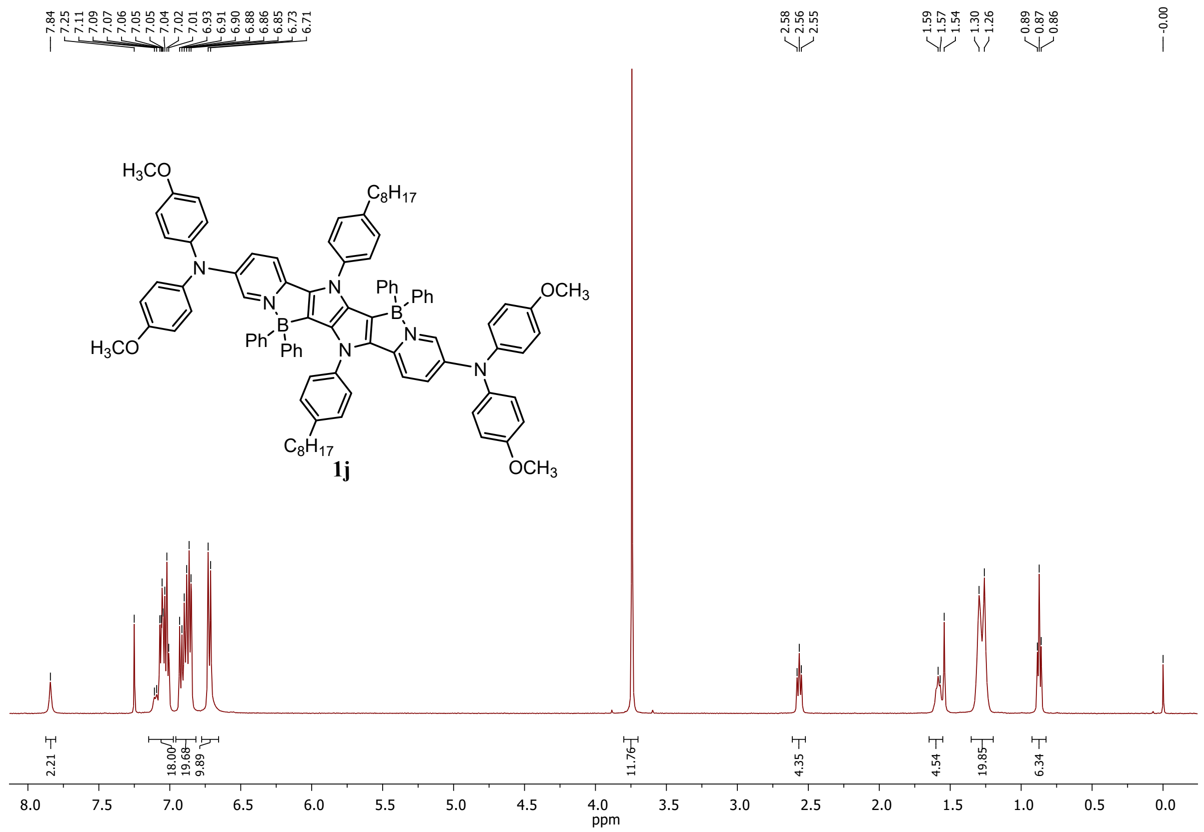

7.84 7.25 7.11 7.09 7.07 7.06 7.05 7.05 7.04 7.02 7.01 6.93 6.91 6.90 6.88 6.86 6.85 6.73 6.71
2.58 2.56 2.55
1.59 1.57 1.54 1.30 1.26
0.89 0.87 0.86
-0.00
H_3CO
OCH_3
C_8H_{17}
Ph
B
N
1j
2.21 18.00 19.68 9.89 11.76 4.35 4.54 19.85 6.34
8.0 7.5 7.0 6.5 6.0 5.5 5.0 4.5 4.0 3.5 3.0 2.5 2.0 1.5 1.0 0.5 0.0
ppm

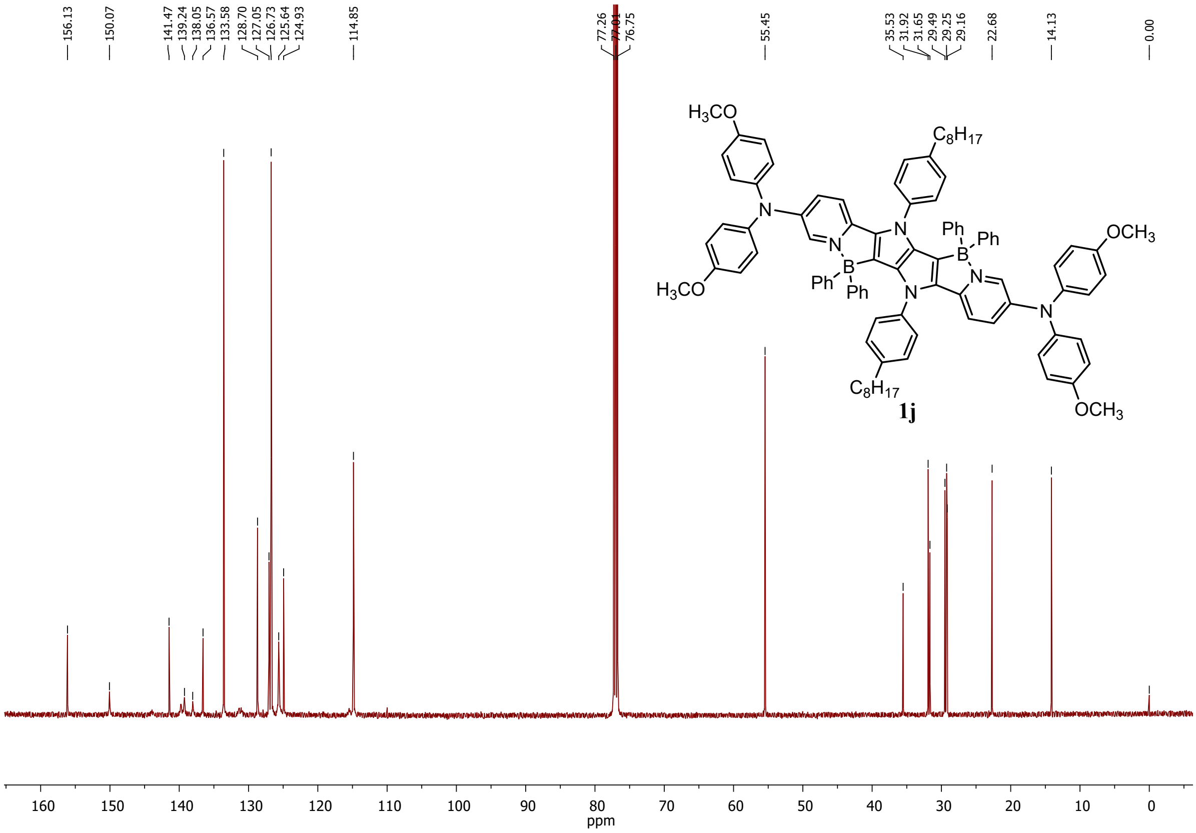
156.13
150.07
141.47
139.24
138.05
136.57
133.58
128.70
127.05
126.73
125.64
124.93
114.85
77.26
77.01
76.75
55.45
35.53
31.92
31.65
29.49
29.25
29.16
22.68
14.13
0.00
H_3CO
C_8H_{17}
N
Ph
B
OCH_3
1j
160 150 140 130 120 110 100 90 80 70 60 50 40 30 20 10 0
ppm

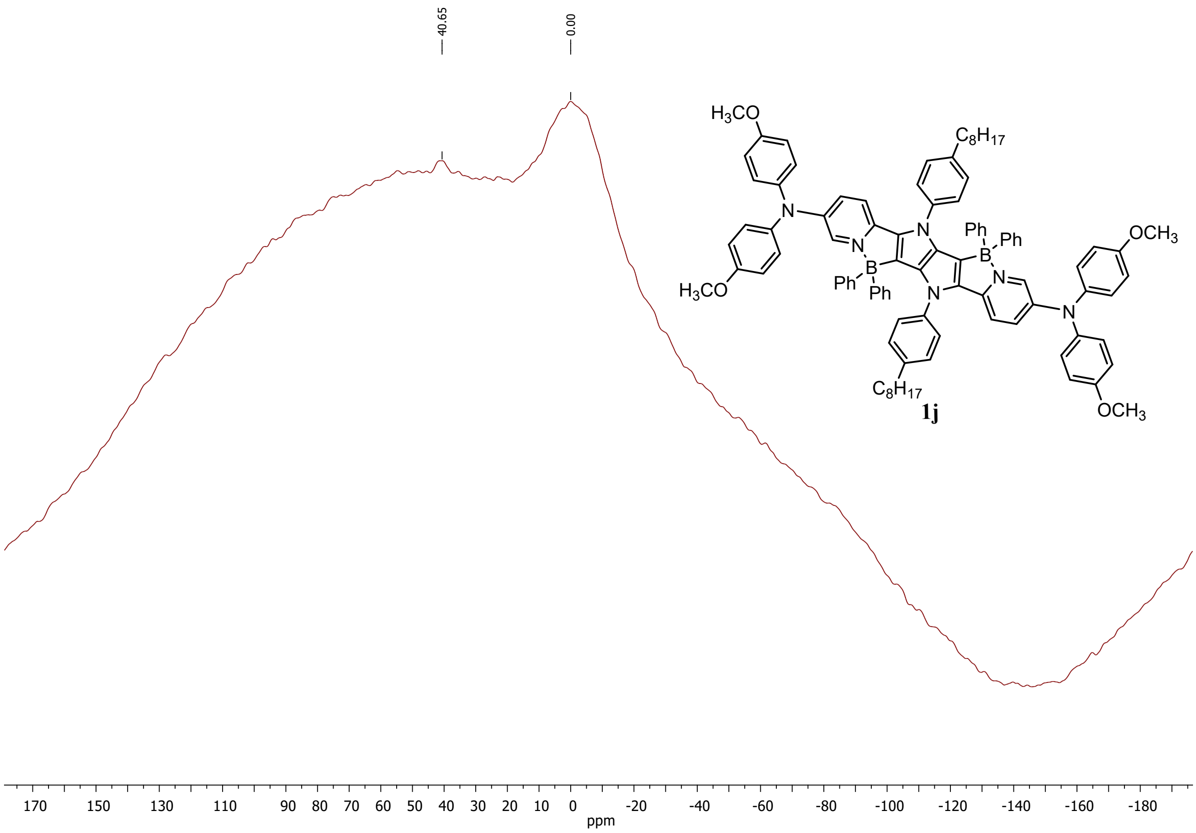
40.65
0.00
H_3CO
C_8H_{17}
N
Ph
B
OCH_3
1j
170 150 130 110 90 80 70 60 50 40 30 20 10 0 -20 -40 -60 -80 -100 -120 -140 -160 -180
ppm

(1k)

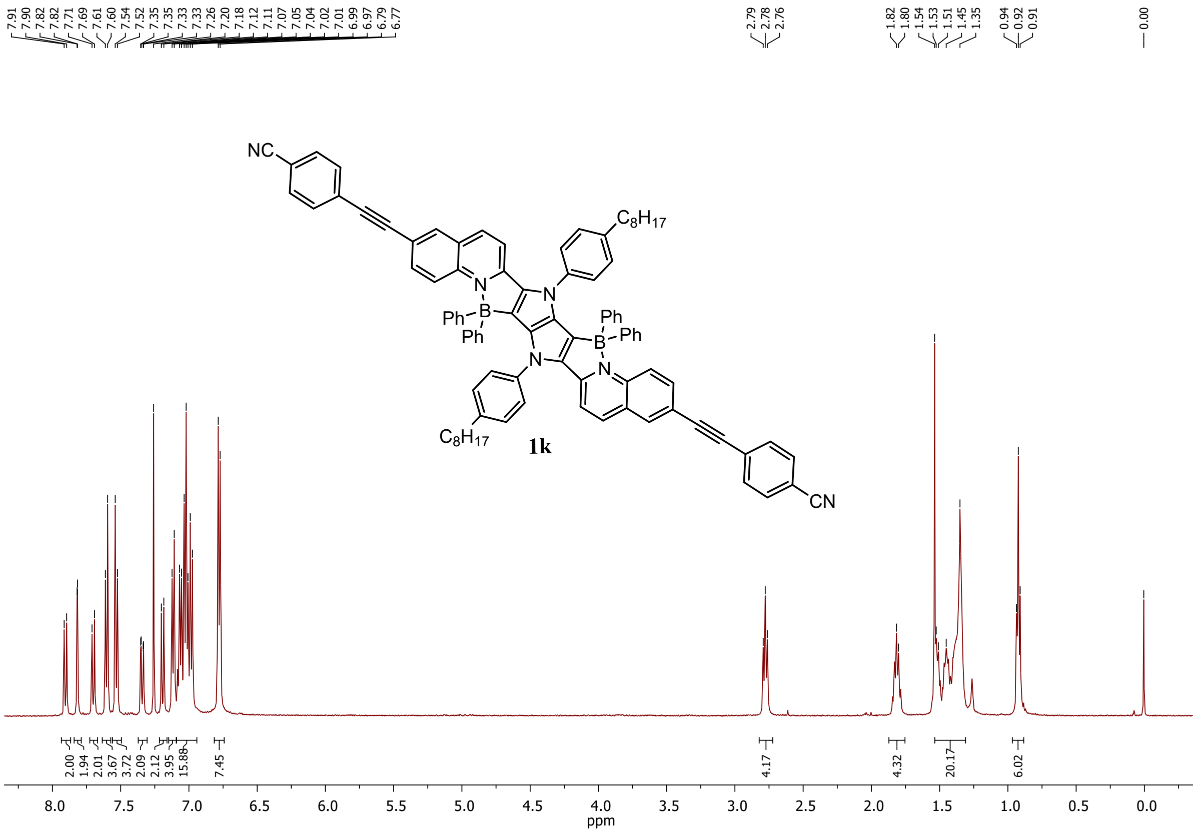
7.91
7.90
7.82
7.82
7.71
7.69
7.61
7.60
7.54
7.52
7.35
7.35
7.33
7.33
7.26
7.20
7.18
7.12
7.11
7.07
7.05
7.04
7.02
7.01
6.99
6.97
6.79
6.77
2.79
2.78
2.76
1.82
1.80
1.54
1.53
1.51
1.45
1.35
0.94
0.92
0.91
0.00
NC
C_8H_{17}
N
N
B
Ph
Ph
Ph
Ph
CN
1k
2.00
1.94
2.01
3.67
3.72
2.09
2.12
3.95
15.88
7.45
4.17
4.32
20.17
6.02
8.0 7.5 7.0 6.5 6.0 5.5 5.0 4.5 4.0 3.5 3.0 2.5 2.0 1.5 1.0 0.5 0.0
ppm

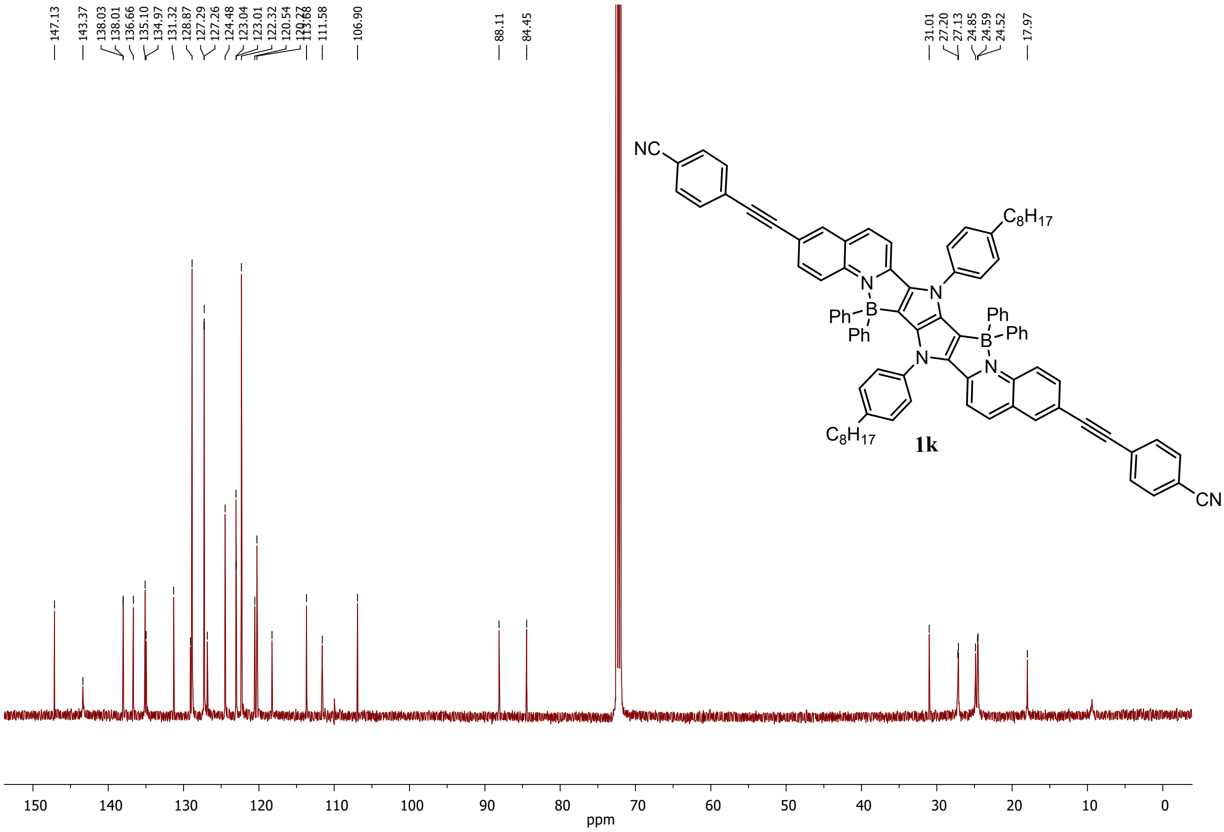
147.13
143.37
138.03
138.01
136.66
135.10
134.97
131.32
128.87
127.29
127.26
124.48
123.04
123.01
122.32
120.54
120.27
113.68
111.58
106.90
88.11
84.45
31.01
27.20
27.13
24.85
24.59
24.52
17.97
NC
C_8H_{17}
N
N
B
Ph
Ph
Ph
Ph
CN
1k
150 140 130 120 110 100 90 80 70 60 50 40 30 20 10 0
ppm

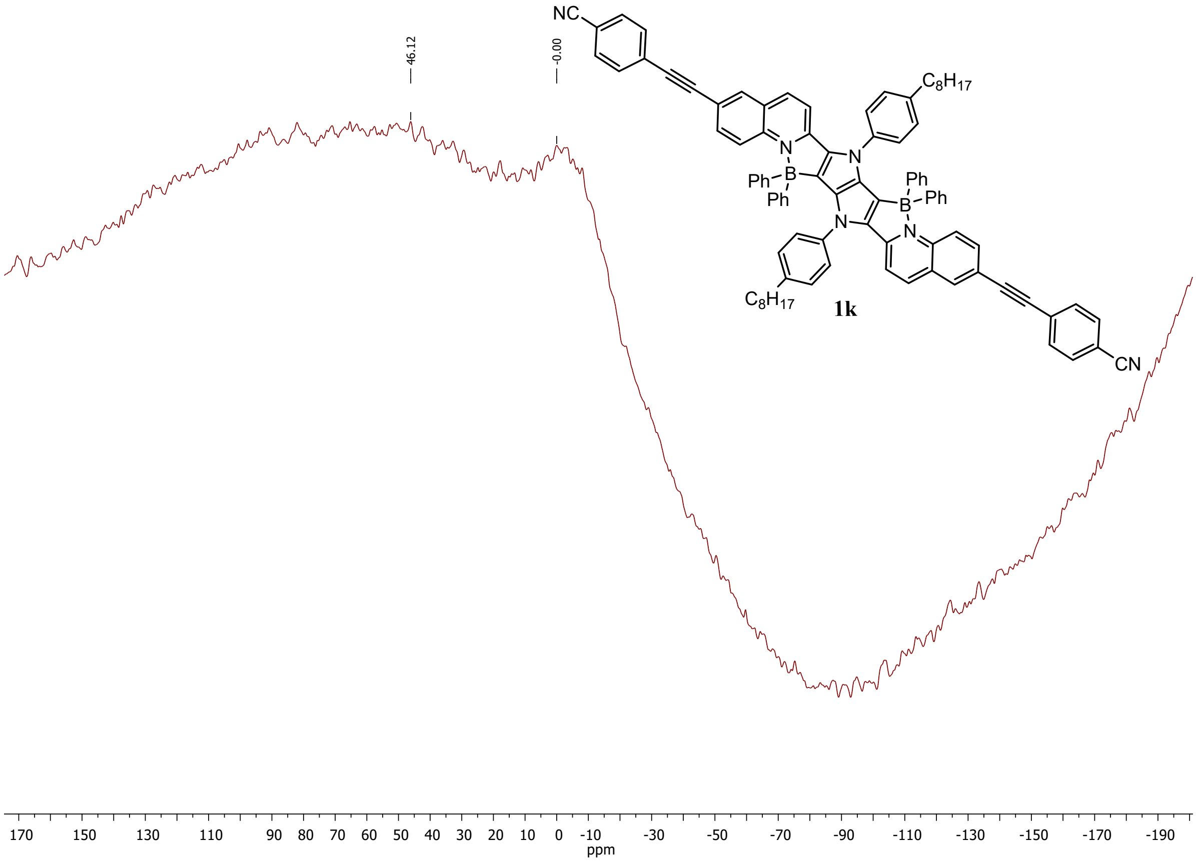
46.12
-0.00
NC
CN
C8H17
Ph
N
B
1k
170 150 130 110 90 80 70 60 50 40 30 20 10 0 -10 -30 -50 -70 -90 -110 -130 -150 -170 -190
ppm

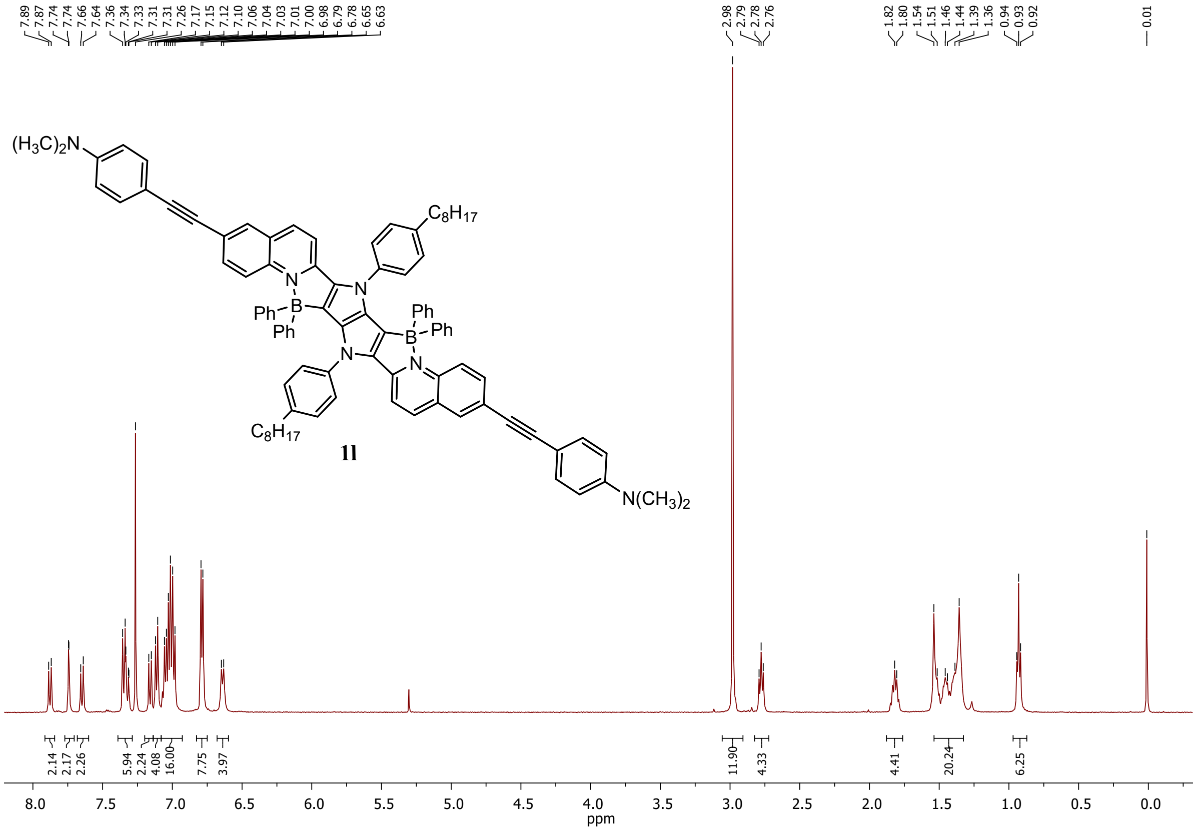
7.89 7.87 7.74 7.74 7.66 7.64 7.36 7.34 7.33 7.31 7.31 7.26 7.17 7.15 7.12 7.10 7.06 7.04 7.03 7.01 7.00 6.98 6.79 6.78 6.65 6.63
2.98 2.79 2.78 2.76
1.82 1.80 1.54 1.51 1.46 1.44 1.39 1.36 0.94 0.93 0.92
0.01
(H3C)2N
N(CH3)2
C8H17
Ph
N
B
1l
2.14 2.17 2.26 5.94 2.24 4.08 16.00 7.75 3.97 11.90 4.33 4.41 20.24 6.25
8.0 7.5 7.0 6.5 6.0 5.5 5.0 4.5 4.0 3.5 3.0 2.5 2.0 1.5 1.0 0.5 0.0
ppm

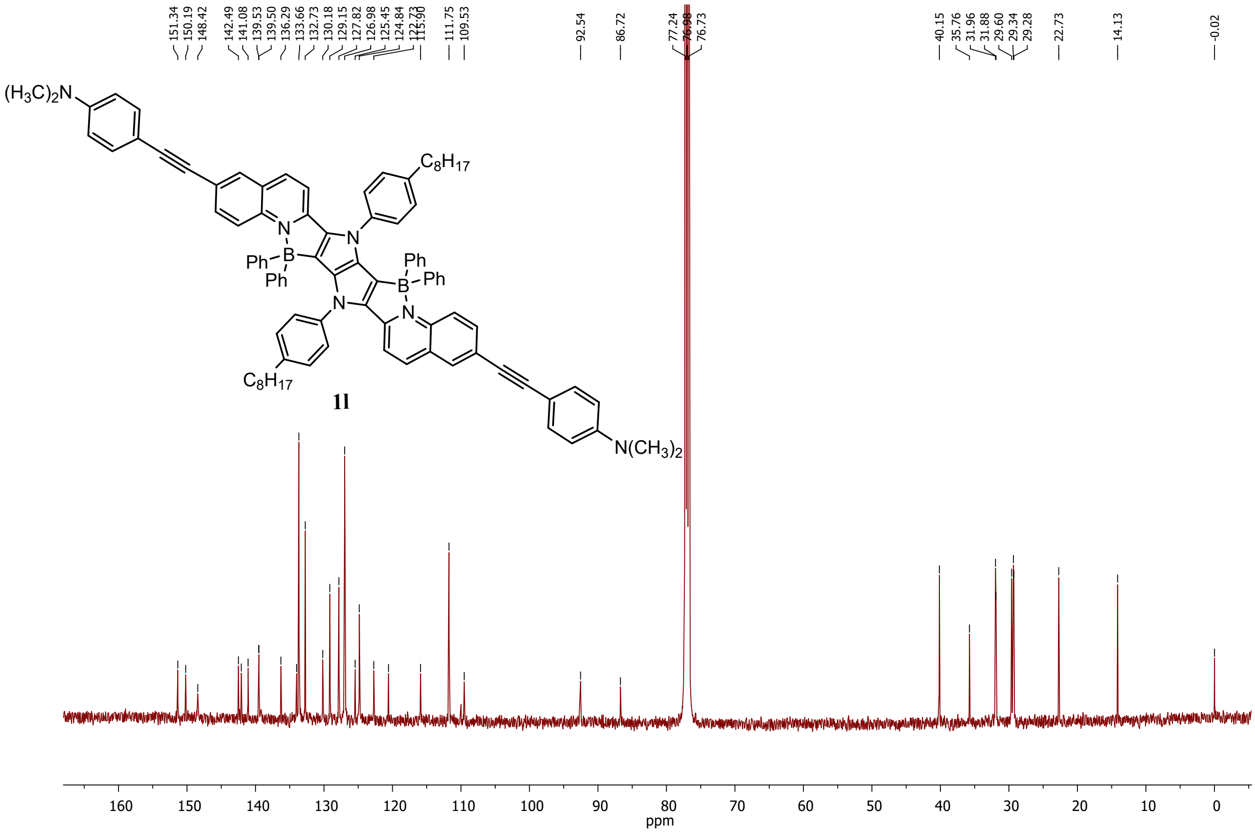
151.34
150.19
148.42
142.49
141.08
139.53
139.50
136.29
133.66
132.73
130.18
129.15
127.82
126.98
125.45
124.84
122.73
115.90
111.75
109.53
92.54
86.72
77.24
76.98
76.73
40.15
35.76
31.96
31.88
29.60
29.34
29.28
22.73
14.13
-0.02
(H3C)2N
C8H17
Ph
N
B
N(CH3)2
1l
ppm

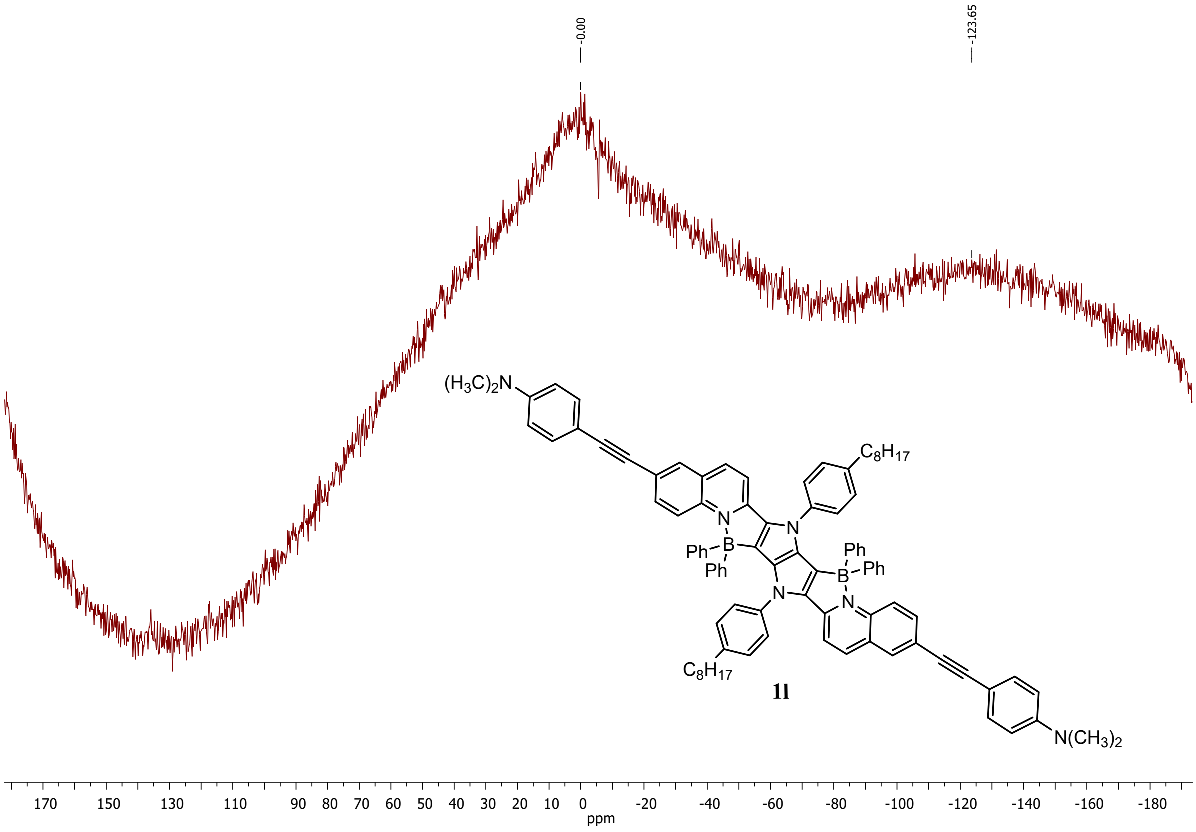
-0.00
-123.65
(H3C)2N
C8H17
Ph
N
B
N(CH3)2
1l
ppm

[1] M. J. Frisch et al. Gaussian 16.A.03 Wallingford, CT, 2016.
[2] Y. Zhao, D. G. Truhlar, *Theor. Chem. Acc.* 2008, **120**, 215–241.
[3] J. Tomasi, B. Mennucci, R. Cammi, *Chem. Rev.* 2005, **105**, 2999–3094.
[4] R. Cammi, B. Mennucci, *J. Chem. Phys.* 1999, **110**, 9877–9886.
[5] F. Santoro, D. Jacquemin, *Wires Comput. Mol. Sci*. 2016, **6**, 460–486.
[6] (a) J. Cerezo, F. Santoro, FCClasses 3.0, http://www.pi.iccom.cnr.it/fcclasses; (b) F. Santoro, R. Improta, A. Lami, J. Bloino, V. Barone, *J. Chem. Phys.* 2007, **126**, 084509.
[7] K. Aidas, et al., *WIREs Comput. Mol. Sci.* 2014, **4**, 269–284.
[8] T. Yanai, D. P. Tew and N. C. Handy, *Chem. Phys. Lett*., 2004, **393**, 51–57.
[9] B. Le Guennic and D. Jacquemin, *Acc. Chem. Res*., 2015, **48**,
530.
[10] M. Tasior, O. Vakuliuk, D. Koga, B. Koszarna, K. Górski, M. Grzybowski, Ł. Kielesiński, M. Krzeszewski and Daniel T. Gryko, *J. Org*. *Chem*., 2020, **85**, 13529.
